# Unveiling the Power of Proximity of Prevalent Fe-Based Tandem Catalysts in CO_2_ Hydrogenation via Modified Fischer–Tropsch: Crucial Relations toward Industrialization

**DOI:** 10.1021/acs.chemrev.4c00697

**Published:** 2025-10-22

**Authors:** Sara Najari, Samrand Saeidi, András Sápi, Zoltán Kónya, Gábor A. Somorjai

**Affiliations:** † Department of Applied and Environmental Chemistry, Interdisciplinary Excellence Centre, 37442 University of Szeged, Rerrich Bela ter 1, Szeged H-6720, Hungary; ‡ Department of Chemistry, University of California, Berkeley, California 94720, United States

## Abstract

CO_2_ reduction using renewable H_2_ represents an emerging approach for minimizing dependency on fossil fuels and reducing the carbon footprint while providing chemicals and fuels. In this context, CO_2_ hydrogenation using Fe-based oxide, which exhibits outstanding capabilities in both reverse water gas shift (RWGS) and Fischer–Tropsch synthesis (FTS) reactions, integrated with zeolite has been a promising method for heavy hydrocarbon (C_5+_) production. This review investigates the critical roles of promoter, zeolite topology and acidity, and synthesis methods in optimizing product distribution and their contributions to active site proximity. It has been found that the catalyst integration manner and the interaction between the basic sites of Fe-based oxide and the acidic sites of zeolites significantly influence catalytic performance. In addition, the proximity of active sites, a crucial factor in tandem catalysis, can be controlled via different catalyst synthesis methods, dispersion on mesoporous supports, or using encapsulated structures that can provide the confinement effect while guiding the reaction sequence. Furthermore, the choice of alkali promoters (Na vs K) is very important since each can alter electronic properties, reduction behavior, and hydrocarbon distribution due to different electronegativity and ionic radii. While Na could hamper all reduction steps and diffuses into bulk iron oxide, K remains mainly on the surface, increasing electron density and facilitating iron carbide formation. Besides, integrating spectroscopic imaging techniques with proximity metrics will enhance the understanding of active site spatial distribution. To bridge the gap between lab-scale results and industrial applications, advanced computational methods coupled with artificial intelligence (AI) and machine learning (ML) techniques are required to monitor and analyze catalyst behavior and optimize large-scale production. The findings of this study provide a comprehensive understanding of catalyst design principles with emphasis on the importance of the proximity of active sites, offering insights for the next generation of efficient CO_2_ hydrogenation catalysts for industrial-scale fuel production.

## Graphical abstract

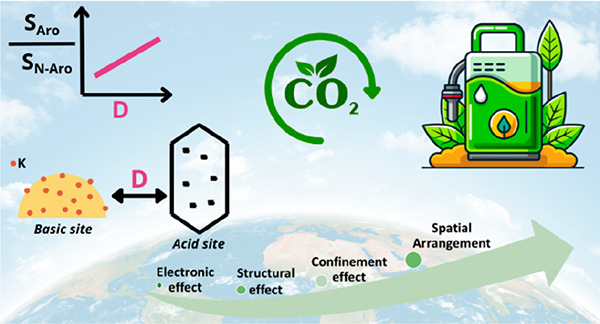

## 1 Introduction

The utilization of CO_2_ has attracted significant research attention due to the growing interest in using renewable resources and converting them into various forms of energy, such as electricity or chemicals.^1,2^ This emerging potential, driven by current and anticipated cost reductions as well as increased durability of CO_2_ utilization technologies, provides an opportunity for CO_2_ transformation into valuable carbon-based products. Converting significant amounts of CO_2_ into useful chemicals brings us closer to establishing a circular carbon economy that mimics natural processes.^3,4^ Additionally, the utilization of CO_2_ presents the opportunity to reduce greenhouse gas emissions into the atmosphere and decrease the extraction of fossil carbon, as some of it can be replaced with recycled carbon.^5,6^ In the coming years, fuels will remain vital, and CO_2_ hydrogenation represents a promising and sustainable fuel production strategy. Through thermo-catalytic reactions, CO_2_ and renewable H_2_ ^7^ can be employed to produce heavy hydrocarbons (C_5+_) that closely resemble the fuels commonly used today, such as gasoline, diesel, liquefied natural gas (LNG), and jet fuel.^8–10^

It has been indicated that hydrogenation of CO_2_ includes a wide variety of reactions exhibiting challenging issues, which necessitate an in-depth understanding of various aspects of heterogeneous catalysis, including the role of promoters or second metals, the support properties, and the characteristics of zeolite.^11^ Moreover, recently, it has been found that the proximity of active sites within the catalyst particle, the confinement effect, and the spatial distribution of active sites in the reactor are of paramount importance. In fact, the transport of intermediates and products, and consequently, the distribution of final products, can be controlled via an appropriate configuration of active species in CO_2_ hydrogenation.^12,13^

Due to the substantial number of publications and widespread interest in the CO_2_ hydrogenation process over the last few decades, numerous outstanding reviews have emerged, covering the advancements in catalyst development, process intensification, and reaction mechanisms for converting CO_2_ into various chemicals and fuels.^14–20^ However, with the rapid progress of this process toward industrialization and a notable absence of reviews addressing the significance of active site proximity and configuration, there is a pressing need to develop a systematic and critical investigation to understand the proximity effect in CO_2_ catalysis to produce desired product distribution.

To this end, a comprehensive understanding and a well-rounded summary of the proximity of active phases within Fe-based catalysts, confinement effect, various integration methods of Fe-based catalysts and zeolites in the reactor, as well as the contributions of alkali promoters and the active site proximity on the product distribution, are presented. Hence, to avoid redundancy and duplication with existing reviews, this paper primarily focuses on the integration of Fe-based catalysts with zeolites, with a specific emphasis on the role of active site proximity on the distribution of heavy hydrocarbons (C_5+_). This review focuses on the latest advancements in this domain, with a particular emphasis on the roles of promoter type and zeolite Brønsted acidity. While doing so, we meticulously assess the most pertinent publications from 2017 to 2024, acknowledging the pioneering contributions of earlier works.

The current study starts with the fundamental concepts in CO_2_ hydrogenation, followed by a discussion of the principles of catalyst design. Then, the crucial role of promoters, which induce basic properties to the catalyst, on CO_2_ hydrogenation performance in terms of both activity and C_5+_ selectivity is addressed. In the next section, zeolite topology and its acidity, as the active oligomerization sites of the catalyst, are studied comprehensively to provide a clear insight into their contributions to the distribution of heavy hydrocarbons. Moreover, the factors contributing to the proximity of Fe-based active phases are studied. Following the roles of Fe-based oxide and zeolite, we analyze their integration mode and proximity of active sites on the product distribution, providing the possibility to design catalysts for the proper energy sector and industrial applications. In addition, by examining the aforementioned parameters, a roadmap is proposed to unlock the potential of alkali-promoted Fe-based catalysts for tuning the aromatic to nonaromatic ratio by changing the proximity of active sites. Besides exploiting C_5+_ yield vs C_5+_ STY correlation, considering the critical role of GHSV, more detailed insights into the performance of various catalysts for large-scale purposes are obtained. In addition, the methods of evaluating proximity in heterogeneous catalysis in lab- and large-scales are addressed, and their limitations are highlighted. Moreover, some remarks and suggestions regarding the complexities and issues associated with this process for industrialization are addressed. Finally, a method is proposed based on advanced computational tools, coupled with AI/ML, to optimize catalyst integration and process at a large scale, exploiting proximity data obtained at the lab scale. Figure 1 illustrates the contributing factors to the proximity of oxide and zeolite, which clarifies the roadmap of this review paper.

**Figure 1:**
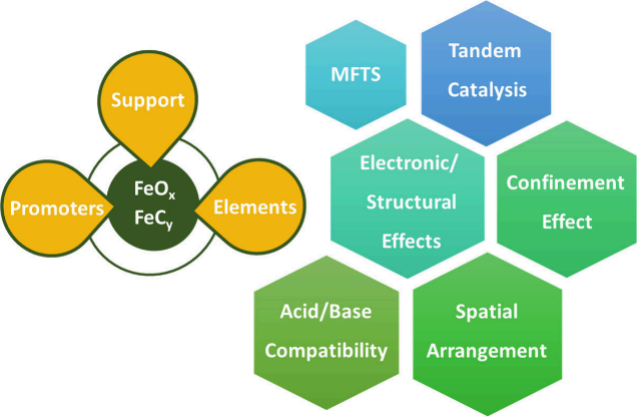
IIustration of the contributing factors to the proximity between oxide and zeolite in this research (designed by authors).

## 2 Fundamentals of CO_2_ Hydrogenation

Effective CO_2_ hydrogenation to chemicals and fuels has been one of the most vital challenges of catalysis. This challenge attracted much attention due to the urgent need to mitigate CO_2_ emissions and develop sustainable energy cycles. Nevertheless, the thermodynamic and kinetic obstacles of this reaction made this accomplishment difficult. Therefore, it is essential to understand the fundamental concepts in order to properly address these challenges.

### 2.1 Thermodynamic Challenges

Although the C=O bond in CO_2_ is quite strong, the overall CO_2_ hydrogenation process is exothermic, since the total energy released by forming the C–C and C–H bonds in hydrocarbons and O-H in H_2_O, exceeds the energy required for breaking C=O and H–H bonds in reactants. Thus, the formation of heavier hydrocarbons decreases overall enthalpy, as these molecules have more bonds, which makes CO_2_ hydrogenation more enthalpically favorable. On the other hand, the conversion of gaseous reactants to liquid/solid products (phase transition) decreases entropy. In addition, forming fewer large molecules (for instance, one or two products can be formed from four reactant molecules) that have fewer degrees of freedom than reactant molecules further reduces entropy. Therefore, the entropy penalty becomes large in hydrocarbon production via CO_2_ hydrogenation. The balance between enthalpy gain and entropy cost determines the thermodynamic favorability of each reaction. Since in CO_2_ hydrogenation, the formation of heavy hydrocarbons results in reduced enthalpy and entropy, the spontaneity of the reaction is determined by Gibbs free energy change (Δ*G* = Δ*H* – *T*Δ*S*). Besides, the role of temperature cannot be neglected in hydrocarbon production. At low temperatures, the enthalpy term is the dominant factor for heavy hydrocarbon formation. In contrast, at high temperatures, the enthalpy benefit can be outweighed by the entropy penalty, impeding the formation of long-chain hydrocarbons. Therefore, volume reduction and exothermicity show that hydrocarbon formation is favored by low temperatures and high-pressure.^21^

The RWGS reaction is endothermic in nature and thus favored by higher temperatures, while increasing pressure does not have any effect on the reaction, as the number of molecules is similar on both sides. In CO_2_ hydrogenation, C_2_–C_4_ selectivity is almost zero (Figure 2 (a)), while the process is highly selective toward CO and CH_4_ over a wide range of temperatures and CO_2_/ H_2_ ratio (Figure 2 (b)). It can be observed that a higher H_2_ partial pressure results in higher CO_2_ conversion and CH_4_ formation. However, CO dominates CH_4_ as temperature increases. Excluding CO and CH_4_, it can be observed that C_2_H_6_ is more favorable compared to other C_2_–C_4_ hydrocarbons (Figure 2 (c)), and high CO_2_ conversions are theoretically possible at moderate temperatures and pressures (Figure 2 (d)).^22^ It was found that, in the FT process, olefins and paraffins do not follow similar thermodynamic trends. The formation of lighter paraffins is more exothermic than that of heavier ones, while the production of light olefins is less exothermic than that of heavier ones. The equilibrium CO_2_ conversion and olefins selectivity for 1 and 50 bar can be observed in Figure 2 (e) and (f).^21,23^ To achieve high selectivity toward heavy hydrocarbons, appropriate catalysts should be employed to suppress CO and CH_4_ formation.

**Figure 2:**
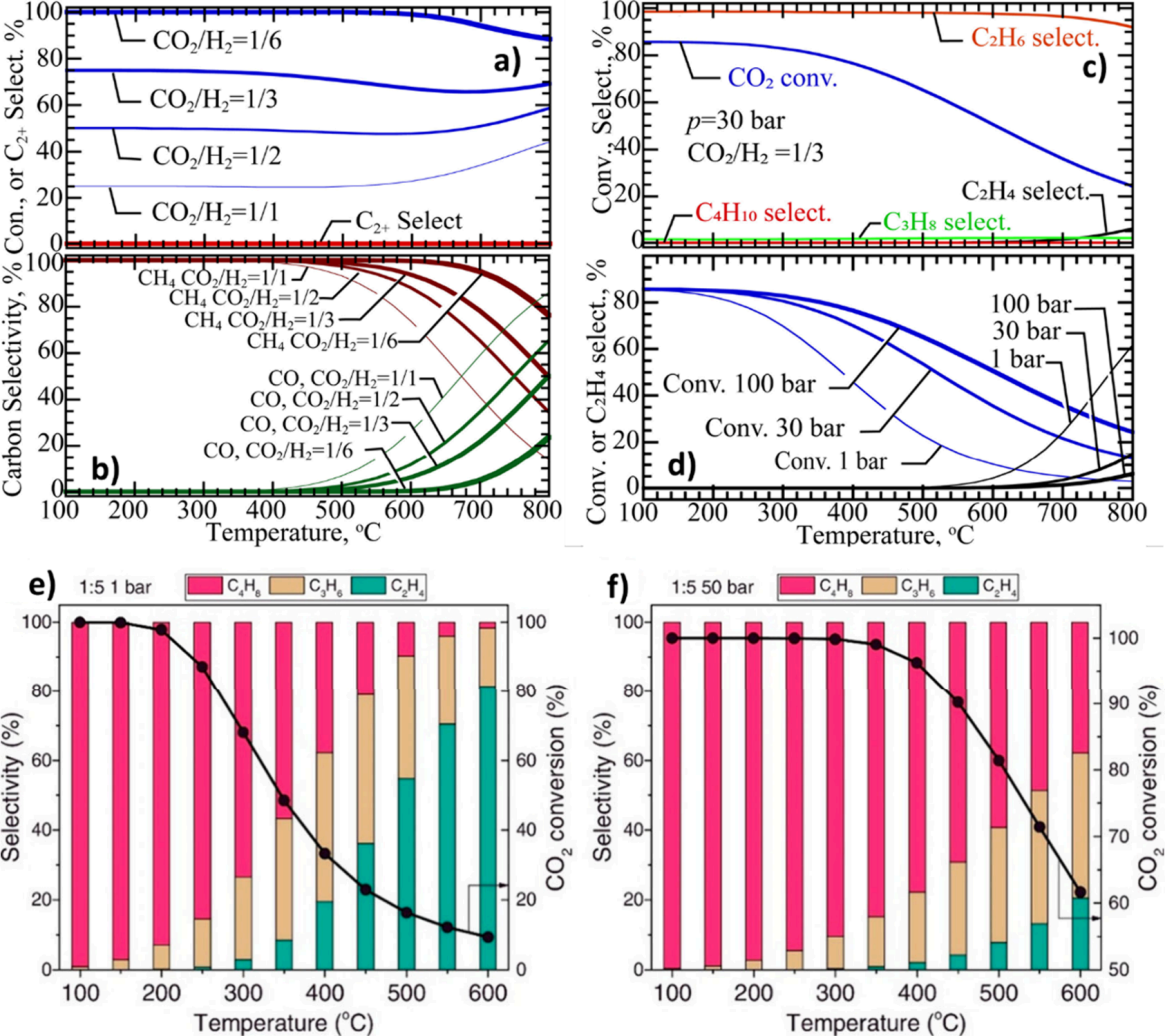
Equilibrium predictions of CO_2_ conversion to CO, CH_4_ and C_2_–C_4_ hydrocarbons: **a)** C_2_–C_4_ selectivity and CO_2_ conversion (*P* = 30 bar). **b)** Effect of CO_2_/H_2_ ratio on CO and CH_4_ selectivity, Equilibrium predictions of CO_2_ conversion to C_2_–C_4_ hydrocarbons excluding CO and CH_4_. **c)** CO_2_ conversion and C_2_–C_4_ olefin selectivity (*P* = 30 bar, CO_2_/H_2_ = 1/3). **d)** The impact of pressure and temperature on C_2_H_4_ selectivity. Reproduced with permission from ref 22. Copyright 2022, John Wiley and Sons. CO_2_ conversion and C_2_–C_4_ olefins selectivity at CO_2_/H_2_ = 1/5 **e)** 1 bar and **f)** 50 bar. Reproduced with permission from ref 23. Copyright 2016, Elsevier.

### 2.2 Thermocatalytic CO_2_ Hydrogenation

The CO_2_ hydrogenation reaction can be categorized into two primary pathways depending on the intermediates involved in the reaction: (i) the methanol-mediated pathway, where methanol acts as an intermediate, and (ii) the modified Fischer–Tropsch synthesis (MFTS) pathway, including reverse water gas shift (RWGS) and FTS reactions to produce hydrocarbons^24–26^ (Figure 3 (a)). The MFTS route enables direct production of hydrocarbons via surface-bond CO and CH_x_ species, in contrast to the methanol-mediated pathway. Moreover, the wide operating temperature of the MFTS pathway allows using the catalyst at moderate temperatures.^22^

**Figure 3:**
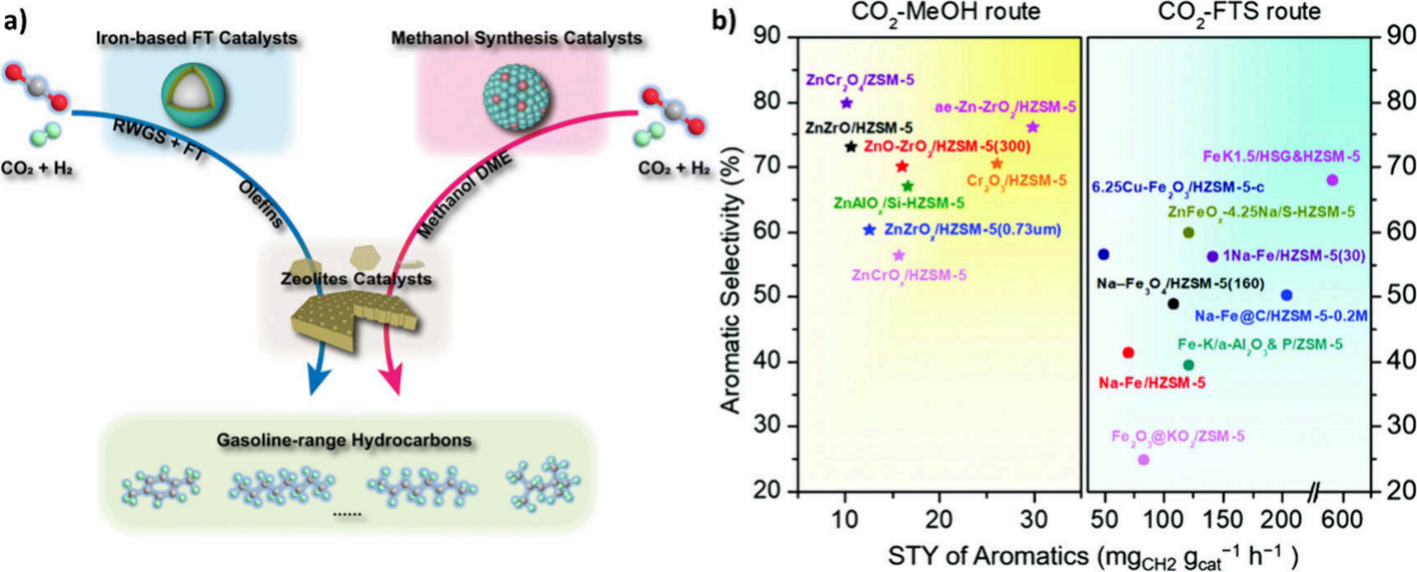
**a)** Schematic illustration of two main routes of CO_2_ hydrogenation. Reproduced with permission from ref 26. Copyright 2023, RSC Publishing under [CC BY 3.0]. **b)** Aromatic selectivity vs STY of CO_2_ hydrogenation to C_5+_ hydrocarbons. Reproduced with permission from ref 46. Copyright 2021, RSC.

Iron (Fe)-based catalysts are commonly used in CO_2_ hydrogenation reactions due to their excellent capability to catalyze both RWGS and FT reactions. Iron is abundant and relatively cost-effective compared to other metals. These advantages make Fe-based catalysts highly attractive for large-scale applications, including CO_2_ hydrogenation reactions.^27–29^ Furthermore, the Fe-based catalyst performance can be adjusted by altering its synthesis method, introducing promoters and support materials, and integrating it with zeolites.^30^ This flexibility enables the optimization of catalytic properties, including activity, selectivity, and stability, to meet specific process requirements.^31^ Previous results have shown that Fe-based catalysts can be tailored or modified to control selectivity toward desired products, potentially playing a substantial role in driving the progress of sustainable technologies for CO_2_ utilization and the mitigation of greenhouse gas emissions.^32–34^ Moreover, Fe-based catalysts demonstrated good catalytic stability during CO_2_ hydrogenation, even under harsh reaction conditions, without significant deactivation or loss of catalytic performance. This remarkable stability leads to prolonged catalyst lifetimes, reducing the necessity for frequent catalyst regeneration or replacement.^35,36^

In the RWGS reaction, CO_2_ is activated via H_2_ and converted to CO and H_2_O in the presence of basic Fe-based oxide,^37,38^ while iron carbides (Fe_5_C_2_, Fe_3_C, etc.) formed in situ under reduction/reaction conditions are responsible for the FTS reactions.^39–41^ Different hydrocarbons can be formed in the FT reaction via the hydrogenation of CO, which acts as a feedstock for the FTS reaction, through a series of complex reactions.^42–44^ Moreover, it was recently revealed that Fe-based catalysts exhibited higher aromatic STY than those used for the MeOH-mediated route^26,45^ (Figure 3 (b)).

The main disadvantage of the MFTS is the thermodynamic limit of C_2+_ formation since CO and CH_4_ are the thermodynamically favored products. However, due to their low energy density and end-use value, higher-value hydrocarbons are necessary to justify the costs of CO_2_ capture and H_2_ production.^22^ Many studies have shown that the equilibrium limit could shift according to Le Chatelier’s principle by removing the produced water. In this regard, mixing the catalyst with a hydrophilic adsorbent has been found to be a promising approach.^47^

### 2.3 Mechanistic Insight and Mechanisms

There are limited Density Functional Theory (DFT) studies that have been carried out to map out the complete reaction mechanisms of CO_2_ conversion to multicarbon products via the FT reaction, despite the fact that computational approaches have been extensively used to gain insight into the reaction mechanism of CO_2_ conversion to C_1_/C_2_ products. The complexity of the reaction network of CO_2_ conversion, the heterogeneity of the multicomponent catalysts, the change in phase of catalysts due to reaction conditions, and the presence of promoters make realistic DFT modeling of the detailed mechanism of CO_2_ conversion very challenging. According to the CO_2_-MFTS pathway, it is believed that the CO_2_ conversion to higher hydrocarbon products occurs via the coupling of the RWGS and FTS reactions, where CO_2_ is first converted to CO via the RWGS reaction, which undergoes FTS to produce hydrocarbons.^48^

The RWGS over Fe-oxide has been considered a redox cycle consisting of the reduction of Fe^3+^ to Fe^2+^ and oxidation of Fe^2+^ by CO_2_ to form CO, which can lead to the carbonate formation as an intermediate (Figure 4 (a)). Besides, the formed O vacancies through surface dehydration act as active centers for CO_2_ activation, which results in CO formation and Fe^3+^ restoration.^49^ Han et al.^50^ classified the RWGS reaction over Fe-based catalysts into three pathways: the direct CO_2_ dissociation to CO*, the COOH*-mediated pathway, and the HCOO*-mediated pathway. DFT calculations found that the direct mechanism was the most favorable among other pathways over all the Fe-based catalysts examined.^50^ Investigating the CO_2_ activation on different facets of metallic Fe, it was found that Fe(111) exhibited a high ability to activate CO_2_. It was also shown that Fe(100) can activate CO_2_ via direct dissociation.^51^ Nie et al.^51^ demonstrated that CH* was the primary intermediate on Fe(100), and CH_4_ formation was favored kinetically owing to lower energy barriers compared to that of C–C coupling.^51^ Recently, the same group studied CO_2_ activation on different facets of Fe_5_C_2_. It was shown that the (510) facet exhibited a low activation energy for direct CO_2_ dissociation, while the H-assisted pathway (via HCOO*) was preferred on the (111) and (100) facets.^52^ Furthermore, using DFT calculations, Wang et al.^53^ explored the CO_2_ hydrogenation mechanism on the Fe_5_C_2_(510) surface for CH_4_ and C_2_H_4_ formation. It was revealed that CH* species was the key intermediate; however, the high coverage of O* species on the surface, resulting from CO_2_ activation, occupied the main active sites and hindered C_2_ formation via C–C coupling. This O* accumulation could negatively affect catalyst stability.^53^ However, DFT calculations showed that, on Fe_5_C_2_ (111), the O* could be readily removed as H_2_O, which maintains catalyst stability.^54^ Adsorption and dissociation of H_2_ and CO on the surface carbides can form CH* and CH_2_*, which can be oligomerized to form higher hydrocarbons (Figure 4 (b)). The chain initiation caused by CO insertion follows the insertion mechanism; however, chain propagation, which is induced by CCH coupling, occurs through the carbide mechanism. The combined action of both mechanisms results in the FTS reaction.^55^ Figure 4 (c) illustrates the CO_2_-MFTS mechanism; in the first step, CO_2_ is adsorbed on Fe^2+^, oxidizing it to Fe^3+^. Meanwhile, O radicals and C=O groups formed, and H_2_ dissociation resulted in H* formation. In the third step, C=O is attacked by H*, followed by the formation of CO and H_2_O via OH attacking (steps 3a and 4). It can be observed that formaldehyde and alcohol can be formed (from 5a to 7b) if H* attacks the group and the C=O was formed after step 3a dissociates from H_2_. The key species for C–C coupling, which is considered to be Fe^3+^-CH_2_*, is formed after step 7a. The CO_2_ can be attacked by CH_2_* and follow the same pathway to form olefin and reduce Fe if the H can be eliminated from the carbon chain.^56^ The schematic illustration of CO_2_-MFTS and the main intermediates is shown in Figure 4 (d).

**Figure 4:**
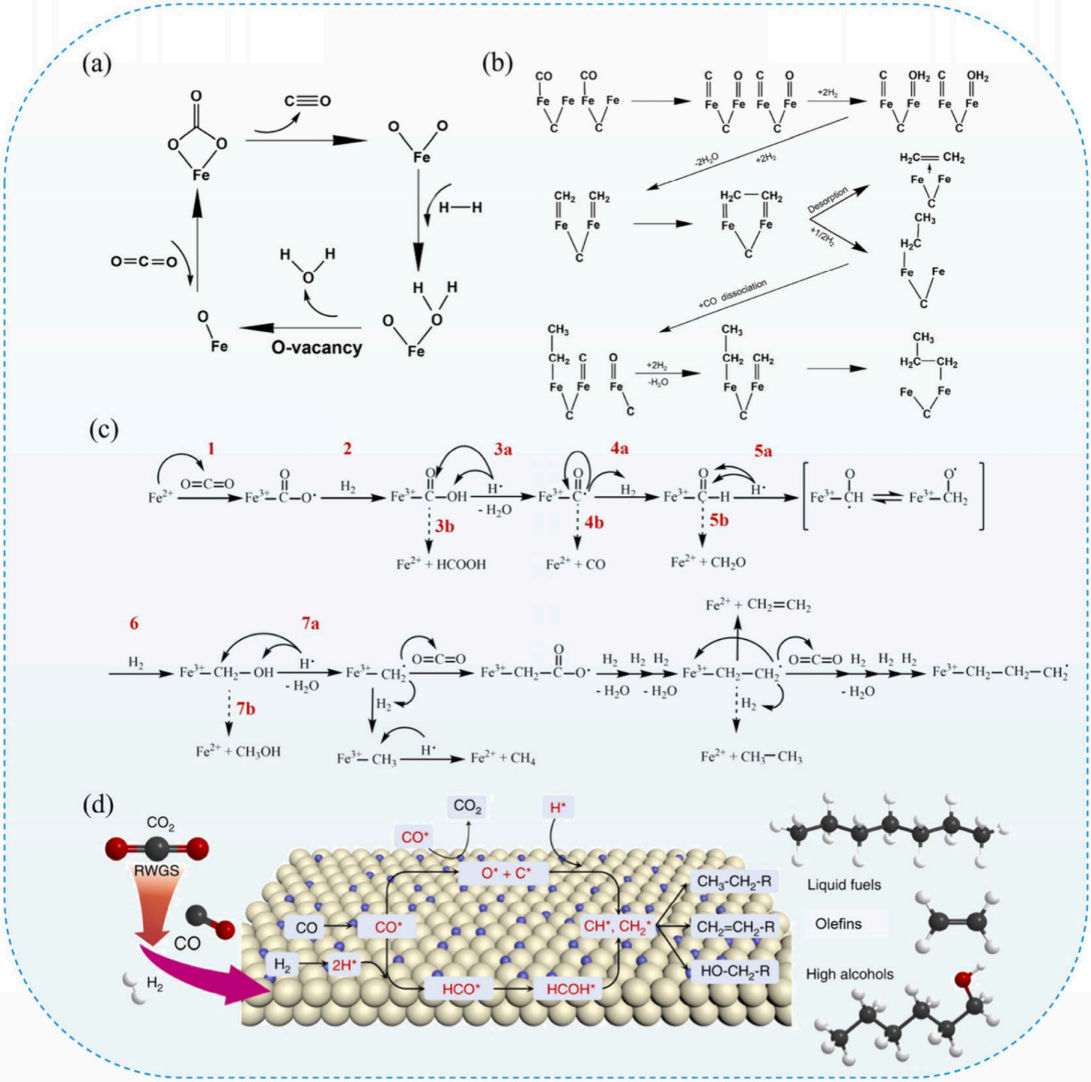
Schematic illustration of **a)** The RWGS mechanism, **b)** the surface carbide mechanism for FTS reaction, **c)** the overall CO_2_ hydrogenation mechanism (the dotted arrows show secondary routes, while solid arrows represent primary routes), **d)** the CO_2_-FTS mechanism. Reproduced with permission from ref 56. Copyright 2024, Elsevier.

## 3 Principles of Catalyst Design

The design of the catalyst is an important factor in optimizing CO_2_ hydrogenation performance, such as activity, selectivity, and stability under various reaction conditions. In addition to addressing thermodynamic constraints, an effective catalyst should provide coordinated and efficient reaction steps. In this regard, tandem catalysis with special attention to the spatial arrangement of active sites should be explored as a determining factor in catalyst design.

### 3.1 Tandem Catalysis

Production of heavy hydrocarbons via coupling the endothermic RWGS and exothermic FTS reactions would be challenging for single-step catalysis due to difficulties in tuning product distribution and conflicting reaction conditions. Tandem catalysis, where the products of one reaction (RWGS) are consumed as the feed of other reactions (FTS), can reduce reaction temperature and enhance CO_2_ conversion according to Le Chatelier’s principle.^57^ Therefore, by tuning the spatial arrangement of the active sites, maintaining the required intermediate conversions, and keeping the balance between the heat requirements of the endothermic and exothermic steps, tandem catalysis can address the mentioned issues.^58^ In this realm, tandem catalysts primarily made up of coupling active metal oxides and zeolites have attracted much attention due to their potential in heavy hydrocarbons and fuel production via CO_2_ hydrogenation.^59–61^

Tandem catalysis, involving at least two different mechanisms under the same conditions for converting reactants to products, relies on the precise regulation of the spatial distribution and connectivity of active sites.^62,63^ Furthermore, tandem catalysts allow for the sequential coupling and mediation of individual steps in multistep reactions at the nanoscale, resulting in the formation of a target product.^64,65^ This approach enables the intensification of macroscale and microscale processes at the nanoscale. The reaction rate depends on the individual step rate at the corresponding sites as along with the transfer of the intermediates via diffusion or spillover.^66,67^ To effectively control tandem catalysis, the movement of reactive intermediates across various catalytic sites needs to be controlled precisely.^68^

### 3.2 Proximity Concept

The spatial distance between the two functional groups or active sites, also known as proximity in tandem catalysis, has a profound impact on both CO_2_ hydrogenation efficiency and product distribution.^69–71^ In other words, different integration methods of active sites can affect CO_2_ conversion and alter hydrocarbon selectivity toward the desired products.^72,73^ For many years, Weisz’s intimacy criterion,^74^ which suggests that closer proximity of active sites leads to better performance, was commonly used to optimize the spatial configuration of these sites. However, it is essential to note that this criterion, which was validated at the micron level, is primarily associated with the catalytic activity of metals/metal oxides, and the effect of proximity between different metal oxides and zeolitic acid sites at the nanoscale and on product distribution has not been well explained and elucidated.^75,76^ The close proximity of active sites can accelerate the second reaction due to the high concentration of intermediates, which are the products of the first reaction. On the other hand, undesired interactions between the active sites may prohibit the second reaction, leading to undesired byproducts or catalyst deactivation.^77^ Therefore, accurate control over how intermediates interact with the second site is crucial for achieving high selectivity in integrated tandem catalysis.^78^

Employing the treasure map metaphor, Figure 5 schematically illustrates the effect of the detrimental interactions arising from the inappropriate proximity of Fe-based oxide and zeolite on the reaction products. The treasure chest represents the desired products, obtained by guiding the reactants and intermediates through the appropriate reaction path (depicted as islands) when oxide and zeolite are located at the optimum distance, illustrating the importance of proximity at tandem catalysis.

**Figure 5:**
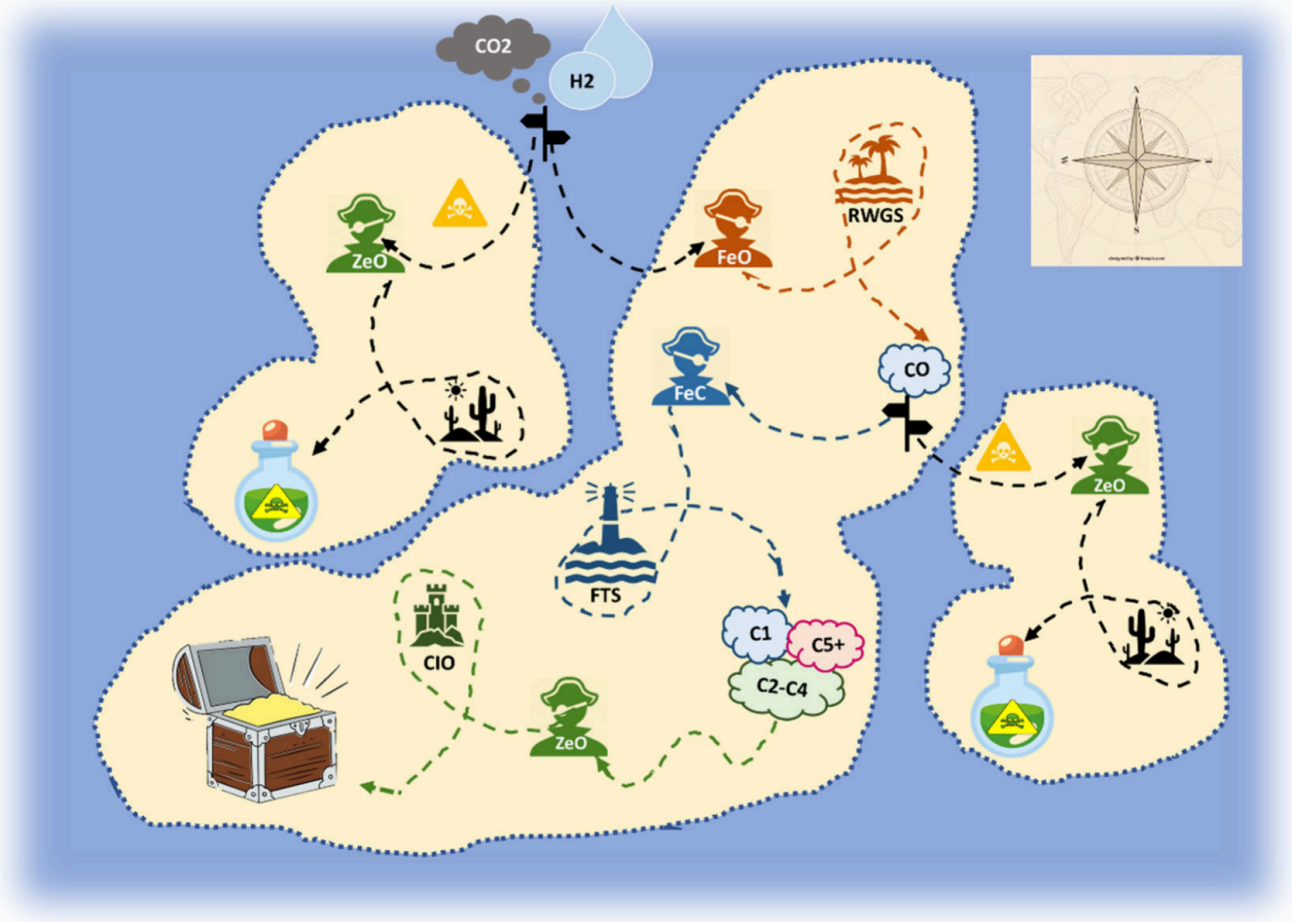
Schematic representation of the reaction landscape using a treasure map metaphor. The pathways illustrate how the appropriate spatial arrangement of Fe-based oxide and zeolite diminishes detrimental interactions caused by the misalignment of oxide and zeolite proximity. The treasure chest represents the desired products, which are achieved when oxide and zeolite are situated at an optimal proximity (Designed by the authors).

The reactions that take place over bifunctional catalysts involve a series of interlinked transport and reaction steps, each with its own specific time and size scale. This complex system can be represented by a resistive circuit in Figure 6 that includes transport and transformation steps for each active site.^79^ Accordingly, the CO_2_ hydrogenation reaction involves the gas-phase reactants (CO_2_ and H_2_) being transported to the surface of the metal oxide through diffusion (τ_G_), where they undergo RWGS (τ_G_) and other reactions (τ_O_) such as methanation over Fe-oxide, while CO advect/diffuse (τ_Dr_/τ_Da_) to the in situ formed Fe-carbide, and CO-FT proceeds (τ_FT_). The formed intermediates over the Fe-oxide and carbide mainly consist of light olefins and paraffins that advect/diffuse (τ_Dr_/τ_Da_) to the zeolite reactive domain, where a secondary set of steps occur. These steps generally include reactions on the BAS of zeolite (τ_S_), intraparticle diffusion (τ_Da_), and heavy hydrocarbon formation (τ_HC_), where light hydrocarbons participate in oligomerization, isomerization, or cracking reactions, which may undergo cyclization with further dehydrogenation (aromatization) steps. The secondary byproduct pathways, such as paraffin formation via olefin hydrogenation, are also expected in both domains.^79^

**Figure 6:**
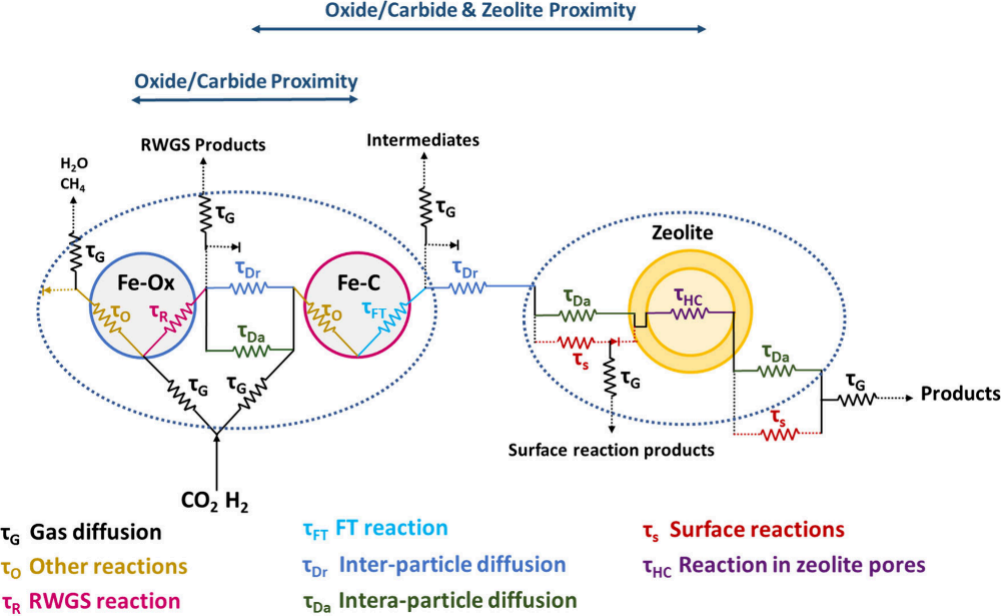
Schematic representation of transport and reaction time-scales based on resistive circuit concept, reconceptualized and redrawn with permission from Nezam et al.^79^ Copyright 2023, ACS under [CC-BY 4.0].

The nature of these active sites determines how they should be coupled to result in the highest catalytic performance. However, it is essential to note that while process intensification is usually more effective at the nanoscale, specific catalysts may be better integrated through other integration methods, such as physical mixing, which can create micro- to millimeter-scale spacing between active sites caused by compatibility issues within different active phases.^80^ The decision to use single reactor vessels for tandem reactions using integrated tandem catalysts, mixed catalysts, or dual-bed is typically driven by the desire to create processes that are both economically advantageous and environmentally friendly, by avoiding the need for extra cooling and separation in two-stage reactor configurations.^81,82^ Moreover, the thermodynamic characteristics of the individual reaction steps in CO_2_ hydrogenation result in different reaction temperature windows (RTW) for each reaction due to the endo- and exothermic nature of RWGS and FTS reactions, respectively. Where the difference in RTW results in a positive Gibbs free energy of the overall reaction, two-stage reactors should be used.^57^ However, a recent investigation by Hos et al.^83^ found that using Fe-based catalysts for tandem CO_2_ hydrogenation to liquid fuel in a single reactor vessel does not offer any significant advantages over a two-stage reactor in spite of fewer recycling stages and less separation energy. One crucial factor affecting the economic feasibility of various methods is how well individual catalysts perform, particularly in terms of their selectivity and stability. Therefore, achieving exceptional overall performance, encompassing both activity and selectivity, is crucial to reduce energy consumption for separation within a single reactor, as opposed to employing a two-stage reactor configuration.^67^

## 4 The Role of Promoters and Additional Elements

Even though Fe-based catalysts have advantages in converting CO_2_ into valuable hydrocarbons such as light olefins or liquid fuels, the effectiveness of using CO_2_ is limited due to the chemical equilibrium in the CO_2_ hydrogenation process. Suppose the step that controls the rate of C–C coupling is facilitated. In that case, the driving force for RWGS can be enhanced by consuming intermediate CO, thereby increasing the efficiency of the carbon element and promoting the formation of hydrocarbons. Based on this principle, a different metal element that has greater activity for C–C coupling but does not alter RWGS can be incorporated into the Fe catalysts as a dopant or promoter.^84,85^

The role of various metal promoters in CO_2_ hydrogenation was investigated by Li et al.^86^ via synthesis of Fe/M = 40/1 (atomic ratio), where M can be Li, Na, K, Rb, Cs, Ca, Co, Al, Mg, Cu, Zn, and Mn. As illustrated in Figure 7 (a, b), the addition of monovalent alkali metals (as promoters) to the Fe_2_O_3_ resulted in the formation of more C_2_–C_4_ olefins and C_5+_ hydrocarbons than the unpromoted Fe_2_O_3_. In contrast, the introduction of divalent or trivalent metals led to the formation of more CH_4_ and C_2_–C_4_ paraffins. According to TEM and SEM images, the introduction of alkali metals reduced the iron oxide particle size, increased the BET surface area, and thus facilitated the reduction of Fe_2_O_3_ to Fe_3_O_4_ via tuning the electronic state of the catalyst.^86^

**Figure 7:**
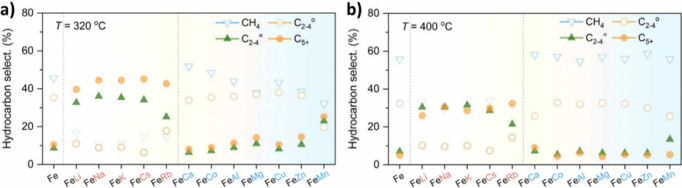
Effect of metal promoters on the product distribution of CO_2_ hydrogenation at **a)** 320 °C, **b)** 400 °C (reaction operating conditions: *P* = 3 MPa, GHSV = 6000 mL/g h). Reproduced with permission from ref 86. Copyright 2023, Elsevier.

### 4.1 Monovalent Elements

Evidently, alkali metals such as Na and K affect the basicity, reduction behavior, and carburization of iron oxides.^87–89^ The addition of appropriate amount of alkali metals enhances the stability of Fe-based catalysts by protecting their active sites from potential hydrogenation or oxidation, which could otherwise result in the loss of these sites.^90,91^ However, it was confirmed that Na strongly retards the reduction of all forms of iron oxides, while K partially inhibits their reduction. In other words, K restrained the initial reduction steps of Fe_2_O_3_ to Fe_3_O_4_, while it was ineffective in limiting Fe_3_O_4_ reduction to FeO and metallic Fe as much as Na could.^92,93^ In situ X-ray diffraction (XRD) measurements over alkali-promoted Fe/SiO_2_ showed that alkali promoters suppressed the first reduction step (Fe_2_O_3_ to Fe_3_O_4_), while the inhibitive effect was reduced with increasing alkali atomic number, as shown in Figure 8 (a) and Figure 8 (b) for Na- and K-promoted samples, respectively.^94^ However, the magnitude of these effects depends on support, promoter form and loading, and gas composition.^92^

**Figure 8:**
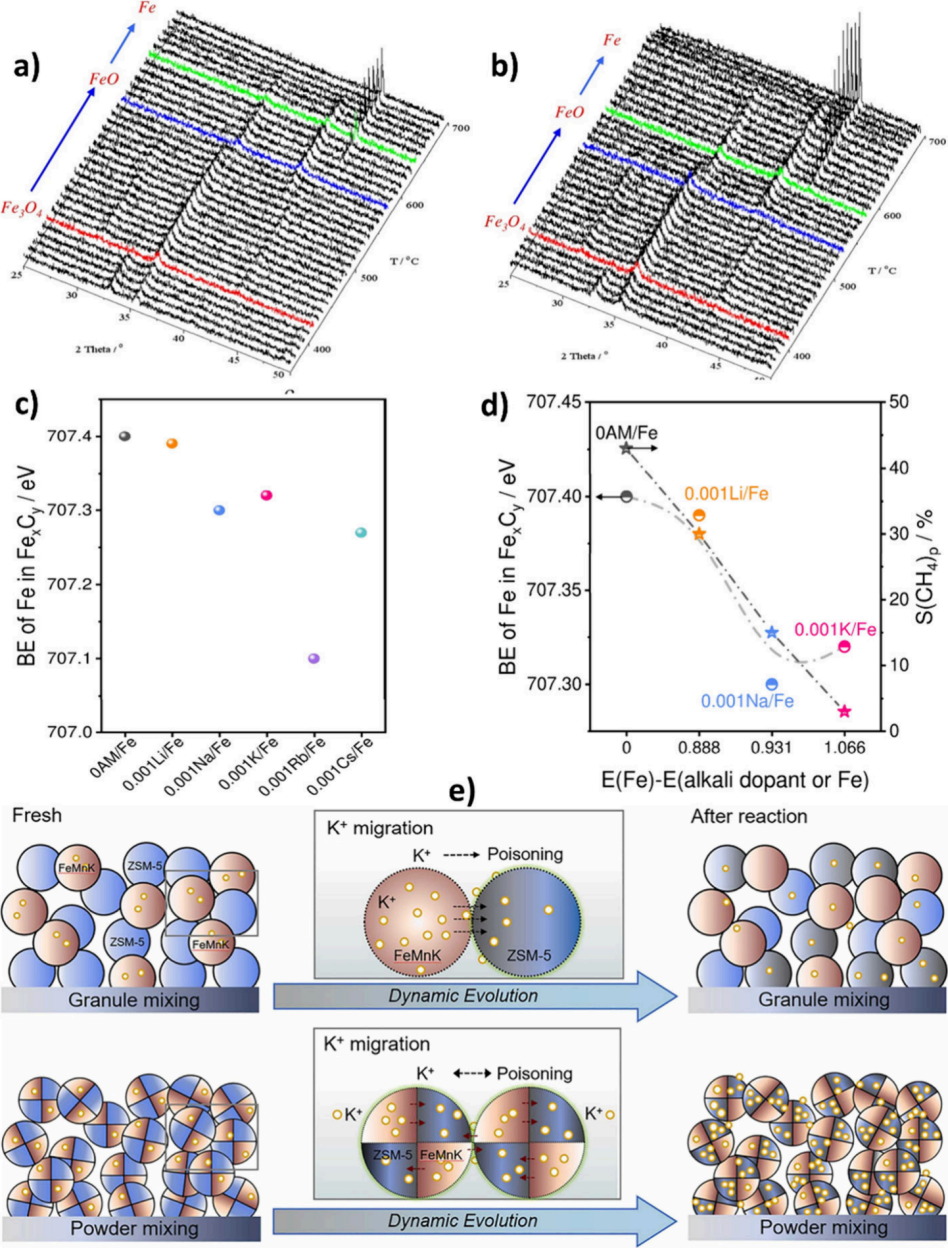
In situ XRD of the hydrogen reduction of **a)** NaFeSi and **b)** KFeSi (Alkali: Fe = 2:100 atomic ratio). Reproduced with permission from ref 92. Copyright permission obtained from Elsevier, 2016. **c)** Binding energy of Fe in Fe-carbide in the presence of alkali metals, and **d)** the dependency of Fe binding energy and CH_4_ selectivity to Allen-scale electronegativity.^104^ Copyright 2022, Wiley-VCH GmbH Publishing under [CC-BY-NC-ND] license. **e)** The influence of the proximity of FeMnK and K^+^ migration to ZSM-5. Reproduced with permission from ref 86. Copyright permission obtained from Elsevier, 2023.

In addition, X-ray photoelectron spectroscopy (XPS) analysis of alkali-promoted Fe/SiO_2_ ^92^ indicated that the concentration of alkalis on the surface is higher than that on the bulk for alkalis with a larger atomic number. Accordingly, Na exhibited a lower surface but a higher bulk concentration, contrary to K, whose surface concentration was larger.^95^ Therefore, Na could interact with more bulk Fe oxide, and bulk oxide species cannot be reduced as easily as surface species, which is likely one of the reasons for the higher inhibitive effect of Na on Fe_2_O_3_ reduction. However, enriching the surface with K forms other Fe species, which can be easily reduced. Additionally, in situ XPS revealed that FeO_*x*_ reduction to metallic Fe was accelerated by K incorporation. It was confirmed that the bond strength of oxidic Fe phases was stronger and more challenging to cleave due to Na-Fe interactions. In addition, oxygen species on the surface can be attracted by bulk Na, which further retard the reduction of iron oxides.^92^ Therefore, it is speculated that forming iron carbides is easier on K-promoted iron oxides.^96–98^

Using Mössbauer spectroscopy, Yang et al.^99^ showed that in the spent Na/Fe-Zn, Fe_3_O_4_ (56.8%) and carbides (43.2%) were present simultaneously, while the spent K/Fe-Zn was mainly comprised of carbides. Also, in the presence of K, more carbonyl and carbon species were formed on the catalyst surface. Based on the scanning transmission electron microscopy (STEM) and electron energy loss spectroscopy (EELS) analysis, in spent Na/Fe-Zn, iron carbides were distributed within 19 nm of the catalyst surface.^99^ Furthermore, density functional theory (DFT) investigations demonstrated that the hydrogenation of surface carbon atoms on different facets of Fe_5_C_2_ depended on the stability of the initial surface species.^52,100^ It was also demonstrated that as the surface, hydrogen, and carbon atoms became more negatively charged in the presence of K_2_O, these species became more stabilized on the surface. In other words, H_2_ dissociative adsorption energy was found to be dependent on charge transfer in the presence of K_2_O.^101^ Accordingly, it can be concluded that since the electronegativity of K is lower than that of Na, K would donate more electron density compared to Na, resulting in more negatively charged surface carbon and hydrogen species. This, in turn, facilitates the carburization and formation of iron carbide.^102,103^ Similarly, Yang et al.^104^ showed that the electronic properties and binding energies of Fe in Fe-carbide can be affected by the presence of alkali metals in alkali metal-promoted Fe_2_O_3_ (Figure 8 (c)). In addition, ethylene (as an intermediate product) adsorption depends on the alkali metal and follows the order Li > Na > K, indicating that the olefin/paraffin ratio is expected to be higher in K-promoted catalysts. The CH_4_ selectivity was found to be correlated with the Allen scale electronegativity of Fe and alkali metals (Figure 8 (d)).^104^

Therefore, the higher carbide formation capability of K provides more active sites for FT reaction and chain growth of CH_2_* to olefins. Meanwhile, easier reduction in the presence of K most likely results in the formation of more carbides thus promoting secondary chain growth reactions and hydrogenation of olefins to heavy nonaromatics rather than aromatics. DFT studies of Yin et al.^105^ showed that the thermal stability of CH_2_ species and the exposure of Fe facets are crucial in tuning CH_4_ selectivity. By stabilizing CH_2_ species and increasing the exposed areas of Fe(211), Fe(310), and particularly Fe(111) surfaces, it is possible to reduce CH_4_ selectivity without compromising activity.^105^ Lee et al.^106^ proposed that alkali metals can augment this difference via electron donation to Fe species in the FT synthesis. Accordingly, Na addition to the 5Fe-SiO_2_ catalyst was found to decrease CH_4_ selectivity from 68.1% (with no Na) to 20.1% (with 30% Na/Fe).^106^

Moreover, in the presence of ZSM-5, both Na and K can migrate from iron oxide to zeolite and cover the surface Brønsted acid sites (BAS).^107^ In other words, the shielding of Brønsted acidity with alkali-promoted iron oxides can affect the hydrocarbon distribution, especially when the promoted Fe-oxide is located in close proximity to the ZSM-5. However, the higher basicity (electron donation) of K compared to that of Na reduces the Brønsted acidity of zeolite to a greater extent. It was revealed that the close proximity of ZSM-5 and FeMnK oxide was detrimental to the catalyst performance due to the migration of K^+^ ions and the mutual poisoning effect of K^+^ and BAS, which neutralize their functions as depicted in Figure 8 (e).^86^ In addition, since Na could retard iron oxide reduction more than K, reduction and carburization in the presence of K would be easier, and therefore, more aliphatic hydrocarbons could be produced. Indeed, these hydrocarbons should undergo aromatization reactions, including cyclization and dehydrogenation, which require a zeolite with a stronger Brønsted acidity.^108^

Figure 9 illustrates the charge transfer and concentration of both Na and K on the surface and bulk of iron oxide while providing a comparison of their roles in reduction and carbide formation. Notably, among the mentioned factors, the type of alkali metal promoter and its loading have a considerable impact on the binding energy of Fe in Fe-carbide^109,110^ and, in turn, the formation of intermediates and final products.

**Figure 9:**
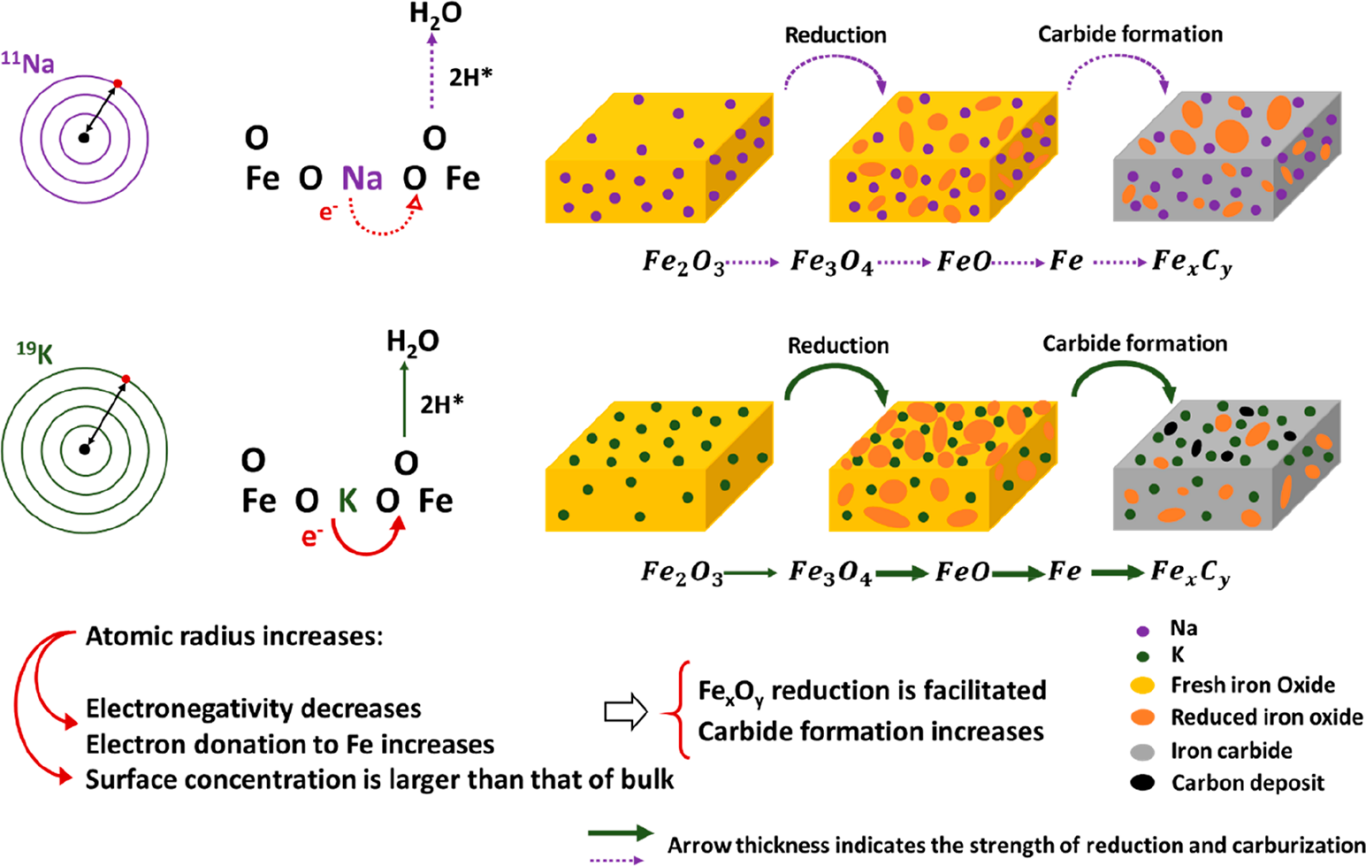
Comparing the roles of Na and K in iron oxide reduction and carbide formation. Arrow thickness has been drawn proportionally, with thicker arrows indicating greater strength (as designed by the authors).

### 4.2 Multivalent Elements

In addition to alkali metals, certain transition metals have been employed as promoters of Fe-based catalysts in CO_2_ hydrogenation toward C_5+_ hydrocarbons.^111,112^ The incorporation of alkali metals and cometals controls the surface composition of active sites involved in the RWGS and FT reactions through essential electronic interactions with Fe-phases. This generates more basic sites for regulating olefin adsorption on the surface.^113,114^ Importantly, the surface adsorption behavior of Fe-based catalysts can be controlled by strategically incorporating suitable ratios of heteroatoms and alkali metals, resulting in a tuned adsorption of CO_2_ and H_2_. This ratio is crucial for favoring olefin formation over paraffins.^115–117^ A large amount of adsorbed hydrogen species can promote further hydrogenation of the produced short-chain olefins, which might adversely affect the overall hydrogenation performance of the Fe-based catalyst.^118,119^ Zn, Mn, Cu, and Co, as transition metal promoters, play a crucial role in enhancing the performance of Fe-based catalysts for CO_2_ hydrogenation to olefins.^120,121^ In this regard, Yang et al.^122^ investigated how Zn, Cu, and Mn enhanced the physicochemical properties and catalytic performance of Fe-based catalysts in CO_2_ hydrogenation. It was found that Zn and Cu modifiers facilitated the reduction and carburization of the iron catalysts, leading to the formation of active Fe_3_O_4_ and Fe_5_C_2_ phases. Additionally, these promoters significantly enhanced the surface basicity and improved the activation of H_2_. On the other hand, introducing Mn improved the reducibility of iron oxides, but the strong interaction between Mn and the Fe species hindered the chemical adsorption of CO and the carburization of metallic Fe in the FeMn-Na catalyst. As a result, the further conversion of CO intermediates was not favored in this case. Consequently, the FeZn-Na catalyst exhibited the highest CO_2_ hydrogenation activity (37.5%) and the lowest CO selectivity (11.5%). However, the presence of Cu species resulted in increased secondary hydrogenation of the formed olefins, leading to a much lower olefin-to-paraffin (O/P) ratio compared to the other investigated catalysts. This effect is primarily attributed to the lower energy barrier of the rate-determining step (RDS) for the secondary hydrogenation reaction at the Cu/Fe_5_C_2_(111) interface, in contrast to the pristine Fe_5_C_2_(111) surface.^122^

#### 4.2.1 Zn

It was found that the presence of Zn atoms could facilitate the reduction of ZnFe_2_O_4_ to Fe_3_O_4_, and further promote the subsequent reduction of Fe_3_O_4_ to FeO and Fe.^123^ Moreover, it was revealed that Zn incorporation could enhance particle dispersion, which provided a better interaction of Zn with both Fe_5_C_2_ and Fe_3_O_4_.^124^ Using in situ XRD, it was revealed that in the FeZn-cp sample, as the temperature increases from 300 to 330 °C, the ZnFe_2_O_4_ phase undergoes reduction, leaving only FeO and ZnO phases. Rapid carburization then converted FeO to Fe_5_C_2_ between 330 and 350 °C. Consequently, ZnO, FeO, and Fe_5_C_2_ coexist in the FeZn-cp catalyst (ZnFe_2_O_4_ prepared by coprecipitation) under both reduction and reaction conditions.^125^ However, during the reduction, ZnFe_2_O_4_ (ZFO) was reduced to ZnO, Fe_3_O_4_, and elemental Fe. Theoretical calculations confirmed that H_2_ preferentially adsorbed on Fe rather than Zn sites. After CO_2_ hydrogenation, the ZFO matrix transformed from ZnFe_2_O_4_ to Fe_3_O_4_, with ZnO as the dominant surface component and Fe_3_C as the key active phase for FTS. Additionally, the presence of Fe–C bonds in the iron carbide phase that formed in ZFO enhanced the RWGS reaction and subsequent carbon chain growth, increasing selectivity for C_2+_ products (Figure 10 (a)).^123^ ZnO has been known as a structural promoter, which enhances the stability and facilitates the dispersion of Fe, which is the active phase for CO_2_ conversion.^125,126^ It has been postulated that CO_2_ can be activated to CO_2_* and subsequently CO* over ZnO, while H_2_ can be converted to H* and then OH* species, forming H_2_O in the next step. The CO* species may diffuse to the Na-Fe_5_C_2_ and undergo FT reaction, including CH_*x*_ formation and C–C chain propagation, to form heavy hydrocarbons, while Na^+^ inhibits further hydrogenation of the intermediates to produce more olefins rather than paraffins.^127^

**Figure 10:**
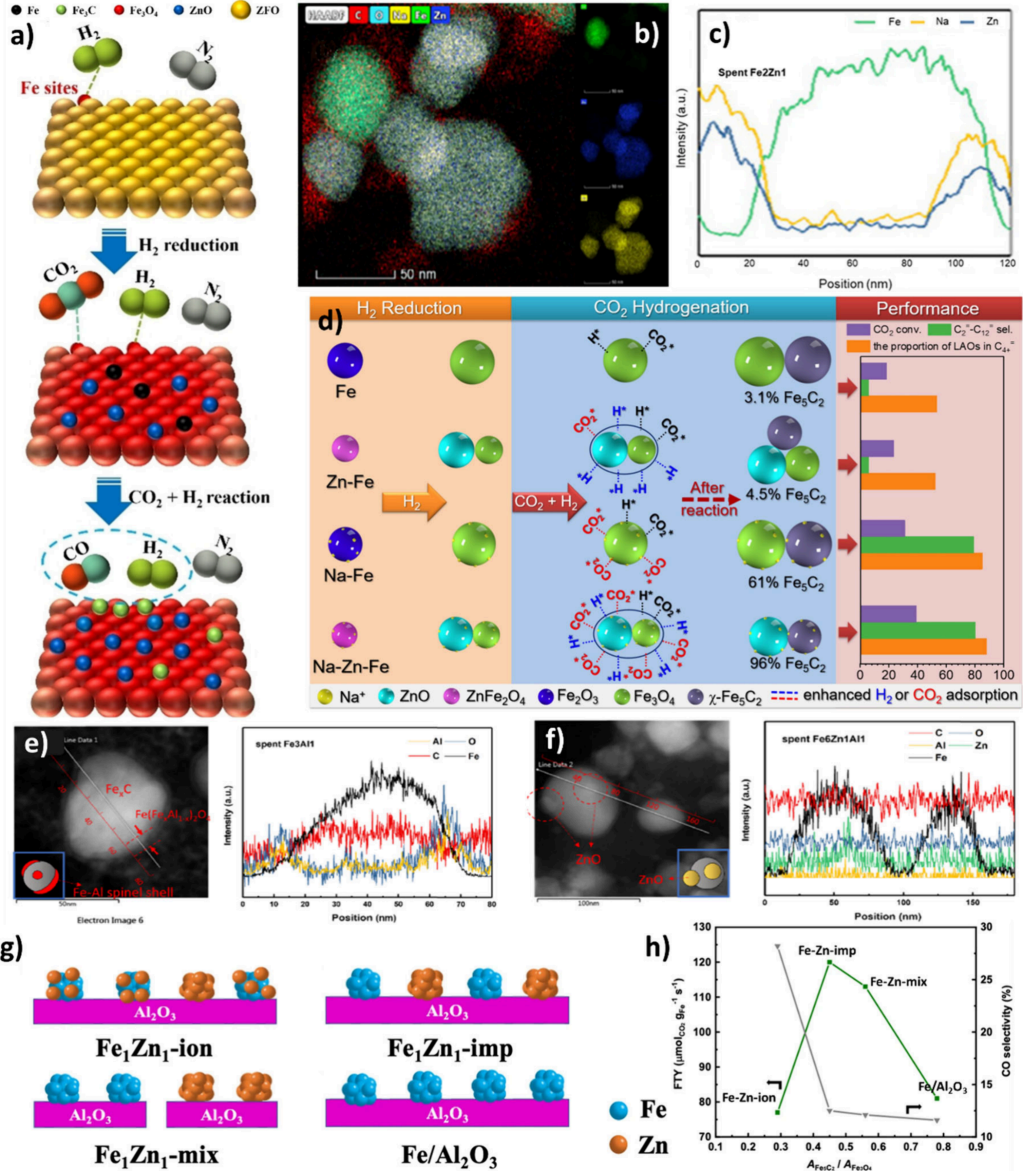
**a)** Structural evolution of ZFO catalyst under reduction and reaction. Reproduced with permission from Cai et al.^123^ Copyright 2023, John Wiley & Sons. **b)** EDX-mapping of the spent Na-Zn1-Fe5 catalyst, **c)** Line scans of the spent Na-Zn1-Fe2 catalyst. Reproduced with permission from Zhang et al.^128^ Copyright permission obtained from ACS, 2021. **d)** A schematic representation of the phase evolution during reduction and carburization, as well as the catalytic performance of Na-Zn-Fe compared with NaFe, ZnFe, and Fe catalysts. Reproduced with permission from Zhang et al.^131^ Copyright 2022, Elsevier. The EDX line scans of **e)** Fe3Al1 and **f)** Fe6Zn1Al1. Reproduced with permission from Xu et al.^135^ Copyright 2021, ACS. **g)** Schematic illustration of different integration manners of Zn and Fe, and **h)** the FTY versus the carbide/oxide ratio. Reproduced with permission from Liu et al.^136^ Copyright 2023, Springer Nature.

To elucidate the synergy between Zn and Na, Zhang et al.^127^ studied the various integration schemes of Na-Fe_5_C_2_ and ZnO and showed that the closest proximity (synthesized via the sol–gel method) enhanced the CO_2_ conversion and decreased CO selectivity. This indicated that the dissociation of CO_2_ to CO and conversion of CO to olefins required a large interface between the active sites to enable CO_2_ adsorption and activation on ZnO and chain propagation on Na-Fe_5_C_2_. In addition, it was shown that Na-Fe_5_C_2_ without Zn could not provide a CO_2_ conversion higher than 3.2% but reached the highest olefin selectivity (80%). However, adding Zn resulted in 38% CO_2_ conversion at almost the same olefin selectivity, confirming the role of ZnO in enhancing CO_2_ conversion. It was suggested that low-valence Zn^δ+^ was oxidized, and ZnO was a sacrificial agent before the Fe_5_C_2_ species. As can be observed in Figure 10 (b), Zn and Na are distributed uniformly in close proximity. The EDX line scans confirm the spatial correlation, since the intensity of the Zn and Na signals coincide in Figure 10 (c).^128^

The incorporation of secondary metals, including Mg^2+^, Co^2+^, Cu^2+^, Fe^2+^, Al^3+^, and Zn^2+^, into trivalent Fe (Fe^3+^) to form lattice Fe oxides with a spinel structure has emerged as a novel approach for deliberate development of highly efficient CO_2_ hydrogenation catalysts.^129,130^ In this framework, Zhang et al.^131^ utilized the electronic properties and surface reconstruction of Na and Zn to create spinel structure nanoparticles. This innovative design facilitated the adsorption of CO_2_ and H_2_ and yielded remarkable results. Accordingly, at 39% CO_2_ conversion, 46% linear α-olefin was produced under industrially relevant conditions. The Na-Zn-Fe catalyst showed a higher presence of Fe_5_C_2_, significantly reducing secondary hydrogenation more effectively than the other catalysts studied, as shown in Figure 10 (d). The study showed that Zn notably decreased the Fe particle size in the catalyst and increased the amount of H_2_ adsorption. In addition, through in situ XPS, the electron transfer from Zn to Fe in spinel ZnFe_2_O_4_ was evident by detecting different chemical states of 0 and +2 for ZnO_*x*_. This electron donation influenced the FeC_*x*_/FeO_*x*_ ratio of the catalyst.^132^ Another study showed that the Na-promoted Fe2Zn1 catalyst, consisting of pure spinel structures, outperformed monometallic Na-Fe_2_O_3_ in producing the C_2_–C_7_ α-olefins. The enhanced stability of the Na-promoted Fe2Zn1 catalyst was credited to the stabilizing influence of Zn on the catalyst surface and the Zn–Na interaction, which suppressed the oxidation of FeC_*x*_ by CO_2_ and H_2_O.^128^ It was found that ensuring high dispersion, precisely controlling the interfacing, and maintaining close proximity of ZnO and Fe_5_C_2_ played a crucial role in effectively synergizing both active centers in the RWGS-FTS reactions.^127^ The enhanced CO_2_ conversion, along with decreased CH_4_ formation, and increased selectivity toward C_2_–C_12_ olefins, was achieved due to the close proximity of the catalyst components.^133^ It was found that the integration of ZnO and Fe_2_O_3_ via physical mixing resulted in a catalyst with inferior performance, especially in olefin selectivity, compared to ZnFe_2_O_4_ synthesized through solvent-thermal methods. This highlights the advantage of a spinel structure for CO_2_ hydrogenation.^134^

However, when Al was introduced, it was demonstrated that Fe_5_C_2_ nanoparticles in the catalyst were enveloped by Fe-Al spinel overlayers through a combination of multiple in situ/ex situ characterizations and DFT calculations. This wrapping process facilitated hydrogenation and hindered C–C coupling over the Fe-Al catalysts. The incorporation of Zn enabled the redistribution of Al, mitigating undesired strong interactions between Fe_5_C_2_ and spinel phases. As a result, the Fe-Al-Zn catalyst exhibited high selectivity toward higher olefins.^135^ In the spent Fe3Al1 catalyst (Figure 10 (e)), the change of the signal intensity of Al and O is synchronized, and several summits are located on the edges or surface areas. At the core of the particle, the intensities of Fe and C reach the peak. This indicates that Fe_*x*_C is partially wrapped with an overlayer of the Fe-Al spinel, and this structure is described as FeAlO_*x*_/Fe_5_C_2_. Unlike Fe3Al1, the Fe_*x*_C particle is not encapsulated by the spinel in the spent Fe6Zn1Al1 (Figure 10 (f)). In addition, no signal of Al is detected in Fe_*x*_C particles, indicating that Fe_*x*_C is separated from the Fe-Zn-Al spinel.^135^ Liu et al.^136^ synthesized a series of alumina-supported Fe-Zn catalysts with different proximities (Figure 10 (g)). It was revealed that decreasing the Fe-Zn distance hampered the reduction of Fe species to metallic Fe. This also hindered further carburization of metallic Fe and promoted the oxidation of carbide during reaction. However, integrating the Fe and Zn at appropriate proximity (Fe-Zn-imp) resulted in the desired Fe_5_C_2_ and Fe_3_O_4_ content and therefore, the FTY (Fe time yield) (Figure 10 (h)).^136^

#### 4.2.2 Cu

Cu plays a crucial role as a promoter in Fe-based catalysts used for CO_2_ hydrogenation. This aids in the reduction of an iron oxide precursor at lower temperatures, thereby enhancing carburization.^137,138^ Its presence in the metallic state during the reaction conditions creates active sites for hydrogen dissociation. Cu, as well as Fe-Cu species, are considered efficient catalysts for the RWGS reaction.^49,139^ It was revealed that adding Cu to Fe-based catalysts can increase CO_2_ adsorption while reducing CO selectivity.^140,141^ In addition, promoting Na-Fe_2_O_3_ with Cu enhanced the C_5+_ formation, indicating that Cu could promote H_2_ dissociation and C–C coupling. Notably, it was found that synergy between Cu and Fe species arose only after the addition of an appropriate amount of Cu to Fe species (6.25% Cu-Fe_2_O_3_), where 56.6% aromatic selectivity could be obtained at 57.3% CO_2_ conversion (Figure 11 (a)).^140^ It was also observed that Cu-promoted catalysts had smaller particles and facilitated the dissociation of intermediates, which hindered byproduct formation. The size of Fe species decreased after adding Cu, which shows that Cu might act as a structural promoter and inhibit Fe-oxide agglomeration.^142^ Choi et al.^143^ reported an impressive C_5+_ hydrocarbon selectivity of 66.3% with only 2.7% CH_4_ using delafossite catalysts (CuFeO_2_-6) for CO_2_ hydrogenation. The catalyst exceptional performance in producing liquid hydrocarbons, compared to Fe_2_O_3_ and CuF_2_O_4_, was ascribed to its rapid reduction behavior and the selective carburization to Fe_5_C_2_. They illustrated that the nature of the Fe–Cu phase plays an essential role in the reduction behavior and carbide formation. It was shown that the ability of CuFeO_2_ to reduce Fe^3+^ to Fe^0^ was much higher than that of CuFe_2_O_4_ spinel. This was attributed to the oxidation states of Cu in different phases, as it is 1^+^ in CuFeO_2_ and 2^+^ in CuFe_2_O_4_, which made the former thermodynamically less stable in the reduction.^143^ Using H_2_-TPR, Liu et al.^139^ and Song et al.^140^ showed that the addition of Cu to Fe-K/Al_2_O_3_ and Fe_2_O_3_, respectively, facilitated the reduction of catalysts at lower temperatures, promoting H_2_ activation compared to the non-Cu-promoted catalyst.^139,140^ The superior catalytic performance of CuFeO_2_ with respect to Fe_2_O_3_, in olefin production, could be attributed to the abundance of surface basic sites and oxygen vacancies.^144,145^ Moreover, it was revealed that the addition of a certain amount of Ga to CuFeO_2_ (0.25Ga-CuFeO_2_) enhanced olefin formation by electron interaction between Ga and Fe, which hindered further hydrogenation of unsaturated hydrocarbons (Figure 11 (b)).^145^ Using XPS, different oxidation states of Cu, Fe, and O on fresh and activated CuFeO_2_ could be observed. Accordingly, Cu^+^ and Fe^3+^ could be observed in fresh catalysts, while Cu^+^, Cu, Fe^3+^, Fe^2+^, and Fe^0^ could be found on the activated catalyst. Besides, more O atoms close to the defect, along with surface hydroxyl groups, were observed on the surface of activated CuFeO_2_. This showed that in the lattice of CuFeO_2_ after activation, Cu^+^ collapsed and aggregated to form a pure Cu phase with a partially oxidized surface. Meanwhile, Fe^3+^ underwent partial reduction and carbonization, resulting in the formation of Fe_3_O_4_, Fe_5_C_2_, and Fe_3_C.^146^ Exploiting DRIFT, SVUV-PIMS, and DFT calculations, Li et al.^146^ showed that the CO insertion mechanism over the interface of Cu/Fe-carbide, along with the carbide mechanism (FT synthesis) on iron carbide, could synergistically enhance C–C coupling and, in turn, C_4+_ formation (around 66.9%) at 27.5% CO_2_ conversion under atmospheric pressure.^146^

**Figure 11:**
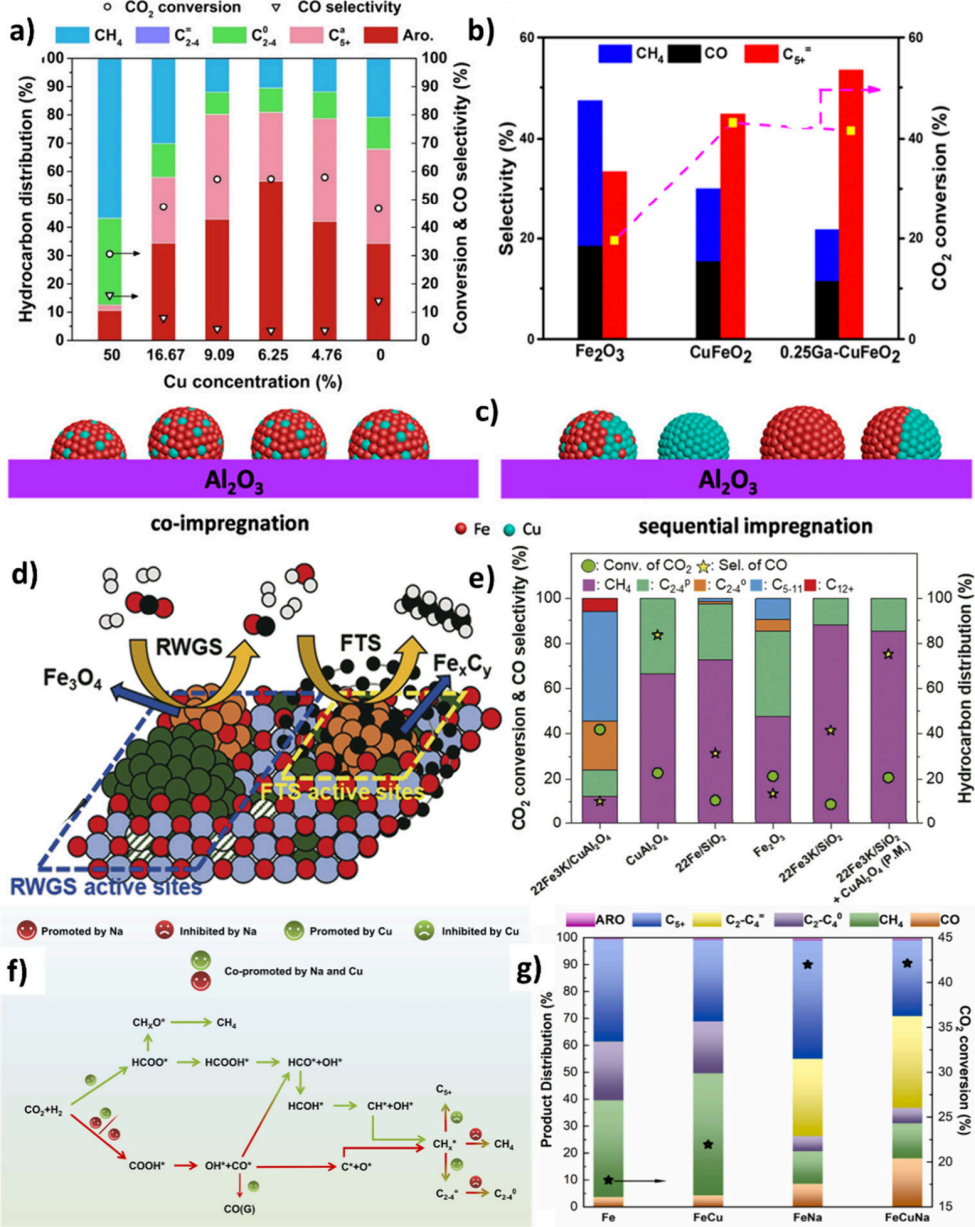
**a)** Effect of Cu concentration on the CO_2_ hydrogenation performance of n–Cu-Fe_2_O_3_/HZSM-5-c. Reproduced with permission from Song et al.^140^ **b)** Catalytic performance of Fe_2_O_3_, CuFeO_2_, and 0.25Ga-CuFeO_2_. Reproduced with permission from Chen et al.^145^ Copyright 2023, RSC. **c)** The effect of the preparation method on the distribution of active sites. Reproduced with permission from Liu et al.^139^ Copyright 2018, ACS. **d)** The active sites of RWGS and FTS in 22Fe3K/CuAl_2_O_4_ and **e)** CO_2_ hydrogenation performance of catalysts (Reaction conditions: 320 °C, 3 MPa, and 10000 mL g_cat_^–1^ h^–1^). Reproduced with permission from Kim et al.^147^ Copyright 2022, RSC. **f)** The schematic illustration of Na and Cu roles in Fe-based catalysts. **g)** The CO_2_ hydrogenation performance of Na and Cu promoted Fe-based catalyst (Reaction conditions: 230 °C, 3 MPa, and 1500 mL g_cat_^–1^ h^–1^). Reproduced with permission from Chen et al.^148^ Copyright 2024, Elsevier.

It is believed that for Cu to function at its best, it needs to be located in close proximity to Fe species. To verify this claim, Liu et al.^139^ utilized coimpregnation and sequential impregnation techniques to regulate the arrangement of Cu and Fe in close proximity to each other. The catalysts produced through sequential impregnation have lower BET-specific surface areas compared to those produced through coimpregnation. The processes of nitrogen adsorption and desorption suggest that the structure of the catalysts was influenced by the method of preparation. In contrast to the coimpregnated catalyst, diffraction peaks of hematite phases were strong in sequentially impregnated ones, suggesting that using Fe and Cu separately led to the relatively complete development of hematite phases. Clearly, α-Fe_2_O_3_ was the predominant form of Fe present, and notably, CuO particles were closely linked to Fe oxide particles in the sequentially impregnated catalysts (Figure 11 (c)).

Kim et al.^147^ also showed that a strong interaction between Fe_3_O_4_, Cu, and CuAl_2_O_4_ resulted in a synergistic effect that led to the effective coupling of RWGS and FTS reactions, as depicted in Figure 11 (d). The inferior performance of physically mixed 22Fe3K/SiO_2_ and CuAl_2_O_4_ (20.7% CO_2_ conversion and no C_5+_ formation) compared to 22Fe3K/CuAl_2_O_4_ (41.9% CO_2_ conversion and 48.9% C_5+_ selectivity), Figure 11 (e), revealed that atomic-scale interactions and their close proximity played a significant role in coupling RWGS and FTS reactions.^147^

The addition of Cu and K as promoters influences the rate of Fe reduction and carburization. Cu significantly enhances both the reduction of hematite and the carburization of magnetite, whereas K hinders the reduction of hematite to magnetite. Cu-promoted catalysts exhibited much higher reaction rates than unpromoted and K-promoted catalysts, while K-promotion had a more pronounced effect on selectivity.^149^ The observed increase in catalytic activity is mainly attributed to the higher carbide content and larger carbide surface area in the promoted catalysts.^150,151^ Using DFT calculations and different characterizations, Chen et al.^148^ showed that, in Na-Cu-promoted Fe, Na enhanced CO_2_ adsorption and iron carbide formation, while Cu promoted H-spillover on Na-doped catalysts, resulting in the formation of oxygen vacancies for the adsorption and activation of CO_2_. In addition, Cu promoted nondissociative activation of CO on Na-promoted Fe, which hindered the formation of the CHx*-rich surface. These effects led to the formation of more C_2_–C_4_ (39.81%) and a restriction of C–C coupling, as depicted in Figures 11 (f) and (g).^148^

#### 4.2.3 Mn

Mn is recognized as an effective transition metal promoter in CO_2_ hydrogenation for producing α-olefins.^152,153^ The Mn promoter offers excellent electronic tuning capabilities, making it suitable for catalyst modification. Due to the polyvalency of Mn, Mn^3+^ can replace Fe^3+^ in Fe_2_O_3_, forming a solid solution. Moreover, Mn^2+^ can substitute Fe^2+^ in Fe_3_O_4_, creating a mixed MnFe_2_O_4_ spinel phase. However, due to the variable oxidation state of Mn, Mn-Fe spinel catalysts are prone to oxidation and may experience phase segregation, in contrast to Zn-Fe counterparts.^129^ To unravel how Na and Mn influence the performance of CO_2_ hydrogenation, unpromoted and promoted Fe_2_O_3_ catalysts were synthesized and tested for CO_2_ conversion (Figure 12 (a)). The pure Fe_2_O_3_ catalyst exhibited the highest production of methane and light paraffins, along with the highest CO selectivity at the lowest CO_2_ conversion. With the introduction of Mn, the aromatics selectivity improved. However, methane and light paraffins still constituted a significant portion of the hydrocarbon products, and CO selectivity remained high. On the contrary, Na-promoted Fe-based catalysts demonstrated more favorable CO_2_ hydrogenation performance, resulting in a significantly improved distribution of liquid hydrocarbons, including both aromatics and aliphatic compounds. Interestingly, over NaFeMn/HZSM-5 (with a Fe/Mn ratio of 5/1), a relatively lower selectivity of CH_4_ (7%) and CO (10%) was observed at higher CO_2_ conversion (42%) compared to NaFe/HZSM-5.^154^

**Figure 12:**
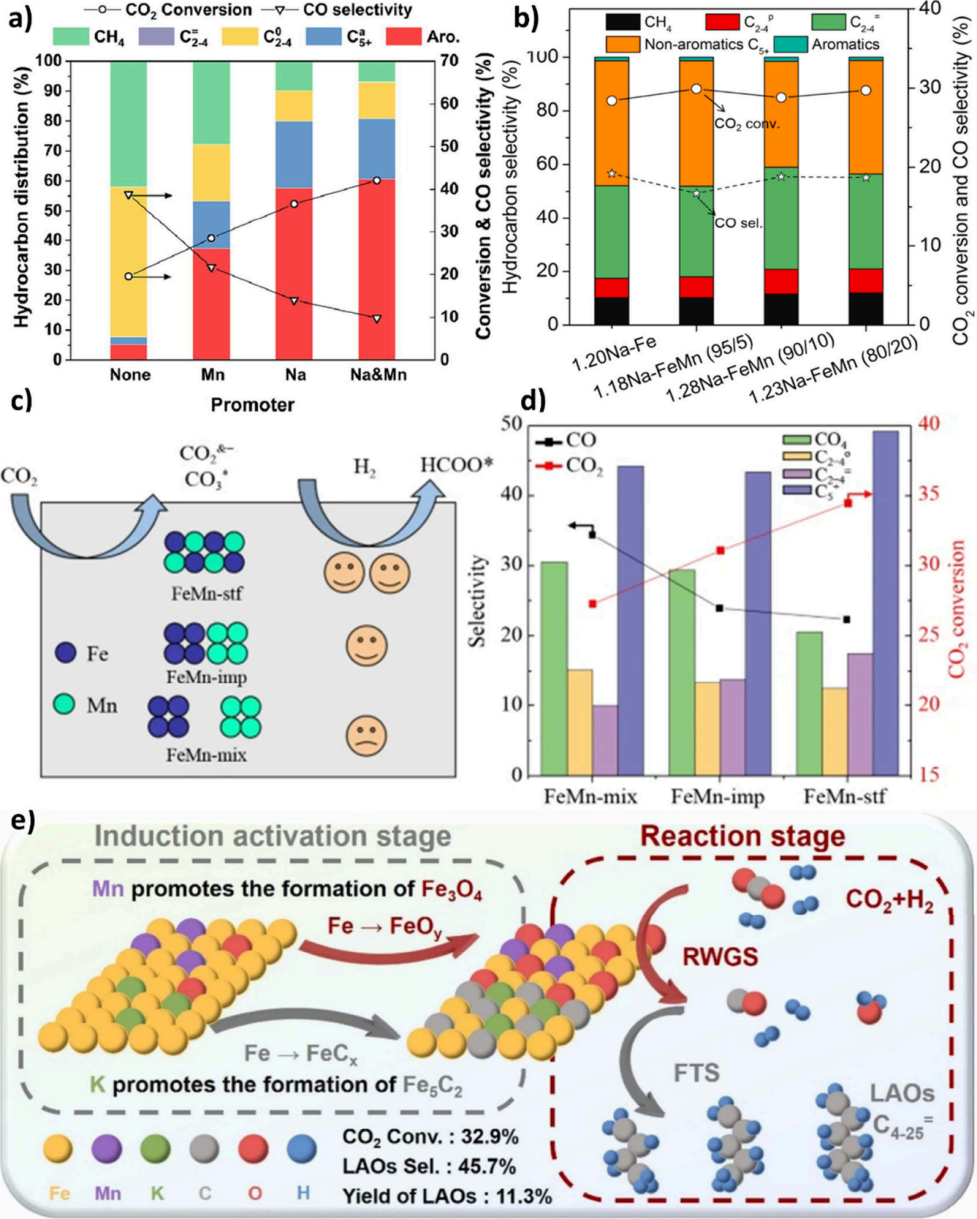
**a)** The effect of Na and/or Mn promotion on Fe_2_O_3_ performance in the product distribution of CO_2_ hydrogenation. Reproduced with permission from Song et al.^154^ Copyright 2023, Elsevier. **b)** CO_2_ hydrogenation performance of Fe-Mn catalysts prepared via different integration methods. Reproduced with permission from Gao et al.^156^ Copyright 2022, Elsevier. **c)** Schematic illustration of varying integration manners of Fe and Mn and **d)** CO_2_ hydrogenation performance at different Fe-Mn integration manners. Reproduced with permission from Liang et al.^165^ Copyright 2024, Springer. **e)** Schematic illustration of Fe-Mn-K catalyst in CO_2_ hydrogenation reaction. Reproduced with permission from Ren et al.^166^ Copyright 2024, Elsevier.

Surprisingly, introducing Na as the third component has reduced the strong interaction between Mn and Fe, improving CO_2_ hydrogenation and selectivity toward alpha olefins. Accordingly, 60% C_4_–C_20_ olefins were produced at 35% CO_2_ conversion in the presence of the Fe-Mn-Na catalyst. Furthermore, the synergy between Na and Mn depended on their ratio, and the enhancement in CO_2_ hydrogenation could not be achieved by adding these two promoters separately. At 0.6 wt % Na content, as the Mn/Fe molar ratio increased from 1:3 to 1:1, CO selectivity rose from 17.5% to 27.5%, while C_2+_ hydrocarbon selectivity dropped from 71.2% to 61.0%. This indicates that at higher Mn/Na ratios, the FTS performance is reduced.^117^ Singh et al.^155^ also demonstrated that the optimal addition of Mn resulted in higher catalyst reducibility. The DFT calculations indicated a reduction in the activation energy barrier for oxygen vacancy generation on the Na-Mn_4_O_4_-CuFeO_2_ (1 0 2) surface, suggesting the promoting effect of MnO on the increased reducibility of the MnO/Na-CuFeO_2_ catalyst. Additionally, the kinetic studies showed that the presence of Mn in the catalyst facilitated the direct hydrogenation of CO_2_ into hydrocarbons by lowering the activation energy and increasing the reaction rate.^155^ Furthermore, it was found that the selectivity to C_2_–C_4_ olefins continuously increased, reaching a maximum value of about 40.3% when the Mn loading reached an Mn/Fe ratio of 0.14. However, further increasing the Mn loading did not lead to additional enhancement in the selectivity of C_2_–C_4_ olefins. Instead, the selectivity to CH_4_ and C_2_–C_4_ paraffins slightly increased, while there was a significant decline in the selectivity to C_5+_ hydrocarbons, decreasing from 54.2% to 37.9%. Notably, the CO_2_ conversion remained unaffected by the Mn loading amount, but the selectivity to CO increased with higher Mn loading.^96^ CO_2_-TPD results showed that the addition of Mn to FeMn (95/5) increased CO_2_ adsorption, whereas further incorporation of Mn into FeMn (80/20) decreased CO_2_ uptake, which may be due to the covering of Fe species with Mn^156^ (Figure 12 (b)). A similar phenomenon was reported by Song et al.,^157^ due to similar atomic radii, whereby Mn could cover Fe sites when Fe_2_O_3_ powder was impregnated by both Na and Mn.

Recently, Yang et al.^158^ identified Fe-Mn-K as a high-performance catalyst via statistical analysis of existing literature data. This catalyst exhibited a 30.4% light olefin selectivity at 42.3% CO_2_ conversion. In this regard, Zhang et al.^159^ demonstrated that adding Mn and K improved the C–C coupling reaction, but their effects differed. Mn promoted the formation of C_2_–C_4_ olefins, while K favored the production of long-chain hydrocarbons. The presence of Mn not only adjusted the FeO_*x*_/FeC_*x*_ ratio but also facilitated electron transfer to the Fe_3_C surface, optimizing the C/H ratio on the active site. This resulted in Fe_3_C nanoparticles with a high electron density, which hindered the hydrogenation of unsaturated CH_*x*_ intermediate species and olefins, thereby increasing the selectivity for light olefins. In addition, Mn improved the stability of Fe_3_C by preventing the overflow of C atoms from Fe_3_C nanoparticles, which was attributed to the interaction between Mn and C.^159^ Hence, adding Mn and K significantly impacted the product distribution during CO_2_ hydrogenation over the iron carbide catalyst.^160,161^ Furthermore, it was found that Fe-Mn-K (with a molar ratio of 10:1:1) synthesized with the organic combustion method exhibited superior CO_2_ conversion rates and higher selectivity to jet fuel range hydrocarbons (47.8% selectivity) compared to nonpromoted catalysts.^162^ This suggests that the synthesis method can have a significant impact on the performance of a catalyst. Interestingly, it was discovered that Mn could hinder Na migration to the zeolite, thus maintaining zeolite acidity for aromatization. The migration of Mn on zeolite from NaFeMn could promote the formation of CO_2_-oxidizable coke, leading to accelerated coke oxidation by CO_2_.^163^ However, Gao et al.^156^ showed that incorporating Mn into Na-Fe catalysts augmented the amount of light hydrocarbons. It was also revealed that the chain growth reaction was hindered via Mn promotion (for Fe/Mn = 90/10), which resulted in higher light olefin selectivity (38.2%) with respect to that of Na-Fe (34.7%). The same phenomenon was reported by Liang et al.,^164^ where introducing an appropriate amount of Mn (5 wt%) to the Na/Fe catalyst promoted the development of an active Fe_5_C_2_ phase, resulting in a 30.2% selectivity of light olefins. This is another indication of the importance of the synthesis method and promoter incorporation into the Fe oxide-based catalysts. Liang et al.^165^ prepared Fe-Mn catalysts via three integration methods, i.e., spinel type ferrite (stf), coimpregnation (imp), and powder mixing (mix) (Figure 12 (c)), and using XPS showed that shortening the Fe-Mn distance via the stf method enhanced electron donation to Fe and favored the formation of more HCOO*. In addition, it was revealed that the FeMn-stf catalyst exhibited higher CO_2_ adsorption capacity and C_5+_ selectivity (Figure 12 (d)).^165^

Exploiting DFT calculations and in situ XPS, Liu et al.^96^ showed that Na facilitated the reduction via electron donation, while Mn could favor the reduction by promoting the oxygen in Fe-oxide to spillover to oxygen vacancies in the MnO. Indeed, MnO_2_ can undergo reduction to MnO at a lower temperature than the reduction of Fe_3_O_4_ to FeO. The reduced MnO, along with the presence of oxygen vacancies, may play a crucial role in weakening the Fe–O bond and promoting the removal of oxygen atoms in Fe oxides. Additionally, using H_2_-TPR, it was indicated that Mn addition could decrease the reduction temperature. Ren et al.^166^ demonstrated that Mn incorporation can enhance the dispersion of Fe species, thereby promoting Fe_3_O_4_ formation, which is active for RWGS. Note that by tuning Mn and K content, the Fe_3_O_4_ and Fe_5_C_2_ amounts could be adjusted for coupling RWGS and FT reactions (Figure 12 (e)).^166^

#### 4.2.4 Co

To improve CO_2_ conversion and adjust the product distribution toward C_5+_ hydrocarbons, small amounts of Co can be added to Fe-based catalysts.^167,168^ It has been suggested that effective dispersion and close proximity between Fe and Co sites facilitate the inhibition of methane formation and promote higher selectivity toward C_2+_ hydrocarbons, particularly lower olefins.^169^ Generally, if the Co-Fe bimetallic catalyst has a well-defined crystalline nanostructure, the structural and electronic properties of the catalyst can be further tailored to improve the catalytic activity and selectivity. The H_2_-TPR and in situ XRD experiments demonstrated that adding Co elements could enhance the reducibility of catalysts. It was revealed that the Co_1_Fe_2_ catalyst, with a pure spinel structure of CoFe_2_O_4_ in the precursor, showed a significantly higher content of (Fe_1–*x*_Co_*x*_)_5_C_2_ alloy carbide. However, precursors with a Co/Fe molar ratio greater than 1/2 tended to form the Co_2_C structure instead of bimetallic alloy carbide, leading to poor performance in olefin synthesis.^170^ Therefore, the proximity of Co and Fe elements in Co-Fe bimetallic catalysts significantly influences their structural evolution during reduction and reaction processes, as illustrated in Figure 13 (a).^170^ The im-Co1Fe2 catalyst, created by impregnating Fe_2_O_3_ with Co^3+^ solution, and the phy-Co1Fe2 catalyst, made by physically mixing of Fe_2_O_3_ and Co_3_O_4_, both exhibit weaker interactions between Co and Fe compared to the Co1Fe2 catalyst. Consequently, the alloying extent of the Co_*x*_Fe_*y*_ and (Co_*x*_Fe_1–*x*_)_5_C_2_ carbide phases during the reduction and reaction processes differs from that of the Co1Fe2 catalyst, leading to poorer performance in olefin synthesis.^170^ It was also confirmed by Kim et al.^171^ that the single-source precursor Na-CoFe_2_O_4_ formed only a single-phase bimetallic alloy carbide, i.e., (Fe_1–*x*_Co_*x*_)_5_C_2_. In contrast, the mixed precursor (Na-Fe_3_O_4_ + Co) resulted in an isolated Co phase as well, which promotes the undesirable formation of CH_4_ (Figure 13 (b)).^171^

**Figure 13:**
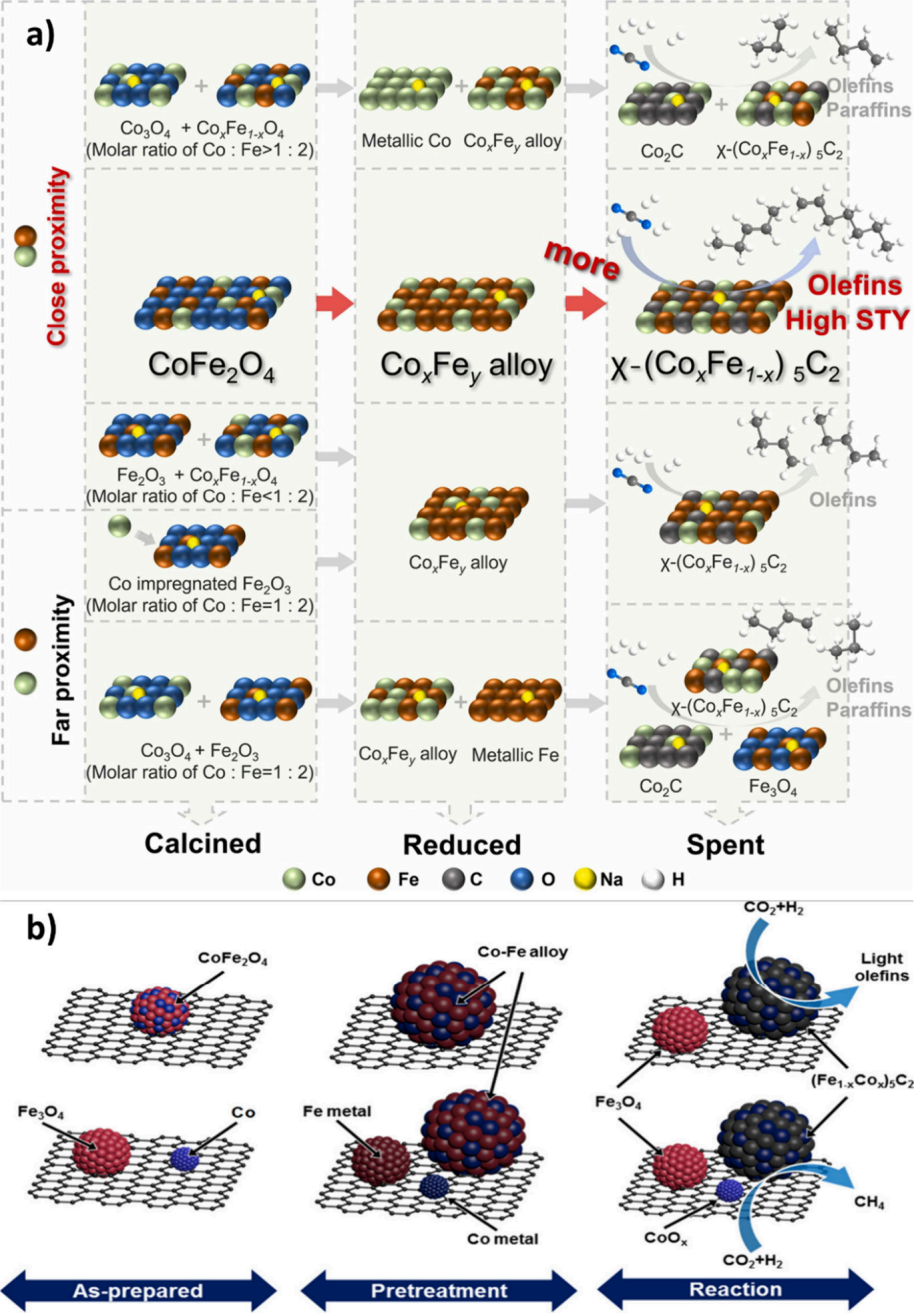
**a)** Phase evolution of Co-Fe bimetallic catalyst. Reproduced with permission from Liu et al.^170^ Copyright 2023, Elsevier. **b)** Schematic illustrations of the phase evolution of CNT-supported CoFe_2_O_4_ (Top) and (Fe_3_O_4_+Co) (Bottom) during the reduction and reaction. Reproduced with permission from Kim et al.^171^ Copyright 2020, ACS.

The effects of the proximity between Co and Fe sites at different proximities have been further studied by Jiang et al.^172^ The findings indicated that closer contact between Fe and Co sites enhanced the selectivity for C_2+_ hydrocarbons. This has been attributed to the efficient transfer of CO intermediates from Fe_3_O_4_ to Co active sites, resulting in a higher CO concentration at the Co sites. Conversely, as the distance between Fe and Co sites increased, the selectivity for CH_4_ rose significantly, which was attributed to the promotion of direct CO_2_ methanation. Also, the likelihood of chain growth in the FTS reaction decreased due to the reduced CO concentration at the Co sites.^172^

Zhang et al.^113^ developed a Na-modified CoFe alloy catalyst using layered double-hydroxide (LDH) precursors that directly converted CO_2_ into jet fuel. The catalyst achieved an exceptionally high C_8_–C_16_ selectivity of 63.5%, with a CO_2_ conversion of 10.2%. It was demonstrated that the Na-modified CoFe alloy phase exhibited intermediate chain propagation activity, which enhanced the C–C coupling reaction, leading to a high selectivity for C_8_–C_16_ hydrocarbons. Furthermore, the incorporation of Na and the formation of the CoFe alloy structure can effectively inhibit CO_2_ methanation.^113^ Yuan et al.^173^ also employed LDH as a precursor to synthesize Fe-Co bimetallic catalysts in close proximity. It was demonstrated that adding 10% Co (FeCo-9:1-LDH) significantly boosted the light olefin selectivity to 36.4%. The olefin-to-paraffin ratio on this catalyst reached 3.5, the highest among all FeCo-x:y-LDH catalysts.^173^ Xu et al.^174^ ascribed the inhibited CO_2_ methanation over ZnCoxFe_2–*x*_O_4_ catalysts to the formation of Fe-Co carbide, Co_2_C, and θ-Fe_3_C phases during the CO_2_ hydrogenation. Guo et al.^175^ demonstrated that Co promoted the reduction of Fe-oxide and enhanced CO_2_ adsorption to iron species in K-ZnFe-Co catalysts. DRIFT analysis revealed that, in addition to the carbide mechanism, the presence of Co_3_Fe_7_ provided O-containing species for chain propagation via oxygenate pathway Figure 14 (a), (b). The catalyst showed 50.2% CO_2_ conversion and 8.1% selectivity to CO, while achieving a C_5+_ yield of 26.7% when Co/Fe= 5.^175^

**Figure 14:**
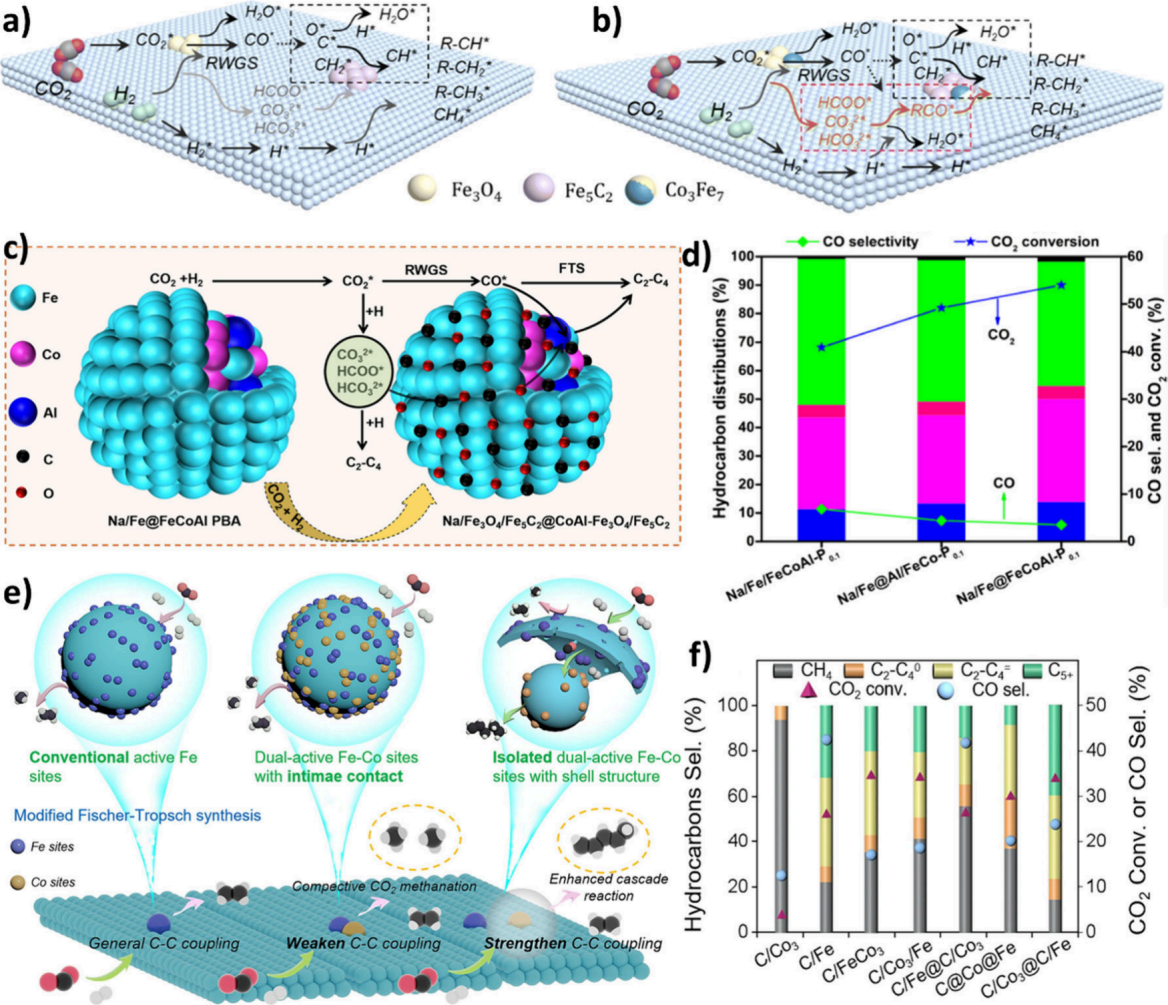
Schematic illustration of reaction pathway over: **a)** KZnFe, and **b)** KZnFe-5.0Co catalysts for hydrocarbons formation via CO_2_ hydrogenation. Reproduced with permission from Guo et al.^175^ Copyright 2023, the Royal Society Publishing under [CC BY-NC 3.0] license. **c)** Schematic demonstration of reaction mechanism over the Na/Fe@FeCoAl PBA catalyst. **d)** CO_2_ hydrogenation performance of the catalysts integrated in different manners (Reaction conditions: 330 °C, 3.0 MPa, and 4800 mL g^–1^ h^–1^). Reproduced with permission from Li et al.^176^ Copyright 2023, ACS. **e)** Schematic representation of reaction path over C/Fe, C/FeCo_3_, and C/Co_3_@C/Fe catalysts, **f)** CO_2_ hydrogenation performances over different catalysts. Reproduced with permission from Wang et al.^177^ Copyright 2024, Elsevier.

Li et al.^176^ introduced the Na/Fe@FeCoAl-P core–shell catalyst with distinct interfaces between Fe and FeCoAl PBA, which exhibited 36.2% selectivity to light olefins at a 54% CO_2_ conversion. This can be ascribed to dual-active interfaces, where CO_2_ could be activated on Fe in the shell, while C–C coupling proceeded on Co in the core (Figure 14 (c)). However, other catalysts, such as Na/Fe/FeCoAl-P0.1 and Na/Fe@Al/FeCo-P0.1, exhibited lower CO_2_ hydrogenation performance (Figure 14 (d)), indicating the role of proximity and spatial arrangement of the active sites in catalytic performance. It is noteworthy that FeCoAl PBA facilitated the Fe_5_C_2_ formation while hindering the formation of heavy hydrocarbons due to the specific porous structure.^176^ In another study, Wang et al.^177^ developed isolated dual-active Fe–Co catalysts (C/Co_3_@C/Fe) via a multistep impregnation and hydrothermal synthesis method, with Co in the core and Fe in the shell (Figure 14 (e)). The 3D structure of the catalyst provided a CO-rich environment favorable for C–C coupling and enhanced C_5+_ formation (19.8% to 39.7%), while CH_4_ formation reduced considerably (from 35.5% to 14.4%) compared to C/FeCo_3_ (Figure 14 (f)).^177^

## 5 Tuning Zeolite Properties

Zeolite is a type of crystalline microporous material with the distinctive characteristic of having well-defined channels/pores and cages, which enable the adsorption, diffusion, transformation, and reaction of molecules. These channels and cages are constructed by connecting TO_4_ tetrahedra (where T can be Si, Al, P, and other elements) that share corners. The copresence of AlO_4_ and SiO_4_ units results in a negative charge on the zeolite framework. To maintain electroneutrality, a balancing cation (typically H^+^) is required, leading to the formation of Brønsted acid sites (BAS), which is one of the significant features of zeolites.^178,179^ By regulating synthesis conditions, various metals can be incorporated into the zeolite framework, leading to the development of heteroatomic zeolites that possess Lewis acidity.^180^ Zeolites are widely used in catalysis due to their shape-selective capabilities, uniform pores, controllable acidities, and excellent thermal and hydrothermal stability.^181–183^ The topologies of the popular zeolites utilized in CO and CO_2_ hydrogenation to form hydrocarbons are illustrated in Figure 15 (a). The use of multifunctional catalysts that combine the properties of zeolites and active metal has enhanced the potential of zeolites for catalyzing diverse chemical processes. The properties of zeolites have been found to influence the reaction products generated on metal catalysts, serving as intermediates for secondary reaction.^183,184^ In addition, zeolites can anchor certain metallic species, leading to improved selectivity for desired products and enhanced antisintering abilities during CO_2_ conversion.^185–187^ Figure 15 (b) illustrates the BAS of a typical zeolite crystal and the most relevant phenomena, including adsorption, diffusion, and reaction.^188^

**Figure 15:**
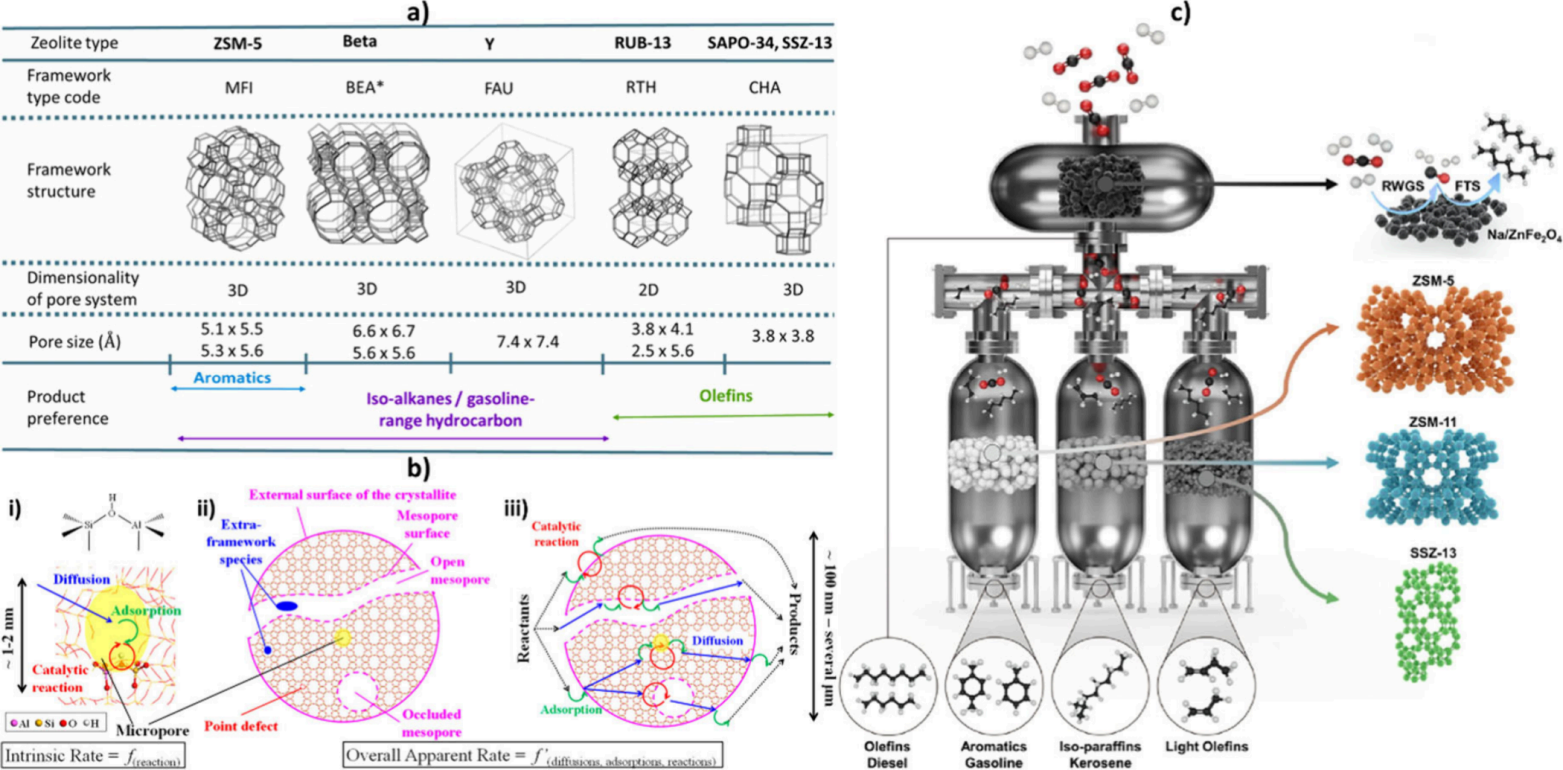
**a)** Schematic of common zeolite topologies (cage/channel dimensions) and corresponding hydrocarbon products. Reproduced with permission from Azhari et al.^183^ Copyright 2022, Elsevier. **b) i)** Model of a BAS zeolite confined to the micropores, considering the diffusion, adsorption, and reaction steps, **ii)** Diagram showing the inherent and finite size properties of a complex zeolite crystallite, modeled as a spherical crystallite for simplicity, and **iii)** The combination of different sites and steps is used to determine the overall observed reaction rate. Reproduced with permission from Chizallet et al.^188^ Copyright 2023, ACS. **c)** Selective hydrocarbon production via combining the catalyst system of Na/ZnFe_2_O_4_ and an appropriate zeolite. Reproduced with permission from Ra et al.^189^ Copyright 2023, Elsevier.

By carefully selecting a suitable zeolite based on factors such as pore size, channel shape, and acid strength, it is possible to design catalysts that selectively drive reactions to a desired product distribution. In Figure 15 (c), a hybrid catalyst system is designed for the selective production of hydrocarbons from CO_2_ hydrogenation. CO_2_ enters the system and undergoes RWGS and FTS reactions facilitated by the Na/ZnFe_2_O_4_ catalyst. The resulting intermediates (olefins and diesel) pass through different reactors containing ZSM-5, ZSM-11, and SSZ-13 zeolites. Each zeolite type selectively converts the intermediates into specific hydrocarbons: ZSM-5 produces aromatics and gasoline, ZSM-11 produces iso-paraffins and kerosene, and SSZ-13 produces light olefins. This integrated approach, utilizing distinct zeolite topologies, optimizes the conversion of CO_2_ into a variety of hydrocarbons, demonstrating an efficient process for sustainable fuel production.^189^

### 5.1 Topology

The micropore structure and morphology of the zeolite primarily determine the selective production of hydrocarbons from the CO_2_ hydrogenation reaction.^190,191^ The optimal pore diameter for the formation of aromatics typically falls within the range of 5–6 Å. ZSM-5, which features a three-dimensional, 10-membered-ring channel pore system (Figure 16 (a)),^192^ is the most desirable zeolite framework for synthesizing aromatics (Figure 16 (b)) due to its outstanding ability to promote aromatization as well as its resistance to coke-induced deactivation.^193–195^ The diffusion limitation is one of the most critical challenges for the zeolite-based bifunctional catalysts. The intermediates formed over Fe-based oxide can diffuse into the micropores of zeolites, where the isomerization and hydrocracking occur on the acid sites. However, the slow diffusion of hydrocarbons through the long micropores could result in overcracking and alter the product distribution to light hydrocarbons.^196^ Nonetheless, it has been revealed that controlled mass transfer limitations within zeolite pores/cages can be used as an effective tool to steer the mechanism and, thus, the product distribution toward the desired direction.^197^ The aromatics formed via light olefin-based intermediates can diffuse out through both straight and sinusoidal channels. Wen et al.^198^ showed that by lengthening the *b*-axis (straight channel) of chainlike ZSM-5 (CLZ5), the diffusion of products can be restricted, and aromatics can only diffuse out through the sinusoidal channels, as depicted in Figure 16 (c). This hinders heavy aromatic formation in straight channels and increases the selectivity toward light aromatics, especially toluene, in products.^198^ In addition, Liu et al.^199^ showed that aromatics formation could be facilitated by increasing both particle size and hierarchy, and, in turn, residence time in ZSM-5 channels. Accordingly, the microporous HZSM-5 (m-Z5) and hierarchical HZSM-5 (h-Z5) exhibited the shortest and the longest diffusion paths, which showed the lowest and the highest aromatics and especially benzene, toluene, and xylenes (BTX) selectivity (Figure 16 (d), (e)),^199^ indicating the role of mass transfer limitations in altering the hydrocarbon selectivity. Gu et al.^200^ showed that by changing the location of Al and, in turn, the BAS of the ZSM-5, the distribution of products can be altered. It was indicated that although the presence of BAS at the intersection of sinusoidal and straight channels could facilitate the formation of aromatics, the BAS at the channels reduced aromatic selectivity due to more constraining effects. Moreover, external BAS could promote hydrocracking and isomerization of the aromatics that diffused out of the channels, hence altering the distribution toward more iso-paraffins rather than aromatics, as observed on Na-Fe@C/Z-5-Hexagon and Na-Fe@C/Z-5-Sheet.^200^

**Figure 16:**
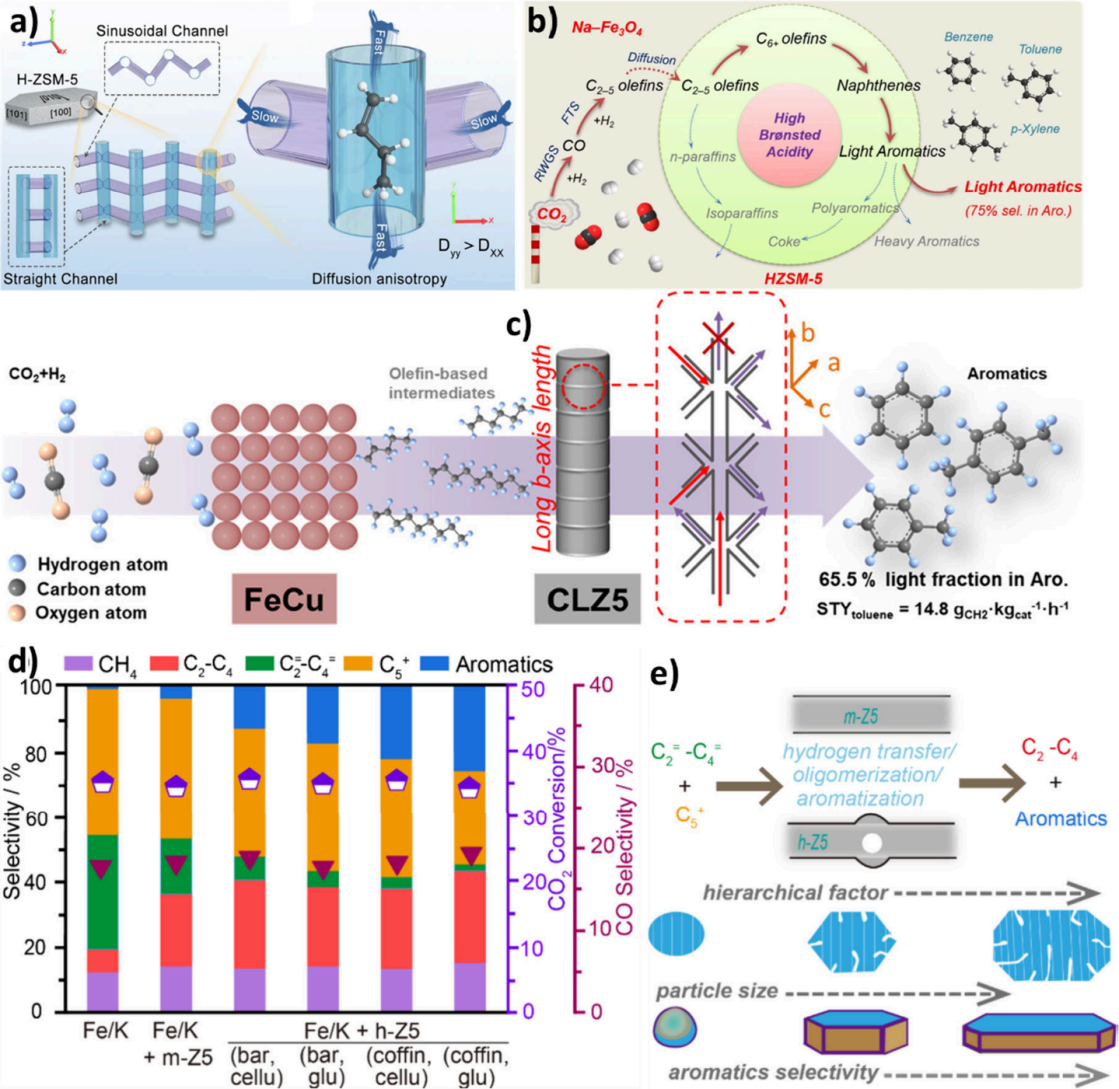
**a)** Schematic view of the channels (straight and sinusoidal) with different geometry shapes but similar opening sizes, and diffusion anisotropy in a two-channel network of ZSM-5. Reproduced with permission from Liu et al.^192^ Copyright 2021, Nature Springer Publishing under [CC BY 4.0] license. **b)** Schematic illustration of aromatics formation over ZSM-5. Reproduced with permission from Wei et al.,^194^ Copyright 2021, Elsevier. **c)** Enhanced light aromatics formation via *b*-axis lengthening. Reproduced with permission from Wen et al.^198^ Copyright 2023, ACS. **d)** Hydrocarbon distribution over Fe_2_O_3_@KO_2_/ZSM-5. **e)** A schematic illustration of the impact of particle size and hierarchy on aromatics selectivity. Reproduced with permission from Liu et al.^199^ Copyright 2023, Wiley.

To further investigate the influence of zeolite topology on product distribution in CO_2_ hydrogenation, Wei et al.^201^ used various types of zeolites with different channel systems (such as HY, HMOR, HBEA, HZSM-5, HZSM-23, and HMCM-22) in combination with Na-Fe_3_O_4_. Although the type of zeolite used did not have a significant impact on CO_2_ conversion and CO selectivity (Figure 17 (a)), it had a noticeable influence on the distribution of produced hydrocarbons (Figure 17 (b)), which was related to the type of channels present in the zeolite. Zeolites with 10-member ring channels are more likely to steer the selectivity toward C_5_–C_11_ hydrocarbons. As the dimensionality of the zeolite channels increases, the productivity of C_5_–C_11_ hydrocarbons is favored in the following sequence: HZSM-5 (3-dimensional) > HMCM-22 (2-dimensional) > HZSM-23 (1-dimensional). Therefore, by changing the type of zeolite, it is possible to adjust the ratio of nonaromatics to aromatics in gasoline-range hydrocarbons. When tested under the same conditions, HZSM-5 zeolites with MFI topology produced a higher proportion of aromatics (up to 61% of the gasoline fraction), while the HMCM-22 zeolite with MWW topology mainly generated nonaromatics (up to 46% of the gasoline fraction).^201^ In another study, Song et al.^140^ synthesized HZSM-5 via three different methods, including hydrothermal (-hy), dry gel (-dg), and phase transfer (-pt), and compared their performance in combination with 6.25Cu-Fe_2_O_3_. It should be highlighted that zeolite HZSM-5-c refers to commercial HZSM-5. It was observed that the phase transfer method provides the best HZSM-5 in terms of both aromatic selectivity (61.9%) in hydrocarbons and BTX proportion (54.2%) due to its abundant mesopores (Figure 17 (c)). Therefore, selectivity toward BTX can be tuned by selecting the synthesis method of HZSM-5 to enhance the morphology.^140^

**Figure 17:**
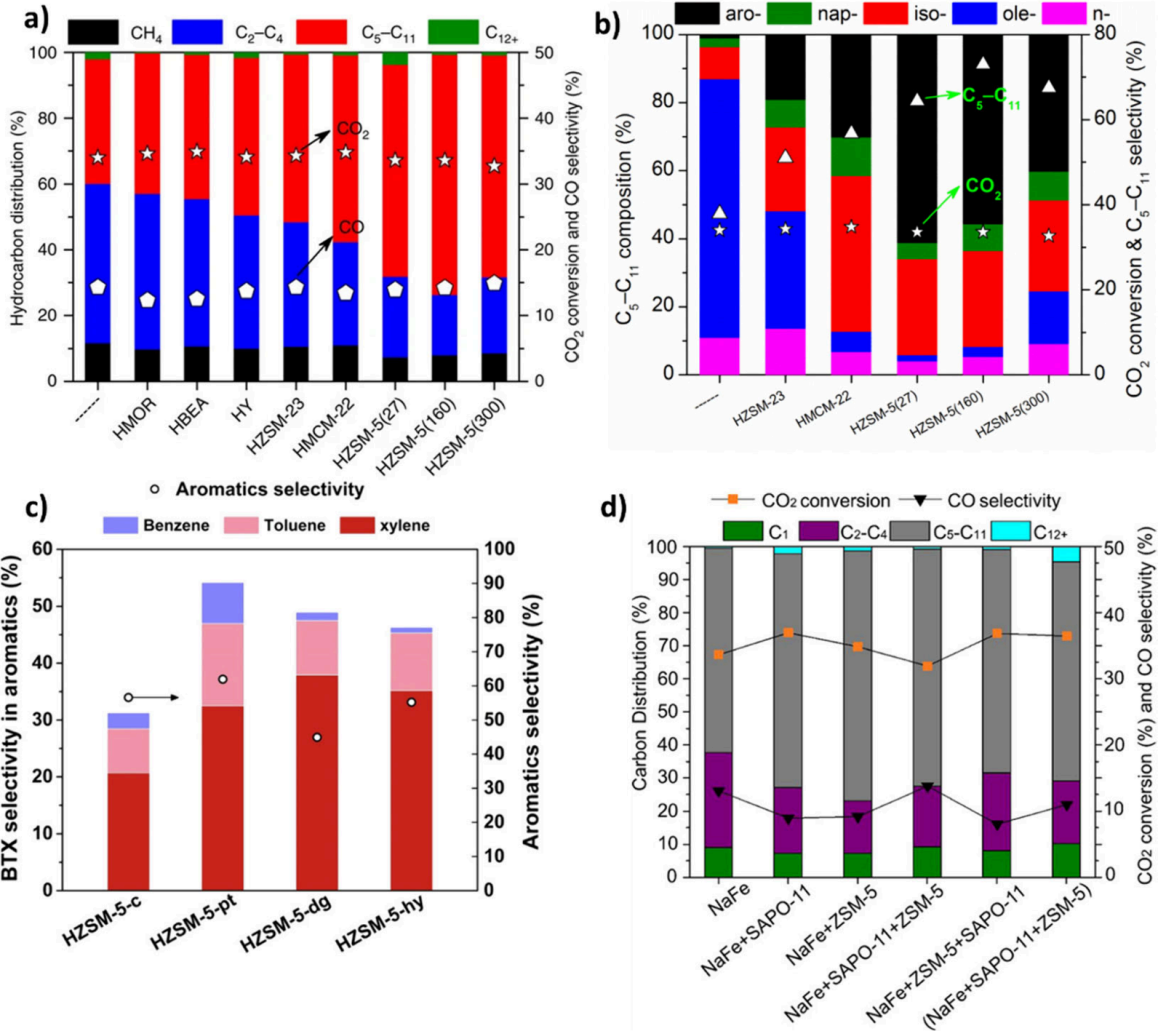
Effect of different zeolites on **a)** CO_2_ conversion and product selectivity, **b)** C_5_–C_11_ hydrocarbon distribution over Na-Fe_3_O_4_/Zeolite. Reproduced with permission from Wei et al.^201^ Copyright 2017, Nature Springer Publishing under [CC BY 4.0] license (Reaction conditions: 320 °C, 3 MPa, and 4000 mL h^–1^ g_cat_^–1^) (Note: aro-: aromatic; ole-: olefin; n-: *n*-paraffin; iso-:isoparaffin; nap-: naphthene). **c)** The effect of integrating different HZSM-5 with 6.25Cu-Fe_2_O_3_ on the aromatics selectivity and BTX distribution. Reproduced with permission from Song et al.^140^ Copyright 2020, ACS (Reaction conditions: 320 °C, 3 MPa, and 1000 mL g^–1^ h^–1^). **d)** Products distribution from hydrogenation of CO_2_ on NaFe coupled with SAPO-11 and ZSM-5 at different catalyst integration manners. Reproduced with permission from Noreen et al.^202^ Copyright permission obtained from ACS, 2020 (Reaction conditions: 320 °C, 3 MPa, and 6 (g h) mol^–1^).

Moreover, Noreen et al.^202^ introduced SAPO-11­(SiO_2_/Al_2_O_3_/P_2_O_5_ = 0.25/1/1) and/or HZSM-5(80) in different arrangements with Na-Fe to assess the effect of the proximity of Fe-oxide with different zeolite cages. Interestingly, the highest value of total C_5+_ hydrocarbon selectivity (76.9%, which contains mainly aromatics) was achieved when Na-Fe was stacked in dual-bed (D.B.) mode with HZSM-5. The high C_5+_ hydrocarbon selectivity is due to the relatively low Si/Al ratio and higher Brønsted acidity of the HZSM-5(80), necessitating a more considerable distance to avoid mutual poisoning of the active sites. However, substituting ZSM-5 with SAPO-11 resulted in lower iso-paraffin selectivity and a higher selectivity to C_2_–C_4_ olefins (14.7%). This difference was ascribed to the presence of parallel, one-dimensional channels in SAPO-11, resulting in a shorter retention time for the intermediates than the three-dimensional channels of HZSM-5. As a result, the formation of more branched hydrocarbons was facilitated in HZSM-5. Moreover, when both zeolites were combined with a Na-Fe_3_O_4_ catalyst (Na-Fe) in a triple-bed configuration or via physical mixing, C_5+_ formation decreased slightly while the formation of C_2_–C_4_ increased. It was observed that in Na-Fe+SAPO-11+ZSM-5 assembly, the selectivity toward aromatics was lower than that of Na-Fe+ZSM-5+SAPO-11, which was related to the easier transfer of intermediates from Na-Fe to ZSM-5 acidic sites. The production of aromatics was reduced, and more CH_4_ was produced on the catalysts integrated by the physical mixing of the three mentioned materials, compared to the triple-layer combination, which indicates the inappropriate proximity. By doubling the amount of ZSM-5 in triple beds, CH_4_ formation increased. At the same time, the aromatic proportion in the total C_5+_ products decreased considerably, which can be ascribed to the elevated density of acidic sites.^202^ The distribution of the products is depicted in Figure 17 (d).

Wang et al.^203^ conducted experiments by combining FeK1.5/HSG with various zeolites. The study revealed that the selectivity toward aromatics increased in the following order: HZSM-5 > HMCM-22 > Hβ > HY > SAPO-34. Among these zeolites, SAPO-34, with a pore size of 3.8 Å × 3.8 Å, produced the least aromatic hydrocarbons. This is due to the pore size being too small to easily allow even the smallest ethylene molecule (kinetic diameter of 3.9 Å) to access the acid sites. On the other hand, both HY and Hβ zeolites possess 12-membered large pores, with dimensions of 7.4 Å × 7.4 Å and 6.6 Å × 6.7 Å, respectively. Additionally, Hβ has an extra 12-membered small pore measuring 5.6 Å × 5.6 Å. The kinetic diameter of benzene is approximately 5.85 Å, with a width of around 5 Å, as calculated based on bond lengths and angles. Toluene and p-xylene have similar kinetic diameters. Due to these dimensions, Hβ zeolite exhibited higher selectivity for aromatic molecules than HY zeolite. Similarly, HMCM-22 shares structural similarities with HZSM-5, possessing two groups of 10-membered pores, measuring 4.0 Å × 5.5 Å and 4.1 Å × 5.1 Å, respectively. Although these pores are slightly smaller than those in HZSM-5 (5.1 Å × 5.5 Å and 5.3 Å × 5.6 Å), HMCM-22 still achieves the second-highest selectivity for aromatic compounds, as illustrated in Figure 18 (a). Figure 18 (b) compares the aromatic distribution over NaZSM-5(50), HZSM-5(50), and HMCM-22 combined with FeK1.5/HSG, all of which exhibit high aromatic selectivity. Notably, NaZSM-5(50) predominantly produces benzene and toluene, whereas HZSM-5(50) and HMCM-22 mainly form ethylbenzene and propylbenzene. Furthermore, HZSM-5(50) exhibits the highest selectivity for xylenes among these zeolites.^204^ This highlights that by utilizing a dual-layer combination of FeK1.5/HSG and different zeolites, the composition of aromatic products can be adjusted simply by changing the zeolite component, providing significant flexibility in the CO_2_ olefination–aromatization process.^203^

**Figure 18:**
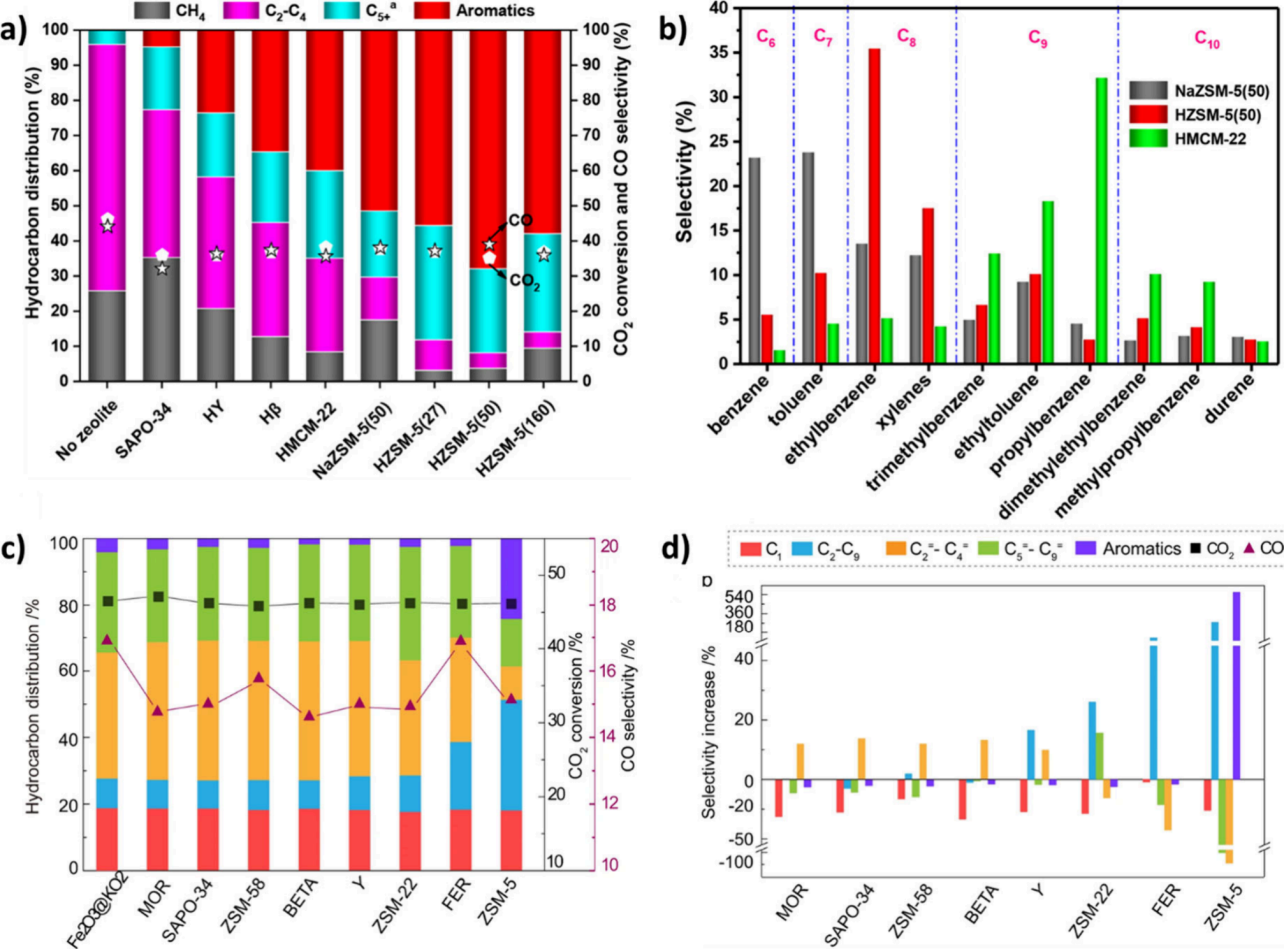
Effect of different zeolites on **a)** CO_2_ conversion and product selectivity and **b)** Aromatic distribution over FeK1.5/HSG|zeolites (Reaction conditions: 340 °C, 3 MPa, and 26,000 mL h^–1^g_cat_^–1^). Reproduced with permission from Wang et al.^203^ Copyright 2019, ACS. Catalytic performance of the Fe_2_O_3_@KO_2_/zeolite bifunctional material in the CO_2_ hydrogenation. **c)** CO_2_ conversion and product distribution. **d)** Detailed depiction of the change in selectivity of individual hydrocarbon groups upon the introduction of zeolites with respect to the standalone Fe/K catalyst (Reaction conditions: 375 °C, 3 MPa, and 10,000 mL h^–1^ g cat^–1^). Reproduced with permission from Ramirez et al.^205^ Copyright 2021, Springer Nature under [Creative Commons CC BY] license.

In another investigation, Ramirez et al.^205^ used Fe_2_O_3_@KO_2_ (physically mixed) integrated with various zeolites such as 1D ZSM-22, 2D MOR, FER and ZSM-58, 3D SAPO-34, BETA, ZSM-5, and Y in a D.B. manner. Combining a Fe_2_O_3_@KO_2_ catalyst with a zeolite decreased selectivity toward CO, with the lowest value observed using BETA zeolite. However, the CO_2_ conversion remained the same for all zeolites, indicating that none of them are capable of initially activating CO_2_, but able to consume CO (Figure 18 (c)). Moreover, it is observed that most zeolites (MOR, SAPO, ZSM-58, BEA, Y) resulted in a slight increase in the formation of light olefins. In contrast, ZSM-22, FER, and ZSM-5 increased the production of heavier olefins, paraffins, and aromatics, respectively. The decrease in CO selectivity by approximately 15% upon introducing zeolite was explained by the presence of multiple carbonylated species as reactive intermediates. Additionally, for most zeolites, there was an increase in selectivity toward light olefins by about 15%, as can be seen in Figure 18 (d).^205^

It is interesting to note that only ZSM-5, FER, and ZSM-22, which exhibit 10-member rings, did not follow this trend (of light olefin production) and exhibited the most effective confinement effect for the oligomerization of intermediates (Figure 19(a)). Accordingly, there are four distinct groups into which different zeolites can be classified based on their ability to incorporate CO: (i) those that produce light olefins (e.g., SAPO-34, BETA, MOR, ZSM-58, and Y), (ii) those that form long-chain olefinic hydrocarbons (e.g., ZSM-22), (iii) those where hydrogen transfer is predominant, leading to paraffin formation (e.g., FER), and (iv) those that facilitate aromatic formation (e.g., ZSM-5),^205^ as illustrated in Figure 19 (b).

**Figure 19:**
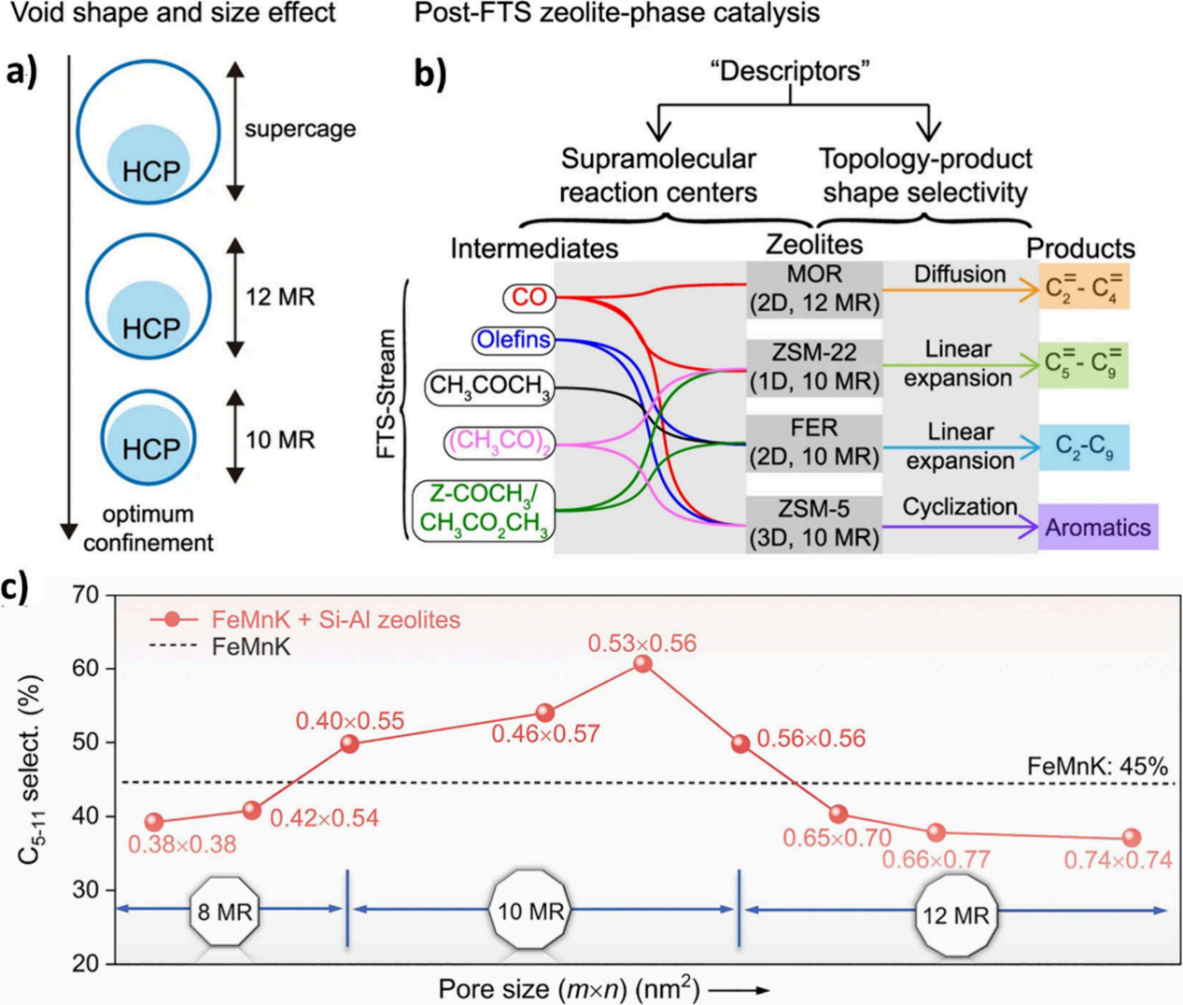
**a)** Schematic representation of the confinement effect in the presence of zeolites and **b)** the influence of zeolite topology on final product selectivity from various intermediates. Reproduced with permission from Ramirez et al.^205^ Copyright 2021, Springer Nature under [Creative Commons CC BY] license. **c)** The effect of zeolite pore size on C_5+_ selectivity. Reproduced with permission from Li et al.^86^ Copyright 2023, Elsevier.

Amoo et al.^206^ showed that the 1-D structure of HZSM-22 was favorable for the formation of heavier olefins and iso-paraffins, while the 3D structure of MFI enhanced the aromatization and isomerization. The effect of zeolite pore size on product distribution was also investigated by Li et al.,^86^ and it was revealed that C_5+_ selectivity over FeMnK integrated with 10 MR zeolites such as HMCM-22, HZSM-22, HZSM-48, and HZSM-5 was greater than 50%, as depicted in Figure 19 (c).

Recently, Lee et al.^207^ also showed that integrating Na-Fe_3_O_4_ with ZSM-5 resulted in more aromatics, while using MCM-22 generated a mixture of aromatic and naphthene. Additionally, they demonstrated that integrating iron oxide with an isomerization catalyst, such as PtWZ, could interestingly lead to the formation of iso-paraffins. It was revealed that using oxo-anions (WO_3_-ZrO_2_) that are active in the isomerization of C_4+_ reactions could be a promising alternative to zeolites that are prone to deactivation and ion leaching.^207^

### 5.2 Brønsted Acidity

In addition to the pore structure, the Si/Al ratio of the zeolite, which is an indication of the zeolite BAS, is another key parameter determining the distribution of hydrocarbon products in CO_2_ hydrogenation.^187^

A low Si/Al ratio leads to an increased concentration of BAS and enhances the cracking ability, resulting in a greater selectivity for light paraffins. Furthermore, an excessive amount of strong BAS can speed up coke formation, causing blockages in the zeolite pores and decreasing the selectivity for aromatics. On the other hand, a high Si/Al ratio results in low BAS density, which may not be enough for further oligomerization, aromatization, and isomerization of the intermediates, as confirmed in recent work by Wei et al.^194^ which used ZSM-5 with different Si/Al ratios ranging from 25 to 570. It was observed that in the presence of Na-ZSM-5(25), exhibiting no BAS, the selectivity of liquid hydrocarbons was negligible, while using H-ZSM-5 and increasing the Si/Al ratio to 160, a maximum C_5+_ selectivity of 74% was obtained (Figure 20 (a)). Moreover, the CO selectivity slightly decreased by increasing the BAS (i.e., decreasing the Si/Al ratio from 570 to 25), indicating the consumption of more CO and progress of FT rather than RWGS.^194^ By decreasing the ratio of Si/Al, the nonaromatic proportion of C_5+_ in the hydrocarbons decreased, while the production of C_2_–C_4_ paraffins increased, showing the cracking of C_5+_ hydrocarbons (Figure 20 (b)).^201^ The same Si/Al ratio, i.e., 160, was also found as the optimum ratio when Fe/C^208^ and CuFeO_2_ ^144^ were coupled with HZSM-5, indicating that moderate BAS is required in contact with Fe-based oxide to produce aromatic hydrocarbons. Accordingly, Jin et al.^208^ showed that the highest performance in terms of both CO_2_ conversion (33.2%) and C_5+_ selectivity (49%) could be achieved over Fe/C/ZSM(160). However, either stronger (Si/Al = 50) or weaker (Si/Al = 300) acidity was not a suitable match since more cracking occurred in the former, and the latter was not able to promote aromatization and isomerization reactions,^208^ as illustrated in Figure 20 (c). It was also suggested that the density of acid sites could affect product distribution. In this context, it was shown that with a higher zeolite weight (Fe-oxide/ZSM-5 (1/2)), the acidity of ZSM-5 can drastically affect the alkalinity of the oxide. Therefore, the distance between the oxide and the zeolite, particularly between Fe-C and ZSM-5, should be increased, as illustrated in Figure 20 (d).

**Figure 20:**
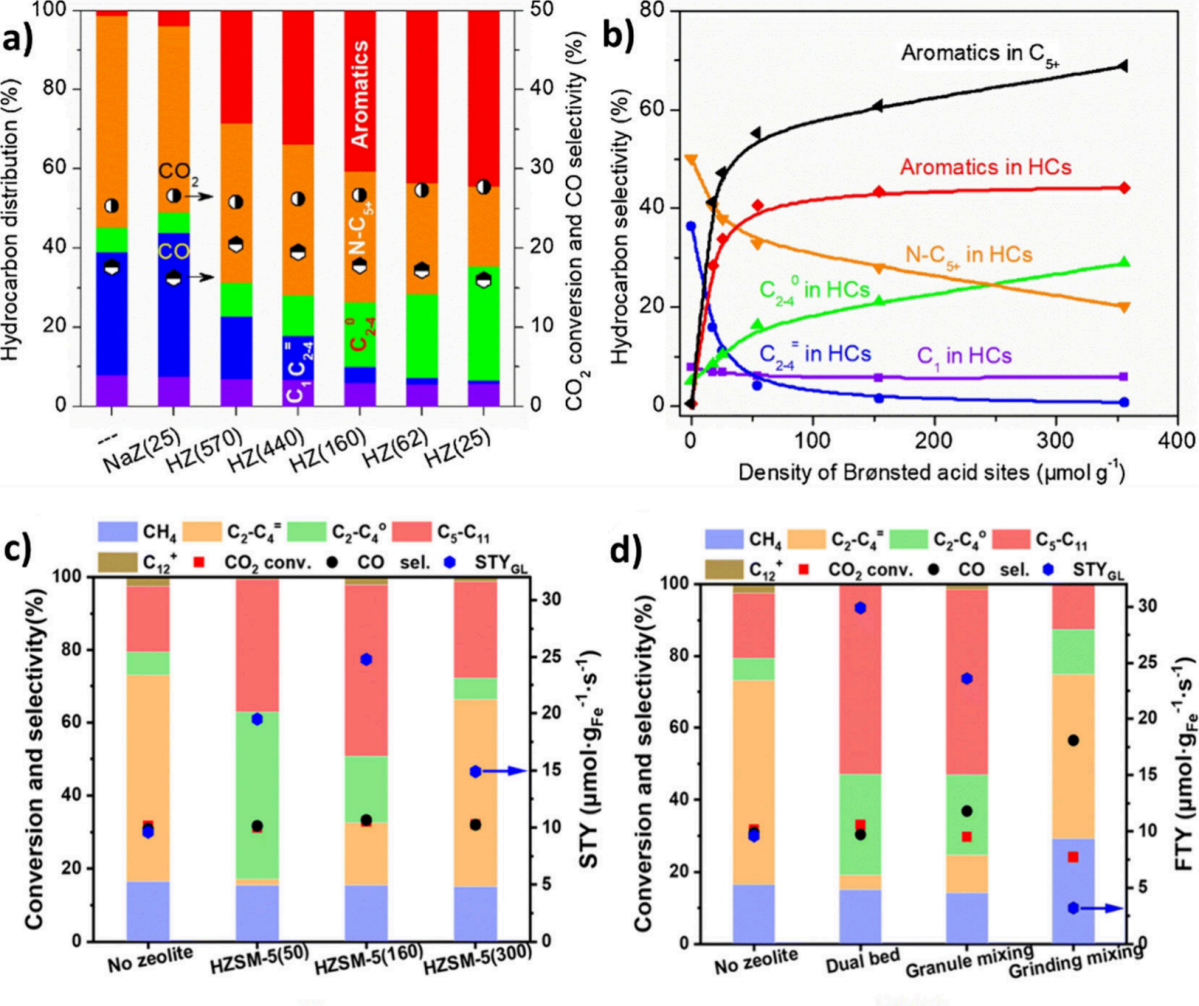
**a)** Catalytic performance of various NaFe/ZSM-5 catalysts with different acidities. **b)** Correlation between product selectivity and BAS density. N–C_5+_ represents the C_5+_ hydrocarbons, excluding aromatics (Note: C_2–4_^0^ and C_2–4_^=^ refer to the paraffins and olefins of C_2–4_ hydrocarbons, respectively). Reproduced with permission from Wei et al.^194^ Copyright 2021, Elsevier (Reaction conditions: 320 °C, 3 MPa, H_2_/CO_2_ = 2, and 4000 mL h^–1^). **c)** The effect of Si/Al ratio on the catalytic performance and **d)** influence of Fe-C/HZSM-5(160) proximity on the CO_2_ hydrogenation performance. Reproduced with permission from Jin et al.^208^ Copyright 2023, the Royal Society Publishing under [CC BY-NC 3.0] license. Reaction operating conditions: H_2_/CO_2_ = 3, 2 MPa, 320 °C, and 4000 mL h^–1^ g_cat_^–1^.

The same phenomenon was reported by Xu et al.^209^ while mixing ZSM-5 particles with different weights and sizes with Na-Fe, confirming the detrimental effect of high Brønsted acidity on the formation of carbides and reduced CO conversion to hydrocarbons via the FT path.^209^ It has been speculated that an appropriate ratio of Na-Fe to ZSM-5 would facilitate CO dissociation to C and, consequently, Fe_5_C_2_ formation. In the presence of a large amount of BAS, ZSM-5 can act as the electron acceptor and hinder electron donation from the promoted iron oxide, thereby preventing CO dissociation.

The choice of the promoter or other active elements may also play an essential role in determining the basicity of the Fe-based oxide and, in turn, the required BAS of the integrated zeolite. For instance, Cui et al.^210^ showed that a Si/Al ratio in the range of 23–27 would provide the best BAS for integration with the 4.25Na-ZnFeO_*x*_ catalyst (Figure 21 (a)). This can be ascribed to the higher basicity of the oxide induced by the incorporation of more Na along with Zn into the Fe-oxide. It was shown that the rise in the concentration of BAS density from 9 to 294 μmol g^–1^ resulted in a notable increase in the selectivity of aromatics while decreasing the selectivity of C_2_–C_4_ olefins and C_5+_ (nonaromatic) hydrocarbons. However, when the density of BAS exceeded 294 μmol g^–1^, the selectivity toward aromatics decreased, while the selectivity of light olefins, paraffins, and C_5+_ hydrocarbons increased (Figure 21 (b)).^210^

**Figure 21:**
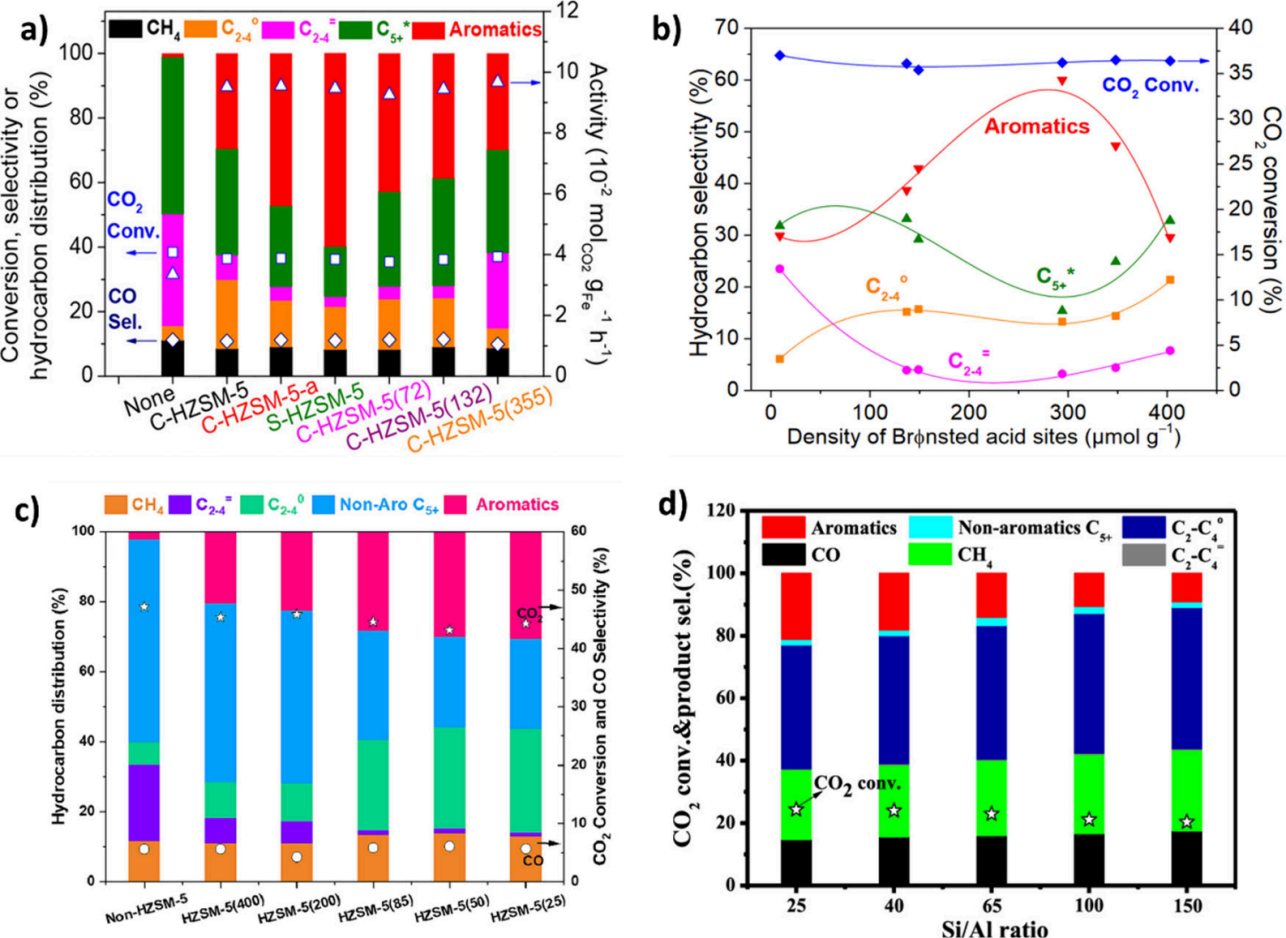
Effect of **a)** zeolite acidity and **b)** BAS density on the product distribution over Na-ZnFeOx/HZSM-5. Reproduced with permission from Cui et al.^210^ Copyright 2019, ACS (Reaction conditions: 320 °C, 3 MPa, and 4000 mL g_cat_^–1^ h^–1^) (Note: C_5+_* refers to C_5+_ products except for aromatics). **c)** The effect of different Si/Al ratios on the performance of K/Fe-Cu-Al and HZSM-5 catalysts. Reproduced with permission from Zhang et al.^211^ Copyright 2023, Elsevier. **d)** Effects of Si/Al ratios on CO_2_ hydrogenation performance using Fe-K/a-Al_2_O_3_ and HZSM-5 catalysts. Reproduced with permission from Dai et al.^212^ Copyright permission obtained from ACS, 2020 (Reaction conditions: H_2_/CO_2_ = 1, 400 °C, 3 MPa, and 3000 mL g_cat_^–1^ h^–1^).

In another study, Zhang et al.^211^ reported similar observations over KFeCuAl and HZSM-5 (Figure 21 (c)), which demonstrated that a lower SiO_2_/Al_2_O_3_ ratio (<85) promoted the aromatization of olefins. It was also observed that as the amount of BAS increased from 0 to 290 μmol/g, the data showed a consistent and continuous increase in the aromatics yield, rising from 0.9% to 12.8%. Nevertheless, the rate at which the aromatics yield increased began to slow down as the total amount of BAS increased. More specifically, there was a substantial increase in aromatics yield, going from 0.9% to 11.8% when the amount of BAS increased from 0 to 69 μmol/g. However, the increase in aromatics yield was only ∼1% when the amount of BAS was further increased to 290 μmol/g. This indicates that a specific threshold of acid sites is needed for aromatics formation, and having an excess amount of BAS (i.e., >69 μmol/g) was unnecessary, as it only resulted in marginal improvements in the aromatics yield.^211^

Furthermore, it has been revealed that the distribution of Fe-active species can be affected by whether the Fe-based oxide is used as bulk or supported. In fact, the choice of the support material can also affect the acid/base property of the Fe-based oxide. Dai et al.^212^ showed that increasing the Si/Al ratio led to a slight decrease in CO_2_ conversion and aromatic selectivity on Fe-K/a-Al_2_O_3_ integrated with HZSM-5, while selectivity toward CH_4_ increased. This suggested that a high Si/Al ratio, leading to the weak acidity of HZSM-5, was not favorable to producing aromatics from light unsaturated intermediates. The 21.1% selectivity toward aromatics achieved at a Si/Al ratio of 25 showed that strong zeolitic acid sites could play a crucial role in producing aromatics over supported Fe-based catalysts (Figure 21 (d)).^212^

### 5.3 Modification of BAS

The ZSM-5 is a preferred zeolite for the formation of aromatics via CO and CO_2_ hydrogenation in both FT and methanol-mediated pathways.^213^ However, some studies have highlighted the negative role of ZSM-5 strong acidic sites on catalyst performance. It has been confirmed that the Fe-based oxide, which is electron-rich and active for the FT, can favor CO dissociation, resulting in higher CO and CO_2_ consumption. However, BAS of ZSM-5 can act as electron acceptors that suppress electron transfer to CO and CO_2_ from Fe-based catalysts, preventing their conversion, especially when BAS are mixed with alkali-promoted Fe-based catalysts with inappropriate ratios of oxide/zeolite and/or in nonoptimized proximity. Moreover, alkali-based promoters can neutralize the BAS of zeolites.^209^ Several methods and conditions have been explored in recent years to regulate the BAS of HZSM-5, such as the passivation of external BAS and elemental substitution, which will be explained in the following sections.

#### 5.3.1 Passivation of External BAS

It was indicated that external acid sites promote bimolecular reactions like isomerization, H-transfer, and alkylation, while internal acid sites are responsible for monomolecular reactions such as cracking. Moreover, light olefins could be transferred to BTX over the surface BAS, leading to coke formation on the same sites.^214^ Within this framework, tetraethyl orthosilicate, formally named tetraethoxysilane (TEOS), a poor electro-conductible material, has been used in many cases as the silicate precursor to avoid the large surface BAS of ZSM-5. In fact, the Silicalite-1 (S-1) layer has been shown to avoid the large BAS of ZSM-5 via shielding the surface acid sites.^215^ Figure 22 (a) illustrates the multifunctional role of S-1 as a protective layer in a catalytic system, offering three critical pros: stabilizing the oxide phase, suppressing coke formation, and preventing ion migration. By keeping In^3+^ in place, S-1 ensures the stability and effectiveness of In_2_O_3_ during the catalytic conversion of CO_2_ and H_2_ into C_2+_ hydrocarbons. The S-1 layer also reduces coke deposition, which can deactivate the catalyst. This “one stone, three birds” approach improves the overall productivity and endurance of the catalyst system. Accordingly, Sibi et al.^216^ overcame the strong BAS via passivation of HZSM-5(12.5) surface acid sites using a similar strategy. The kinetic diameter of TEOS is larger than the pore openings of the HZSM-5, indicating that solely surface OH groups present on the original zeolite can interact with TEOS, creating Si–O–Al or Si–O–Si bonds, which then obstruct external acid sites. The strong surface BAS can help the alkylation of p-xylene (PX), benzene (B), and toluene (T), while passivation of the surface BAS sites hinders these reactions and increases the content of B, T, and PX in the final liquid products compared to the alkyl-substituted aromatics.^216,217^ Similar trends were reported by Cui et al.^210^ that showed coating the surface BAS of HZSM-5 with SiO_2_ altered the product distribution, while increasing the PX fraction in liquid hydrocarbons, as depicted in Figure 22 (b). Using the same approach, Xu et al.^218^ demonstrated that, in addition to suppressing the isomerization of *p*-ethyltoluene and PX, SiO_2_ coating could hinder light olefin hydrogenation to light paraffins, as depicted in Figure 22 (c) and (d).

**Figure 22:**
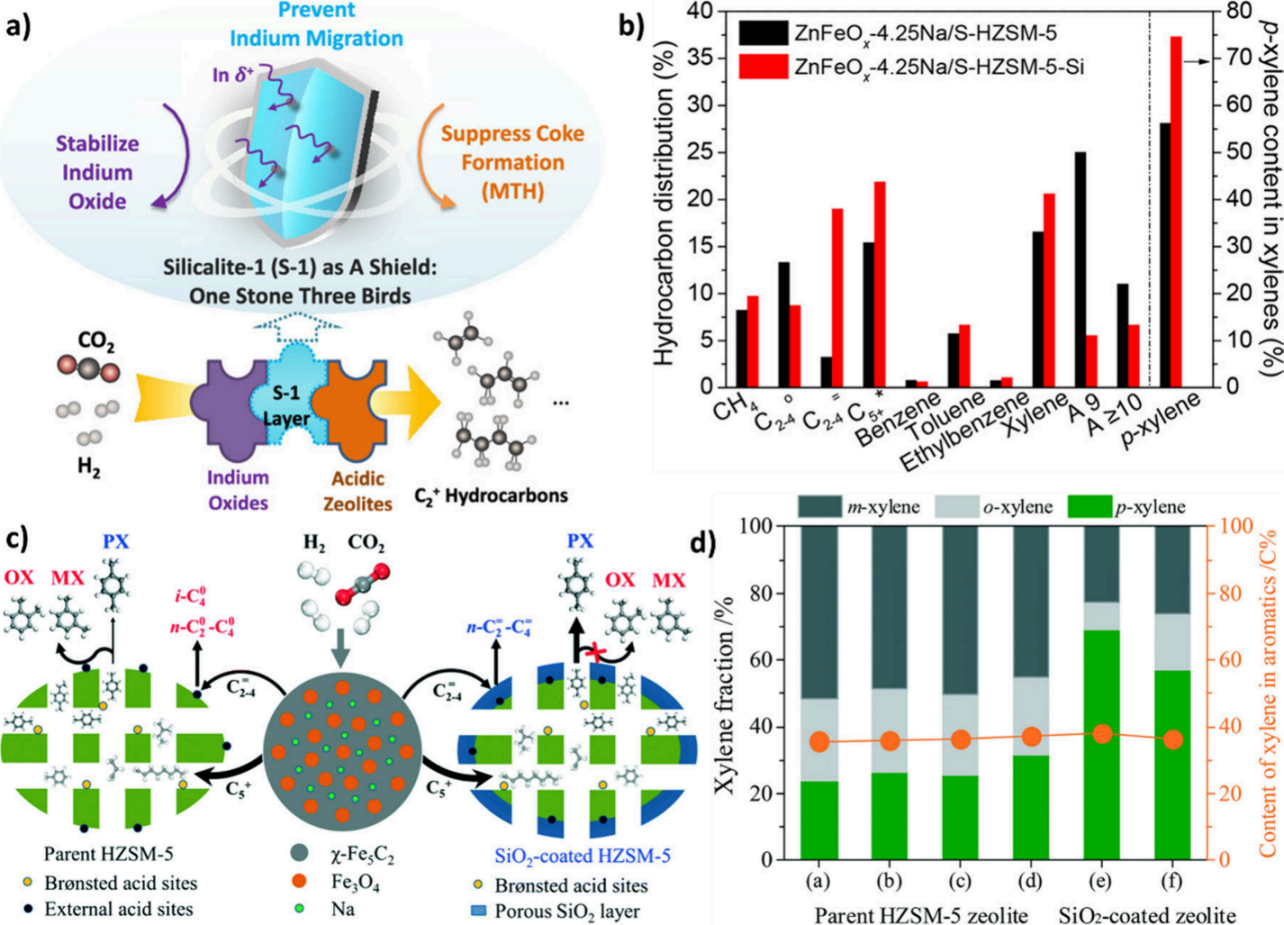
**a)** Schematic illustrating the role of Silicalite-1 in hindering ion migration to the zeolitic acid sites. Reproduced with permission from Xing et al.^215^ Copyright 2023, the ACS Publishing under [CC-BY-NC-ND 4.0.] license. **b)** The influence of H-ZSM-5 silylation on hydrocarbon distribution. Reproduced with permission from Cui et al.^210^ Copyright 2019, ACS. The impact of SiO_2_ coating on the isomerization reactions on the external surface of HZSM-5. **c)** Schematic representation and **d)** xylene distribution ((a) 2 MPa; (b) 1.5 MPa; (c) 1 MPa; (d) 1 MPa for 40 h; and over the composite Na/Fe and SiO_2_-coated HZSM-5 (Si/Al = 12.5) at 1 MPa for (e) 10 h and (f) 100 h). Reproduced with permission from Xu et al.^218^ Copyright 2019, RSC.

#### 5.3.2 Elemental Substitution

A widely used method to improve aromatics selectivity involves regulating the acidity of HZSM-5 by elemental substitution into the surface BAS through different treatments, which can play a crucial role in determining their overall behavior and catalytic activities.^219^ Using the ion-exchange method, Guo et al.^220^ modified the surface acidity of ZSM-5 by eliminating strong BAS. It was realized that the surface acidity of ZSM-5 can be modified to a certain extent depending on the metal ion. For example, it was revealed that ion-exchange with K and Na could effectively reduce surface BAS and consequently increase C_5+_ selectivity. However, K^+^ decreases the number of strong BASs more than Na^+^, which is ascribed to the more basic nature of K than Na.^220^ Interestingly, Cs substitution significantly reduces surface acidity, resulting in some unconverted light olefins in the products, as shown in Figure 23 (a). Liang et al.^221^ used different concentrations of NaOH solution to modify the acidity of HZSM-5. Using NH_3_-TPD, it was observed that acid strength increased with increasing the concentration of NaOH from 0 to 0.2 M, which led to an increase in aromatic selectivity from 25.4% to 35.1%. However, further increasing the concentration to 0.6 M resulted in structural collapse of the zeolite, reduced acidity, and reduced aromatic selectivity to 15.3%. This was ascribed to the enhanced mass transfer in zeolite pores, besides the tuned dispersion of Al through the zeolite structure.^221^ A similar phenomenon was observed by Wang et al.,^45^ who reported increased intensity of acid sites due to the shift of NH_3_-TPD peaks to higher temperatures as a result of the hollow zeolite structure formed by NaOH treatment. Therefore, increasing the concentration of NaOH from 0 to 0.2 M augmented aromatic selectivity from 30.9% to 50.2%, while further increasing the NaOH concentration to 0.4 M reduced selectivity toward aromatics (27.8%).^45^ Similarly, Cui et al.^210^ showed that using NaOH treatment significantly increased aromatic selectivity to 42.2% on the ZnFeO_*x*_-4.25Na/C-HZSM-5-a­(23) catalyst. This is accompanied by a decrease in the selectivity of CH_4_ and C_2_–C_4_ paraffins due to the reduced density of BAS. According to CO_2_-TPD, weak and medium basic sites increased via the introduction of Zn to HZSM-5(12.5). Moreover, NH_3_-TPD showed that compared to HZSM-5(12.5), ZnZSM-5(12.5) showed an increased number of weak acid sites, while medium and strong acid sites were reduced. This observation was linked to the ion exchange of BAS protons with Zn^2+^, acting like weak Lewis acid sites (LAS). These features resulted in tuning the properties of Na-FeAlO_*x*_/ZnZSM-5 to produce more BTEX and PX. As can be observed in Figure 23 (b), the addition of Zn (Zn­(NO_3_)_2_ solution, 1.0 M) significantly improves selectivity toward PX, BTEX, and total aromatics during CO_2_ hydrogenation. However, overdoping with Zn (1.5 M) adversely affected aromatic selectivity. The decreased ability of excess-Zn-doped HZSM-5(12.5) to produce aromatics is due to the excessive formation of less-active ZnO nanocrystallites, which impede the aromatization process.^216^

**Figure 23:**
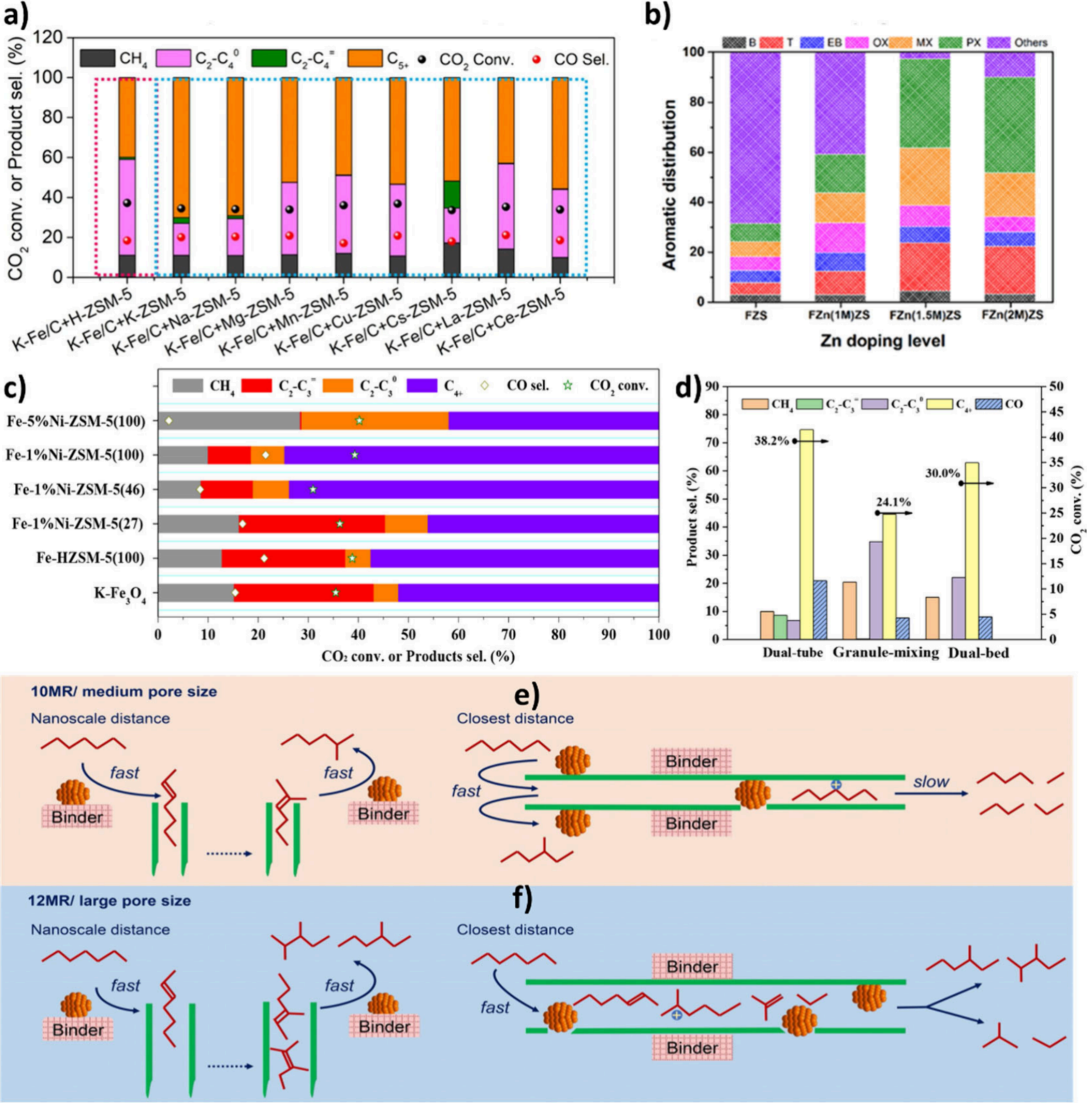
**a)** CO_2_ conversion and product selectivity on various bifunctional catalysts with different ion-exchange approaches (Reaction conditions: H_2_/CO_2_ = 2.5, 320 °C, mass ratio of K-Fe/C to zeolite = 1/3, 2.0 MPa, and 1200 mL g^–1^ h^–1^). Reproduced with permission from Guo et al.^220^ Copyright 2021, Elsevier. **b)** Aromatic distribution. Reaction operating conditions: 3.5 MPa; 370 °C, and 4000 mL g^–1^ h^–1^ (Note: B (benzene), Aro (aromatics), EB (ethylbenzene), Non-Aro C_5+_ (nonaromatic C_5+_), T (toluene), X (xylene), PX (p-xylene), OX (o-xylene), MX (m-xylene)). Reproduced with permission from Sibi et al.^216^ Copyright 2022, Elsevier. CO_2_ hydrogenation performance of **c)** K-Fe_3_O_4_ integrated with ZSM-5 with different acidities and **d)** K-Fe_3_O_4_ and 5%Ni-ZSM-5 (100) at different proximity levels (Reaction conditions: 320 °C, 3 MPa, and 2000 h^–1^). Reproduced with permission from Lu et al.^222^ Copyright 2022, ACS. The effect of the spatial arrangement of Pt on the binder or the acid sites of **e)** H-ZSM-22 and **f)** MOR. Reproduced with permission from Cheng et al.^223^ Copyright 2020, Wiley-VCH Verlag GmbH & Co. KGaA, Weinheim.

Lu et al.^222^ investigated the role of Ni-doped ZSM-5(100) on the CO_2_ hydrogenation performance of K-Fe_3_O_4_/xNiZSM-5­(100). They found that the addition of 1% Ni to HZSM-5 enhanced C_4+_ selectivity considerably, from 57.5% in K-Fe_3_O_4_/HZSM-5­(100) to 74.4% in K-Fe_3_O_4_/1%Ni-ZSM-5­(100). However, a further increase in Ni content to 5% reduced C_4+_ selectivity to 41.9%, even less than that of K-Fe_3_O_4_ (51.9%), as shown in Figure 23 (c). The presence of Ni increased the LAS while decreasing the BAS, which can be advantageous for converting light olefins into aromatic compounds. Nonetheless, an excess amount of acidic sites might cause the cracking of larger hydrocarbon products into lighter ones. Notably, 1%Ni-ZSM-5(100) exhibited a higher LAS amount and a lower BAS/LAS ratio based on pyridine adsorption FT-IR analysis.^222^ The influence of the distance between K-Fe_3_O_4_ and 5%Ni-modified HZSM-5(100) on CO_2_ hydrogenation was also investigated in the same study,^222^ as depicted in Figure 23 (d). By packing the catalysts in two reactors (dual-tube), the highest C_5+_ selectivity (74.7%) was obtained at 38.2% CO_2_ conversion. Decreasing the distance in dual bed (D.B.) mode reduced CO_2_ conversion and C_5+_ selectivity. Moreover, further decreasing the distance via granule stacking (G.S.) manner was detrimental to CO_2_ conversion, as the formation of CH_4_ and C_2_–C_3_ alkanes was promoted. The introduction of Ni species was found to enhance the quantity of LAS and reduce the amount of BAS, leading to a favorable condition for the aromatization of light olefins to produce aromatic compounds. Cheng et al.^223^ studied the hydro-conversion of *n*-heptane as an intermediate of CO/CO_2_ hydrogenation over different combinations of Pt/binder/zeolite to demonstrate the effect of metal/acid proximity on the product distribution. It was found that direct contact with Pt and zeolite resulted in more cracking products, whereas increasing the distance of metal/acid via loading Pt on a binder led to more isomerization than cracking, as shown in Figures 23 (e) and (f).^223^ Therefore, the closest proximity of Pt and acid sites in zeolite was found to be detrimental to isomerization reactions since the available zeolitic acid sites facilitated the cracking reactions on both 12 MR mordenite and 10 MR HZSM-22. However, the cracking was intensified when using MOR due to its longer diffusion length.^223^

## 6 Effect of Proximity of Active Sites

The strategic arrangement of catalytic processes sequentially, enabling cascade reactions, is highly significant in fine chemical synthesis.^224^ This integrated approach effectively minimizes the need for numerous isolation and purification steps, making it highly advantageous.^225^ Such control is decisive in obtaining a high yield of the target product while maintaining the catalyst lifetime.^226^ To manage the sequence of reactions and desired outcomes in the presence of reactants, intermediates, and products, the configuration of tandem catalysts holds significant importance in facilitating diverse reactions simultaneously under identical conditions.

### 6.1 Factors Contributing to the Proximity

Generally, integrating oxide and zeolite active sites in very close contact, such as powder mixing (using mortar or ball-milling), results in interferences that modify the electronic properties of the catalyst with an adverse effect on its performance and stability.^227,228^ Accordingly, granule mixing has been used to provide a balance between compatibility and proximity.^57^ However, the mentioned integration manners result in random and nonuniform distribution of oxide and zeolite, which hinder their availability based on the desired reaction sequence, and, in turn, the target product selectivity cannot be achieved. A dual-bed (D.B.) configuration, where Fe-based catalysts and zeolites are located at the top and bottom of the catalytic bed, respectively, has been used as an alternative to control the order of RWGS and FT reactions. However, in the D.B. mode, the oxide and zeolite active sites are so distant, which can promote alternative reaction pathways, such as further hydrogenation of intermediates producing undesired byproducts.^229,230^ If the active sites are too distant from each other, it can cause diffusion limitations, diminishing the reaction efficiency and impeding the formation of heavy hydrocarbons.^231,232^ Another alternative is employing consolidated zeolite structures such as honeycomb and open-cell foams or sponges, which allow tailoring the shape and size of the macropore system.^233,234^ However, the distance between the metal oxide and the zeolite cannot be adjusted appropriately in these cases since the oxide should be integrated randomly inside the zeolite pores. To overcome this challenge, encapsulated structures, such as core–shell and yolk–shell, have been utilized as emerging alternatives. This design allows the integration of Fe-based oxide and zeolite in the ordered architecture while providing precise control of the spatial arrangement of active sites at nanoscale.^235^

It is important to note that the density and strength of basic sites, which are affected by the incorporation of promoter and/or second element as described in section 4, and acidic sites, which can be modified by different treatments as explained in section 5.3,^236–238^ are critical in determining proximity. A higher active site density increases the likelihood of reactant molecules encountering neighboring sites, promoting preferred reactions and yielding higher hydrocarbons. However, excessively high active site density at close proximity can lead to overcrowding, detrimental interactions, potential poisoning, and deactivation due to ion migration and site blocking.^239^ Thus, finding the right balance in active site density is necessary for optimizing catalyst performance in CO_2_ hydrogenation reactions.^240,241^

Another important factor is the pore/cavity structure and size of zeolites, as explained in section 5.1. These factors influence the diffusion and mass transfer of reactants and intermediates within the catalyst pores, thereby altering the product distribution. Figure 24 illustrates the influential factors mentioned above in determining the active site proximity.

**Figure 24:**
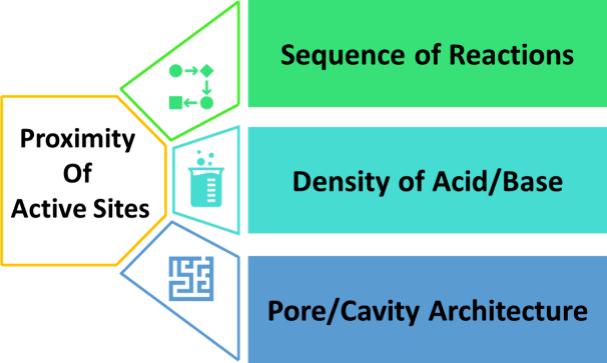
Illustration of the influential factors determining active site proximity (designed by authors).

Considering the above explanations, it can be concluded that the appropriate distance between active sites enables efficient coupling of reaction intermediates, facilitating the growth of hydrocarbon chains and influencing the relative rates of these competing reactions. Ensuring the active sites are optimally spaced promotes the preferred pathways, forming desired hydrocarbons, thereby enhancing their selectivity. In addition, having active sites in optimum proximity can lead to synergistic effects that emerge from cooperative interactions between neighboring active sites, where one site enhances the reaction at another or stabilizes reaction intermediates.^242,243^ Therefore, it can be concluded that the distance between the neighboring active sites should be close enough to facilitate the transport of intermediates to zeolite pores for further reactions on the one hand, and far enough to inhibit the detrimental interactions and mutual poisoning of basic and acidic sites on the other hand.^244^ However, based on the synthesis method and integration manner, which will be described in the following sections, the reaction intermediates and, consequently, the product distribution can be considerably altered.

Figure 25 (a) illustrates various methods for synthesizing bulk and supported Fe-based catalysts, highlighting their specific features. Moreover, Figure 25 (b) demonstrates the possible integration manners of Fe-based oxides and zeolite for the CO_2_ hydrogenation process that have been reported so far in the literature.

**Figure 25:**
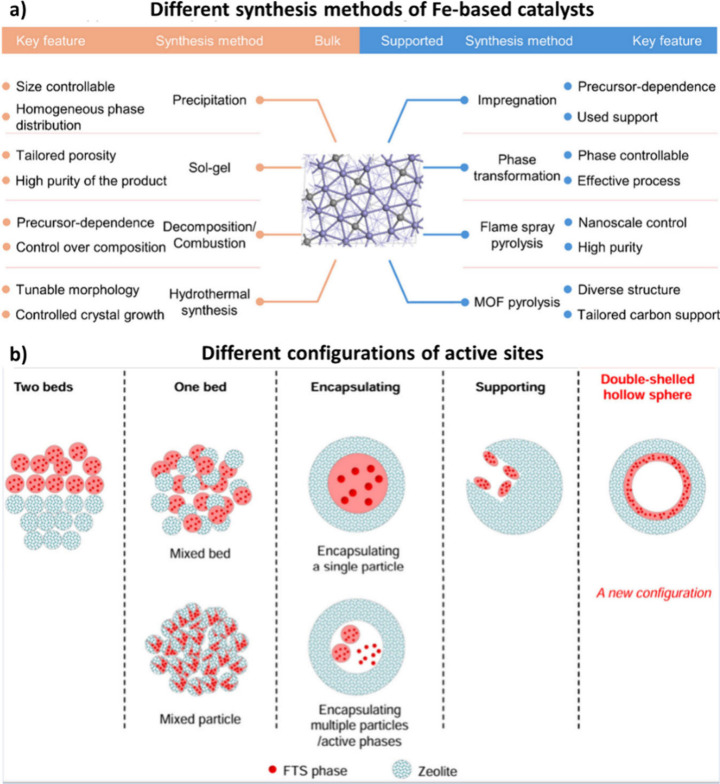
**a)** Different synthesis methods of Fe-based catalysts. Reproduced with permission from Yang et al.^245^ Copyright 2024, the ACS Publishing under [CC BY 4.0] license, and **b)** different integration manners of Fe-based oxides and zeolites for CO_2_ hydrogenation. Reproduced with permission from Nawaz et al.^246^ Copyright 2024, ACS.

### 6.2 The Influence of Synthesis Method

However, practical control of the mentioned distance, which can be tuned by certain synthesis methods, remains a matter of controversy. Sol–gel synthesis, coprecipitation, and atomic layer deposition methods can be used to control the active site placement to some extent.^235^ Recently, other methods have been used to control the proximity of active iron species during catalyst synthesis. Yao et al.^162^ prepared the Fe-Mn-K catalyst with the organic combustion method (OCM) using different organic agents, such as citric acid, EDTA, salicylic acid, oxalic acid, tartaric acid, DTPA, HEDTA, and NTA. It was observed that these agents facilitated the formation of nanostructured by acting as chelating agents. Generally, the catalysts synthesized via the mentioned method exhibited smaller crystallite sizes and better performance than the catalyst prepared without organic agents. It was indicated that using organic solutions resulted in a homogeneous solution due to the close proximity between metal precursors, which hindered aggregation or precipitation. Additionally, the aggregation of nanoparticles can be controlled by using the appropriate agent. Catalysts prepared using the OCM of citric acid showed a 38.2% CO_2_ conversion, while those synthesized without an organic agent showed only a 28.6% CO_2_ conversion.^162^ In another study, Yu et al.^247^ synthesized Fe-K/γ-Al_2_O_3_ via three methods, i.e., reverse microemulsion (RME) method, precipitation on RME-synthesized alumina, and precipitation on commercial alumina. It was found that the particle size in spent samples was smaller for RME Fe/γ-Al_2_O_3_ (6.7 nm) and Fe/RME-γ-Al_2_O_3_ (8.5 nm) compared to that of Fe/γ-Al_2_O_3_ (10.3 nm). Smaller particles provided uniform distribution, which facilitated reduction and carburization and helped the formation of heavier hydrocarbons as well. In the RME method, the particle size was limited since the nanodroplets, which act as nanoreactors, restrained the reactant amount^247^ and could promote closer proximity of active components. However, a thorough study of their stability under harsh reaction conditions and scalability remains elusive. Furthermore, exploiting scalable techniques, such as structured supports, spray pyrolysis, and industrial impregnation, can help regulate the proximity while enhancing mass and heat transfer. In addition, innovative strategies, like designing stable supports and encapsulating materials, are required to maintain the spatial arrangement of active phases under industrially relevant operating conditions.^235^

#### 6.2.1 Carbon Confinement Effect

One of the leading research objectives that has remained controversial is adjusting the proximity and ratio of Fe-oxide and Fe-carbide species via altering and optimizing the synthesis methods to enhance the CO_2_ hydrogenation performance of Fe-based catalysts.^248^ To this end, carbon-coated Fe-based catalysts have been found to provide Fe-oxide and Fe-carbide active sites in close proximity, offering a confinement effect. For instance, Luo et al.^249^ synthesized graphite-wrapped Fe_3_O_4_-FeCx catalysts using resin and varying amounts of copolymer P123 (Figure 26 (a)). It was demonstrated that the confinement effect of graphite layers was crucial in suppressing sintering and agglomeration, as well as shielding the active sites from water molecules. Accordingly, the highest selectivity of light olefins (45.1%) and C_5+_ (23.1%) hydrocarbons at 48% CO_2_ conversion could be achieved over the P-1.2 catalyst (Figure 26 (b)).^249^ Weber et al.^250^ used an iron polymeric complex during resorcinol-formaldehyde polymerization, which was followed by carbonization and synthesized carbon nanosphere (CNS) encapsulated Fe catalysts (Figure 26 (c)). It was indicated that a more reduced state of iron species could be retained within the nanocavities of the CNS, resulting in a favorable balance of iron-oxide and iron-carbide during CO_2_ hydrogenation. Moreover, the conversion increased with augmented temperature and decreased with reduced H_2_/CO_2_ ratios (Figure 26 (d)).^250^ Fu et al.^251^ could produce a FeNaC-N_2_ catalyst via the thermal decomposition of FeNa-EDTA in N_2_ (Figure 26 (e)). The catalyst provided 63% selectivity to olefins at 36.9% CO_2_ conversion (Figure 26 (f)), and the close proximity of N_2_ and carboxylate groups was found to be responsible for high CO_2_ adsorption and low CO and CH_4_ selectivity.^251^

**Figure 26:**
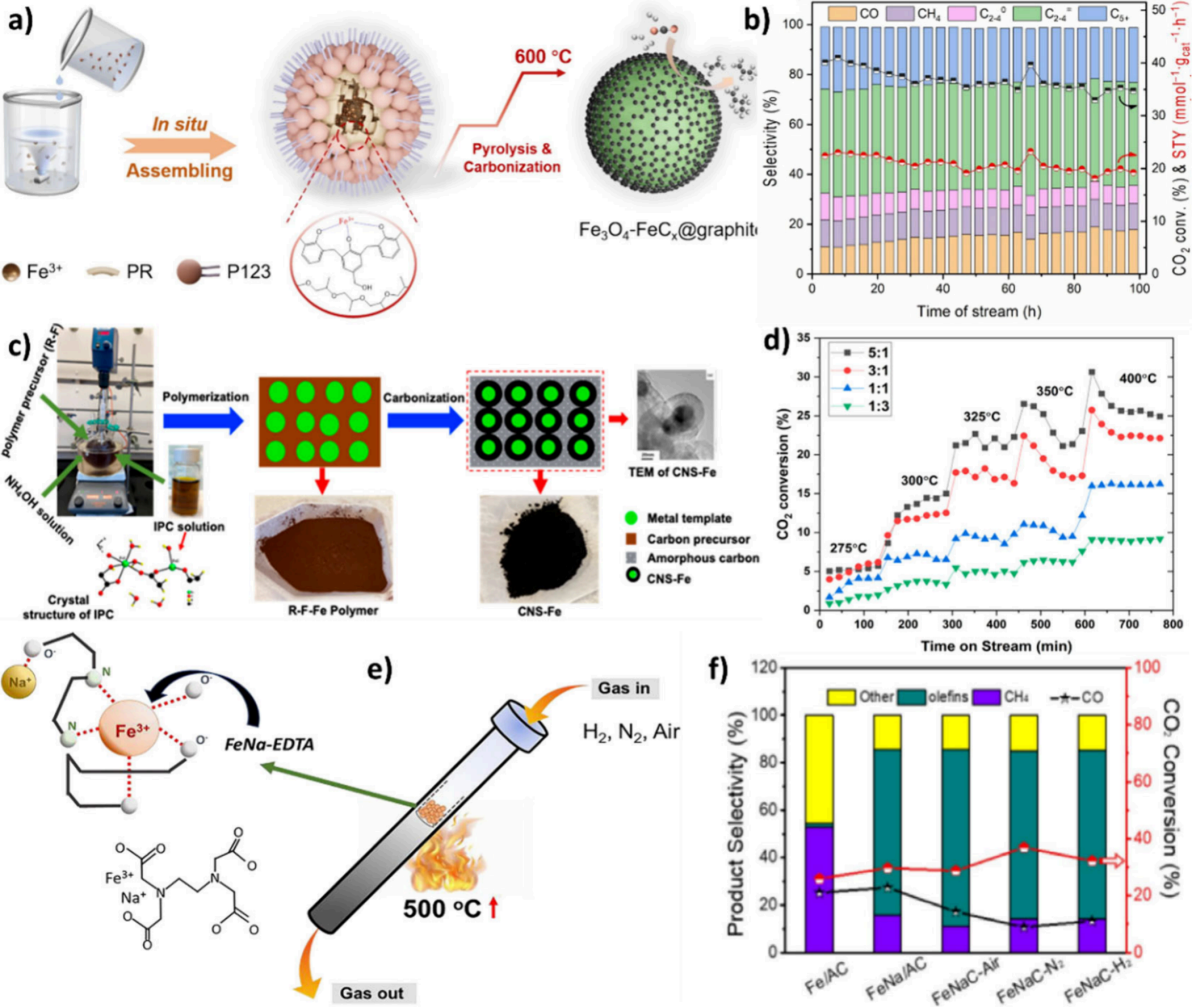
**a)** Schematic illustration of the synthesis procedure of Fe_3_O_4_-FeCx@graphite. **b)** Stability test of Fe_3_O_4_-FeCx@graphite at 320 °C, 3 MPa, and 12000 mL g^–1^ h^–1^. Reproduced with permission from Luo et al.^249^ Copyright 2024, Elsevier. **c)** Schematic representation of preparation methods for the synthesis of the CNS-Encapsulated Iron core–shell catalysts. **d)** The CO_2_ conversion over CNS-Fe (7.2 wt %) at different H_2_/CO_2_ ratios, different temperatures, atmospheric pressure, and 24000 mL g^–1^ h^–1^. Reproduced with permission from Weber et al.^250^ Copyright 2022, ACS. **e)** The synthesis method of FeNaC composites using FeNa-EDTA and **f)** CO_2_ hydrogenation performance of different catalysts at 320 °C, 3 MPa, and 20 mL/min. Reproduced with permission from Fu et al.^251^ Copyright 2022, Elsevier.

Moreover, Qi et al.^252^ prepared a series of carbon-coated catalysts via pyrolysis of ferrous fumarate under N_2_ at different temperatures and showed that the formation of more graphitic shells on NaFe-N2-400 resulted in easier carbide formation, while either lower or higher temperatures led to the formation of more amorphous carbon, which was undesirable.^252^ In another study, core–shell Fe-based catalysts were synthesized via dopamine polymerization, followed by carbonization under an N_2_ atmosphere at 500 °C. It was observed that the confinement effect of carbon coating could prevent the formation of long-chain hydrocarbons in the cavities due to the time-consuming nature of these reactions. In addition, the confinement effect of the carbon shells reduced H_2_ adsorption, which, in turn, hindered the hydrogenation of light olefins (Figure 27 (a)). However, increasing the number of carbon coatings to 3 resulted in aggregation and a reduction in the BET surface area. Moreover, the CO_2_ conversion reduced over this catalyst compared to the catalyst with one and two carbon shells (Figure 27 (b)) due to the lower exposure of active sites for the RWGS reaction.^253^ Zhang et al.^254^ synthesized Fe_3_C confined by F-doped mesoporous carbon using PTFE, F127, and PR (Figure 27 (c)). It was found that F hindered the active site agglomeration and protected FeC_*x*_ from oxidation by water, which resulted in improved catalyst stability. Further, PTFE induced the formation of Fe_3_C and promoted pore channel generation, which showed stable performance at long time-on-stream (Figure 27 (d)).^254^ The same group prepared Fe-based catalysts dispersed in mesoporous carbon via solvent evaporation-induced self-assembly (EISA) followed by pyrolysis. It was shown that the improved dispersion of the active site, along with the tuned ratio of FeO_*x*_ to FeC_*x*_, could be achieved via regulating pyrolysis conditions and additives such as promoters and N_2_ doping.^159,255^

**Figure 27:**
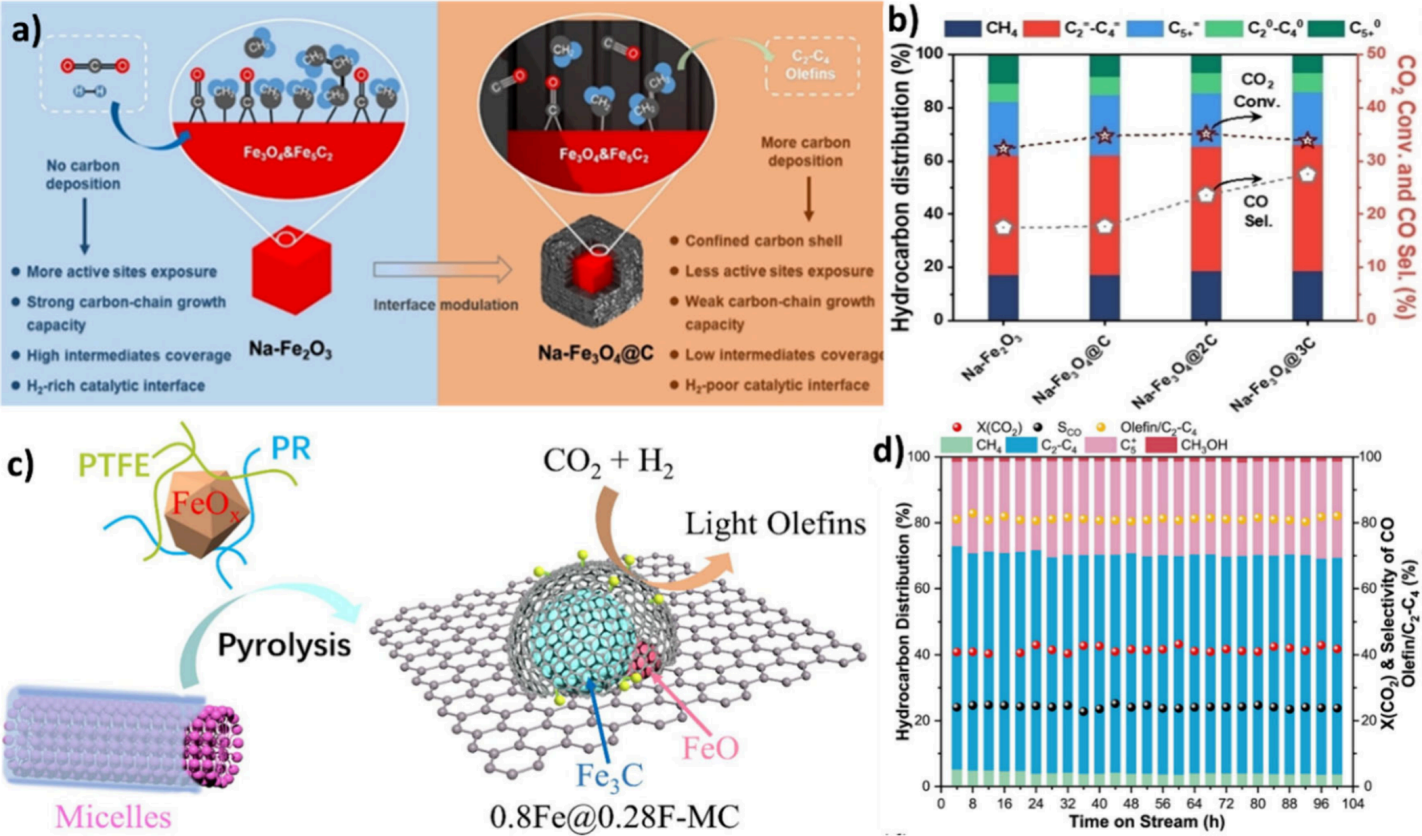
**a)** Comparing the features of Na-Fe_2_O_3_ and Na-Fe_2_O_3_@C. **b)** The CO_2_ hydrogenation performance of Na-Fe_2_O_3_ and Na-Fe_3_O_4_@*x*C (*x* = 1, 2, or 3) at 320 °C, 3 MPa, and 9000 mL g^–1^ h^–1^. Reproduced with permission from He et al.^253^ Copyright 2023, Wiley-VCH. **c)** Schematic illustration of Fe@F-MC synthesis via in situ pyrolysis. **d)** The stability of 0.8Fe@0.28F-MC + 0.02K, at 320 °C, 3.0 MPa, and 12000 mL g^–1^ h^–1^. Reproduced with permission from Zhang et al.^254^ Copyright 2023, Elsevier.

Recently, the synthesis of carbon-confined iron nanoparticles (Fe@C) by pyrolysis of MOFs has attracted much attention.^256–258^ In this method, the MOF can be pyrolyzed under an inert atmosphere like N_2_ at different temperatures and durations, which results in the formation of encapsulated metal nanoparticles in a porous carbonaceous matrix (Figure 28 (a)).^259–261^ It was revealed that by adjusting the pyrolysis temperature and duration, the particle size and structure of the carbon layer, including continuity and thickness, can be controlled (Figure 28(b)). Accordingly, by increasing the temperature or duration of pyrolysis, the particle size increased, while a thinner carbon layer with more defects was achieved.^262,263^ It has been found that more defects in graphitic carbon layers facilitated the diffusion of gas molecules into the iron species. However, this could result in less confinement effect, oxidizing the active iron phases and, in turn, reducing the light olefin and C_5+_ selectivity.^262^ However, it is noteworthy that the external carbon layer that enveloped the Fe nanoparticles in K/Fe-C (carbon-confined Fe derived by MIL-100­(Fe) pyrolysis) could effectively increase the catalyst stability by inhibiting the agglomeration and migration of Fe species compared to that of K/Fe-AC (Fe supported on AC).^264^

**Figure 28:**
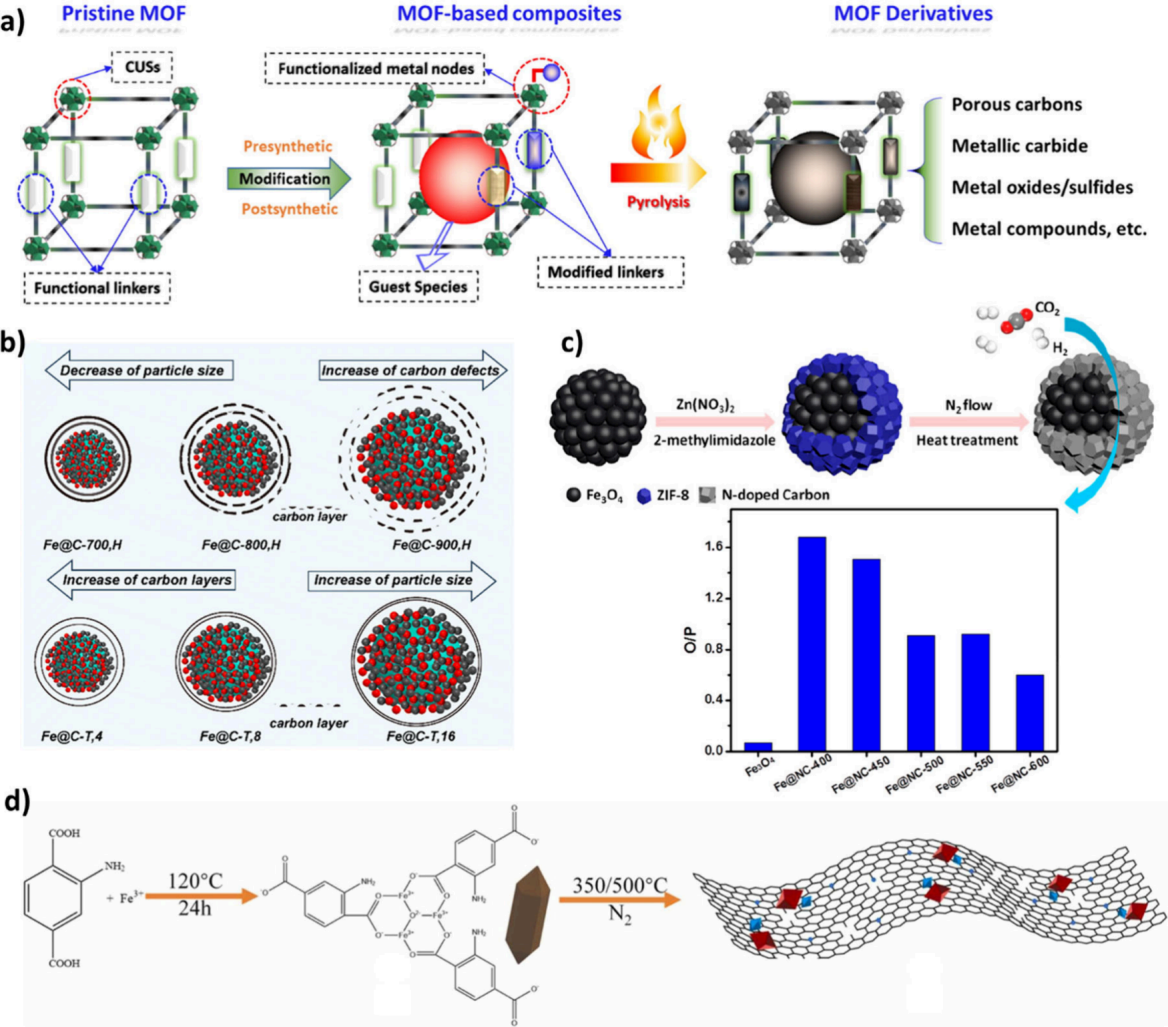
**a)** Schematic illustration showing the formation of carbon/metal-based porous catalysts by means of pyrolysis of MOF precursors and their active site locations. Reproduced with permission from Cui et al.,^261^ Copyright 2019, Elsevier. **b)** Structural changes of the Fe@C catalysts during pyrolysis based on the time and temperature changes. Reproduced with permission from Jiang et al.^262^ Copyright 2025, RSC. **c)** Schematic representation of Fe@NC synthesis and Olefin to paraffin ratio of C_2_–C_4_ hydrocarbons. Reproduced with permission from Liu et al.^265^ Copyright 2019, ACS. **d)** Preparation method of Fe/C@NC-350/500 from Fe-MOF precursors. Reproduced with permission from Xu et al.^260^ Copyright 2024, Elsevier.

Jiang et al.^262^ showed that defects could be formed in carbon layers via increasing pyrolysis temperature of MIL-100­(Fe)-based MOF from 700 to 800 °C, which enhanced CO_2_ conversion to 49% and reduced CO selectivity to 15%. Moreover, a very long pyrolysis time at a high temperature (900 °C) resulted in iron aggregation, which reduced CO_2_ conversion and increased CH_4_ formation due to insufficient exposure to active sites.^262^ It was demonstrated that adjusting the pyrolysis temperature of Fe_3_O_4_@ZIF can alter the morphological characteristics of the resultant Fe@NC, resulting from the formation of porous N_2_-doped carbon (NC) and carbide, thereby increasing the CO_2_ adsorption capacity. It was also found that, at 400 and 450 °C, the Fe_3_O_4_ particles could be surrounded by the NC layer; however, at higher temperatures, massive monoblocks of Fe_3_C were observed. The better performance of the former catalysts in terms of olefin/paraffin ratio (Figure 28 (c)) revealed the role of morphology and, thus, the configuration of active C and Fe particles in the catalyst.^265^ Xu et al.^260^ synthesized the Fe/C–K@NC-X catalysts (X representing the pyrolysis temperature) by pyrolyzing NH_2_-MIL-88B at different temperatures under N_2_ (Figure 28 (d)). It was observed that Fe/C-K@NC-350-500, formed via a two-step pyrolysis process, demonstrated the highest CO_2_ conversion (35.1%) and C_2_–C_4_ olefin selectivity (37.7%), as well as the moderate CO selectivity (28.1%). This was attributed to the presence of both Fe-oxide and Fe-carbide, as well as a portion of the MOF structure in close vicinity. However, in the Fe/C-K@NC-600 and Fe/C-K@NC-350-600, only iron carbide could be observed in the XRD patterns of the spent samples. Therefore, the lack of Fe_3_O_4_ inhibited the CO_2_ activation and the RWGS reaction.^260^

However, to precisely determine the improvement provided by Fe@C encapsulated catalysts and to ascertain whether these enhancements outweigh the synthesis complexity and increase in manufacturing cost, appropriate benchmarking investigations of catalysts are necessary.^235^

#### 6.2.2 Architecture-Enhanced Mass and Heat Transfer

Furthermore, it was found that, in addition to the chemical properties, such as the coordination environment and electronic structure of the Fe-based catalysts, the architecture-derived mass and heat transfer properties can also affect the performance and stability of the catalyst. This can be achieved by modifying the intermediate density on the active sites by altering their geometry through 3D printing technology. Additionally, carbon decomposition and active site aggregation can be suppressed via architecture-enhanced heat transfer.^266^ In addition, the mass transfer limitations due to low bed voidage in granules and pellets can be prohibited by using appropriate 3D structures. Moreover, by changing channel spacing, cell density, and wall thickness through different geometries, transport properties can be enhanced.^267^ Furthermore, by investigating the features of CO_2_ hydrogenation catalysts, such as promoters, supports, and zeolites, it has been concluded that the configuration of active sites has to be considered in designing effective catalysts. 3D printing can help integrate the active components in diverse structures in preferred configurations.^268,269^

However, most studies have been conducted to use 3D printing catalysts in the field of CO_2_ methanation,^270^ and heavy hydrocarbon production using this emerging technology is still in its infancy. Nevertheless, some researchers have used 3D-printed ZSM-5 to produce higher hydrocarbons via the methanol-mediated route.^271,272^ Recently, Wang et al.^266^ prepared 3D monoliths with different configurations denoted as Na-Fe@C-3D-sta (staggered), Na-Fe@C-3D-str (straight), and Na-Fe@C-3D-spi (spiral) (Figure 29 (a)) for the CO_2_ hydrogenation reaction. It was shown that the Na-Fe@C-3D-spi could improve olefin formation and hinder extra hydrogenation of intermediates due to the enhanced mass transfer (Figure 29 (b)). Moreover, the catalyst maintained its performance after 50 h of reaction without any loss in activity.^266^ In another study, Wei et al.^273^ prepared three self-catalytic reactors (SCR) via the metal 3D printing method, denoted as Fe-SCR, Co-SCR (Figure 29 (c)), and Ni-SCR, for CO_2_ hydrogenation, FTS, and dry methane reforming. The Fe-SCR and Co-SCR demonstrated high catalytic performance, along with stability, under high temperatures and pressures for the production of liquid hydrocarbons (Figure 29 (d) and (e)).^273^

**Figure 29:**
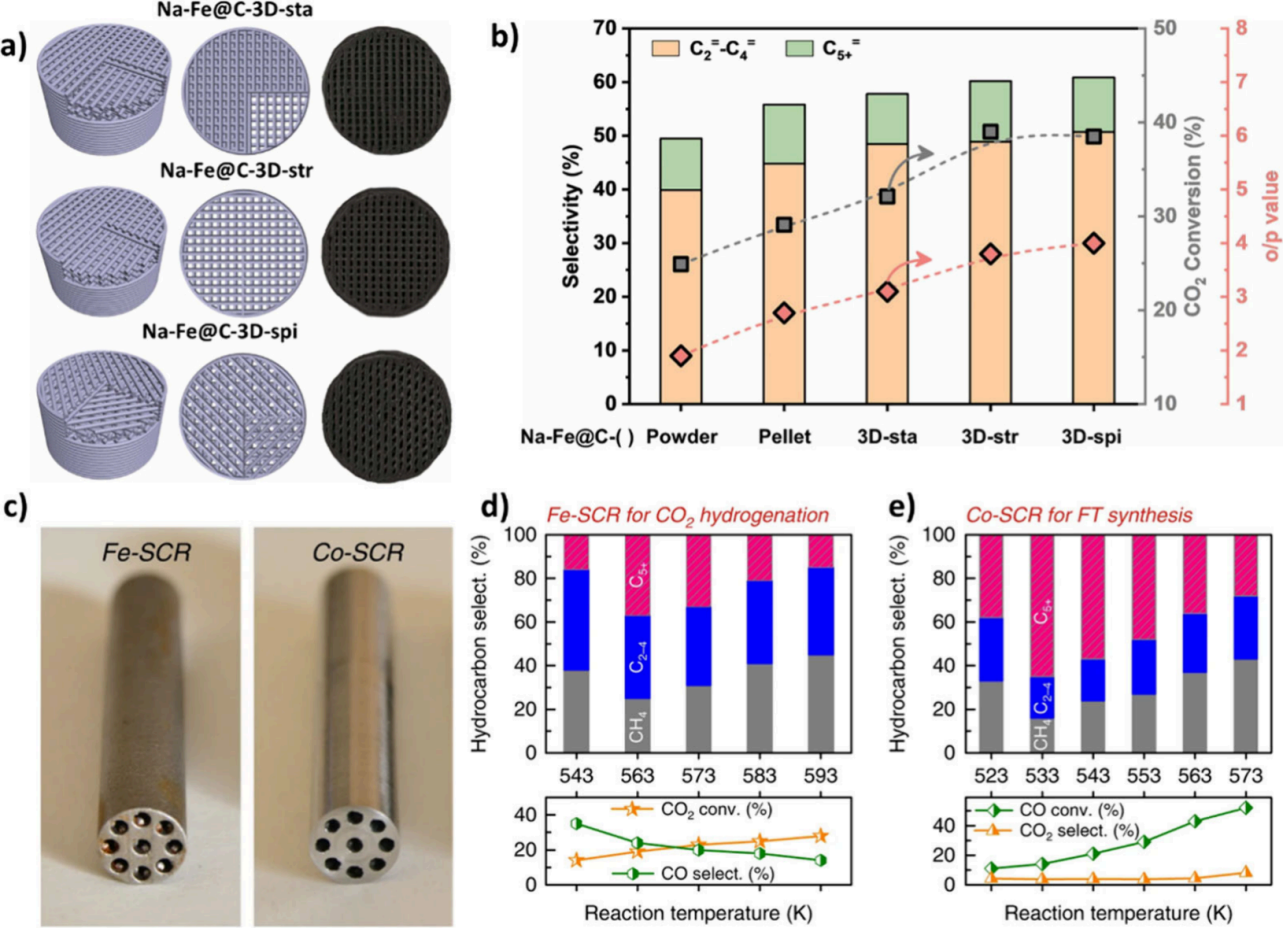
**a)** 3D printed monolithic Na-Fe@C catalysts and **b)** CO_2_ hydrogenation performance of non-3D printed and 3D printed Na-Fe@C catalysts (Reaction conditions: 320 °C, 3 MPa, 15 mL min^–1^). Reproduced with permission from Wang et al.^266^ Copyright 2024, Elsevier. **c)** The 3D printed SCR catalysts, **d)** CO_2_ hydrogenation performance of Fe-SCR at different temperatures (Reaction conditions: *P* = 1 MPa, 20 mL min^–1^), and **e)** FTS performance of Co-SCR at different temperatures (Reaction conditions: *P* = 2 MPa, 20 mL min^–1^). Reproduced with permission Wei et al.^273^ Copyright 2020, the Nature Springer Publishing under [CC BY 4.0] license.

#### 6.2.3 Supported Fe-Based Oxide

The distribution and proximity of active sites can be optimized by employing catalyst support materials with tailored pore structures or surface modifications.^274–276^ This level of control enables better management of catalytic performance and facilitates the formation of desired hydrocarbons in CO_2_ hydrogenation reactions.^277,278^

Furthermore, the pore size distribution of supports is also crucial in determining the catalytic performance. It can affect the reducibility, dispersion of metals, and mass transport. Results revealed that increasing the alumina pore size led to an increase in Fe_2_O_3_ particle size and, in turn, decreased iron dispersion. In addition, a small pore size resulted in a minimal particle size of Fe_2_O_3_, which is unfavorable for C–C bond growth. In this regard, Xie et al. showed that an appropriate pore size of approximately 7–10 nm for alumina could result in an optimum particle size of about 5–8 nm for Fe_2_O_3_ in FeK/Al_2_O_3_.^279^ However, Numpilai et al. showed that increasing catalyst pore sizes could increase the olefin/paraffin ratio due to the enhanced diffusion, suppressing the hydrogenation of readsorbed olefins. It was found that increasing the pore size of the γ-Al_2_O_3_ support from 6.2 to 49.7 nm increased the olefin/paraffin ratio from 3.93 to 6.38 in the K-Fe-Co/K-Al_2_O_3_ system.^167^

Liu et al.^280^ studied the effect of support on the CO_2_ hydrogenation performance of NaFe-supported catalysts. It was demonstrated that the surface carbon content increased in the order of SiO_2_ < Al_2_O_3_ < CNT < ZrO_2_. However, despite the highest olefin/paraffin ratio (8.05) and STY (111.4 g kg_cat_^–1^ h^–1^) in Na-Fe/ZrO_2_, the Na-Fe/Al_2_O_3_ showed the highest CO_2_ conversion of about 38.5% (Figure 30 (a) and (b)).^280^ In addition, Bao et al.^281^ showed that appropriate Fe-support interactions in Fe/Al_2_O_3_ and Fe/ZrO_2_ resulted in the formation of Fe_5_C_2_ and defect-rich FeOx, which could enhance olefin formation. In contrast, very poor or excessively strong interactions between Fe and support in Fe/TiO_2_ or Fe/CeO_2_ could not lead to the active components (Figure 30 (c)).^281^ Furthermore, it was verified that the introduction of Fe and K onto supports like Al_2_O_3_, SiO_2_, and ZrO_2_ can enhance the dispersion of active sites, consequently improving catalytic performance.^282^ However, only Al_2_O_3_ exhibits a strong interaction with K, forming KAlO_2_ that binds H firmly, impeding the hydrogenation of olefins to paraffins.^283,284^

**Figure 30:**
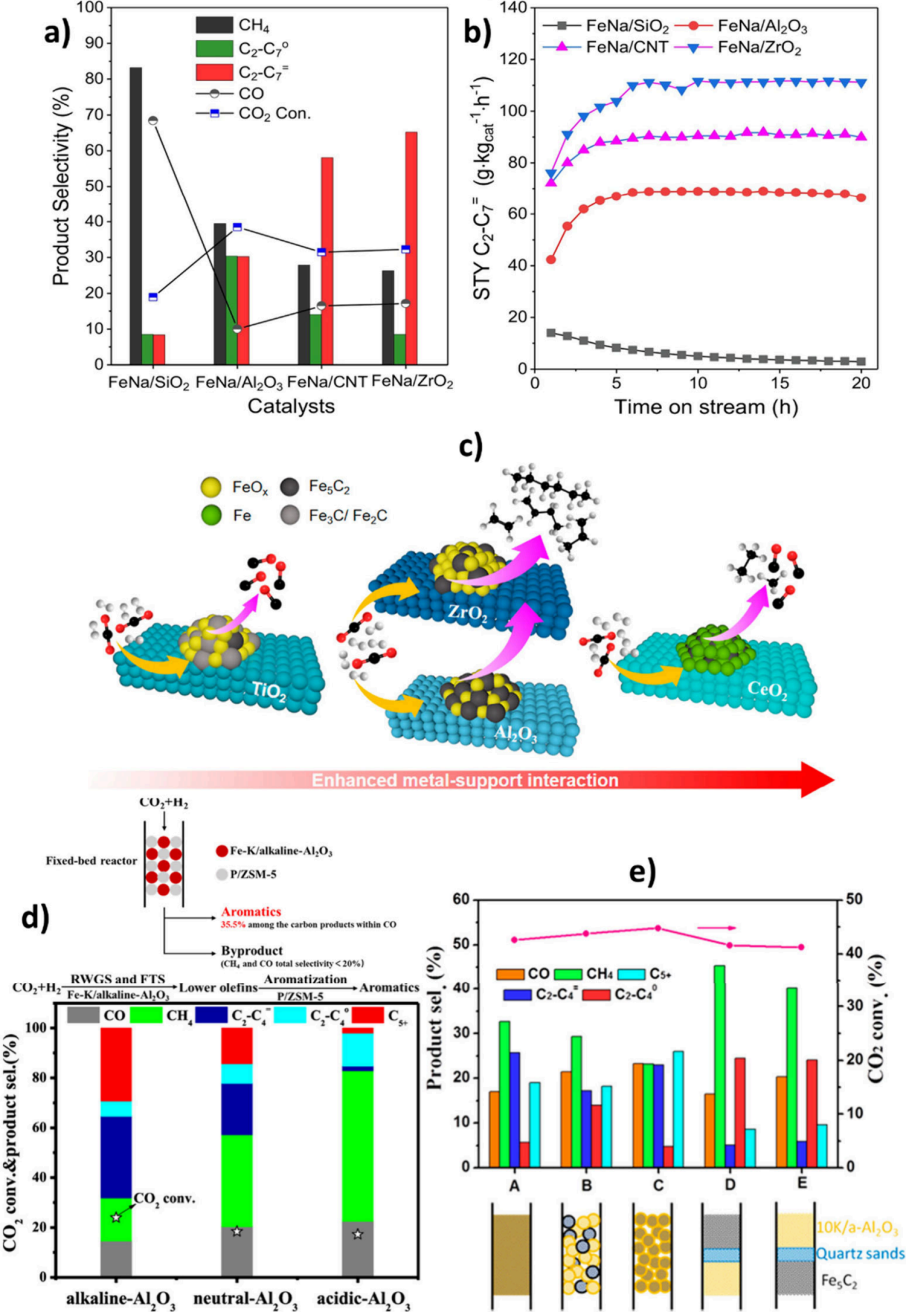
**a)** Catalytic performance and **b)** STY of C_2_–C_7_ olefins of supported Fe catalysts (Reaction conditions: 323 °C, 2 MPa, and 9000 mL g_cat_^–1^ h^–1^). Reproduced with permission from Liu et al.^280^ Copyright 2022, Elsevier. **c)** Schematic illustration of CO_2_ hydrogenation on different supported Fe Catalysts. Reproduced with permission from Bao et al.^281^ Copyright 2023 with permission obtained from ACS. **d)** Influences of various types of Al_2_O_3_ support over 15Fe-10K/Al_2_O_3_ catalysts on the CO_2_ conversion and product selectivity. Reaction conditions: 6000 mL g_cat_^–1^ h^–1^. Reproduced with permission from Dai et al.^212^ Copyright 2020 with permission obtained from ACS. **e)** The influence of different proximities of Fe_5_C_2_ and K/a-Al_2_O_3_ on CO_2_ hydrogenation performance (Reaction conditions: 3.0 MPa, 400 °C, and 3600 mL g^–1^ h^–1^). Reproduced with permission from Liu et al.^287^ Copyright permission obtained from ACS, 2018.

Alumina can slow down the reduction of iron oxide due to Fe-support interactions, thereby allowing for the FeC_*x*_/FeO_*x*_ ratio adjustment in the catalyst.^56^ Furthermore, Al_2_O_3_ is commonly utilized to enhance resistance against thermal sintering and physical attrition and improve the dispersion of Fe phases.^135^ The enhanced dispersion and reducibility of Fe_*x*_O_*y*_ species on the Al_2_O_3_ surface compared to bulk iron oxide may promote the creation of Fe sites, which readily form carbide during the reaction.^285^ Investigating the effect of alumina phases, Yu et al.^286^ showed that the more basic sites and less OH on FeNa/α-Al_2_O_3_ resulted in stronger CO_2_ activation and carburization, leading to more carbide formation and, in turn, higher olefin formation. In contrast, the presence of more OH content on the FeNa/γ-Al_2_O_3_ catalyst led to easier deactivation and formation of more CH_4_. Moreover, the hydrogen-rich environment resulted in overhydrogenation capacity and, therefore, a lower olefin/paraffin ratio.^286^

To provide a better insight into the role of Al_2_O_3_, Dai et al.^212^ investigated the influence of assembling different Al_2_O_3_ with K-promoted Fe_2_O_3_ and confirmed that different Al_2_O_3_ supports considerably influenced the product distribution at the same amount of the Fe and K impregnation (15 wt % Fe, 10 wt % K), as depicted in Figure 30 (d). The SEM and TEM analysis revealed that the alkaline Al_2_O_3_ (a-Al_2_O_3_) support exhibited a uniform distribution of small-sized Fe-K bimetallic particles. In contrast, the neutral and acidic Al_2_O_3_ supports showed evident particle agglomeration, leading to an irregular catalyst morphology. Additionally, the results demonstrated a robust interaction between Fe ions and a-Al_2_O_3_. This interaction could be attributed to the complexation of Fe ions and hydroxyl groups on the a-Al_2_O_3_ surface, resulting in a Fe species-rich surface with an abundance of electrons. Consequently, alkaline hydroxyl groups in a-Al_2_O_3_ enhanced the dispersion of Fe-K bimetal and increased CO_2_ adsorption. This hindered hydrogen adsorption, resulting in a lower H_2_/CO_2_ ratio on the catalyst surface, which favored the formation of intermediates. As a result, 15Fe-10K/a-Al_2_O_3_ catalyst was found to be highly selective in CO_2_ hydrogenation into light olefins and C_5+_ hydrocarbons.^212^

The promoter-metal proximity in the oxide phase plays a vital role in the performance of the catalyst, as confirmed by Liu et al.^287^ that exploited the K-promoted Al_2_O_3_, mixed it with Fe_5_C_2_ instead of promoting the bulk Fe-oxide, and integrated them in different proximity, as demonstrated in Figure 30 (e). Types A–C demonstrated lower CH_4_ selectivity compared to D.B. packed catalysts. Among them, type A showed higher selectivity toward light olefins (25.7%), while type C exhibited better C_5+_ selectivity (26.0%). However, the latter stacking approach (type C) displayed the highest overall selectivity for valuable C_2_–C_4_ olefins and C_5+_, along with the highest CO_2_ conversion rate of 44.8%. This suggests that close proximity, such as the mixed-powder granules (M.G.) mode, was crucial in achieving the highest C_5+_ yield.

### 6.3 Tuning the Oxide-Zeolite Proximity via Encapsulated Structures

The synergistic proximity effect in tandem reactions indicates that the two catalysts are coupled through a concentration gradient of the intermediate product.^288^ The encapsulated structure leads to exceptional catalytic and sorption properties, surpassing those of the individual core and shell materials in their pristine forms or even when physically mixed together. Encapsulated catalysts with hollow voids provide emerging alternatives to address significant challenges such as coking and sintering. Moreover, they offer further potential for facilitated diffusion and confinement effect to tune product distribution.^289,290^ This configuration creates a confined environment within the nanoscale dimensions of the core, allowing for unique chemical reactions or interactions to occur (Figure 31 (a)).^291–293^ These materials were categorized into core–shell, yolk–shell/hollow structures, and sandwiched core–shell structures, as depicted in Figure 31 (b), based on their structure and morphology.^235^ Gao et al.^294^ summarized synthesis methods of encapsulated materials and their catalytic performance. Despite their unique and intricate characteristics, core–shell catalysts can be viewed as more complex variants of common catalyst structures, such as supported metals, metal oxides, and alloys. Single or multicore–shell metal@metal oxide, metal oxide@metal oxide, and metal@carbon catalysts can be regarded as distinct instances of supported metals and metal oxides on different supports, where the interface with the support almost completely encloses the supported particles. Yolk@shell or yolk@hollow structures can be considered extensions of nanoparticles embedded within the channels of mesoporous supports. Metal@metal core–shells, on the other hand, represent a form of bimetallic nanoparticles with more pronounced spatial segregation of the individual metals compared to alloys.^235,292^ During the CO_2_ hydrogenation reaction on the core–shell catalyst, where the metallic core is encompassed by a zeolitic shell, the reactants initially pass across the shell to reach the core catalyst, where linear intermediates are formed. Subsequently, all hydrocarbons must pass through the zeolite shell before leaving the catalyst, and the heavier hydrocarbons have more opportunities to undergo conversion into long-chain hydrocarbons on the acidic sites of the zeolite shell.^295^ This leads to a higher selectivity for light iso-paraffins in the final products. These findings have inspired numerous researchers to adjust the proximity effect in heterogeneous catalysis by designing core–shell catalytic systems where the metal oxide forms the core and the zeolite forms the shell.^296–298^ Moreover, by tuning the synthesis method, bimetal-loaded dual-layer structures, i.e., M1@hsZSM5@M2, can be prepared to take advantage of the properties of two metals without direct contact, as depicted in Figure 31 (c).^299^ In this context, Wang et al.^296,300^ investigated the effect of the assembly of Fe-Zn-Zr and HZSM-5 on the CO_2_ hydrogenation performance. In all configurations except powder mixing, C_5+_ nonaromatics account for the higher proportion of the total C_5+_ gasoline. It was revealed that, by separating active sites via a dual-bed configuration, a high amount of CO (66.3%) was produced. In contrast, by increasing the proximity of Fe-Zn-Zr and HZSM-5 through granule mixing, the CO selectivity considerably decreased, and more C_2_–C_4_ hydrocarbons formed. This can be ascribed to the repeated contact of metallic and zeolitic active sites, as well as the further hydrogenation of products to saturated hydrocarbons. By exploiting the core–shell structure (Figure 31 (d)), the highest proportion of C_5+_ nonaromatics (around 91.9%) in total C_5+_ gasoline at a 21.5% CO_2_ conversion was achieved. It was attributed to the basic-silica modification of zeolitic Brønsted acid sites, which inhibited further hydrogenation, aromatization, and cyclization. Also, by further increasing the proximity through the powder mixing configuration, which provides the closest proximity between active sites, more C_5+_ gasoline was produced compared to the core–shell structure. At the same time, aromatics account for a higher proportion of C_5+_ gasoline, ∼50.3%, as shown in Figure 31 (e).

**Figure 31:**
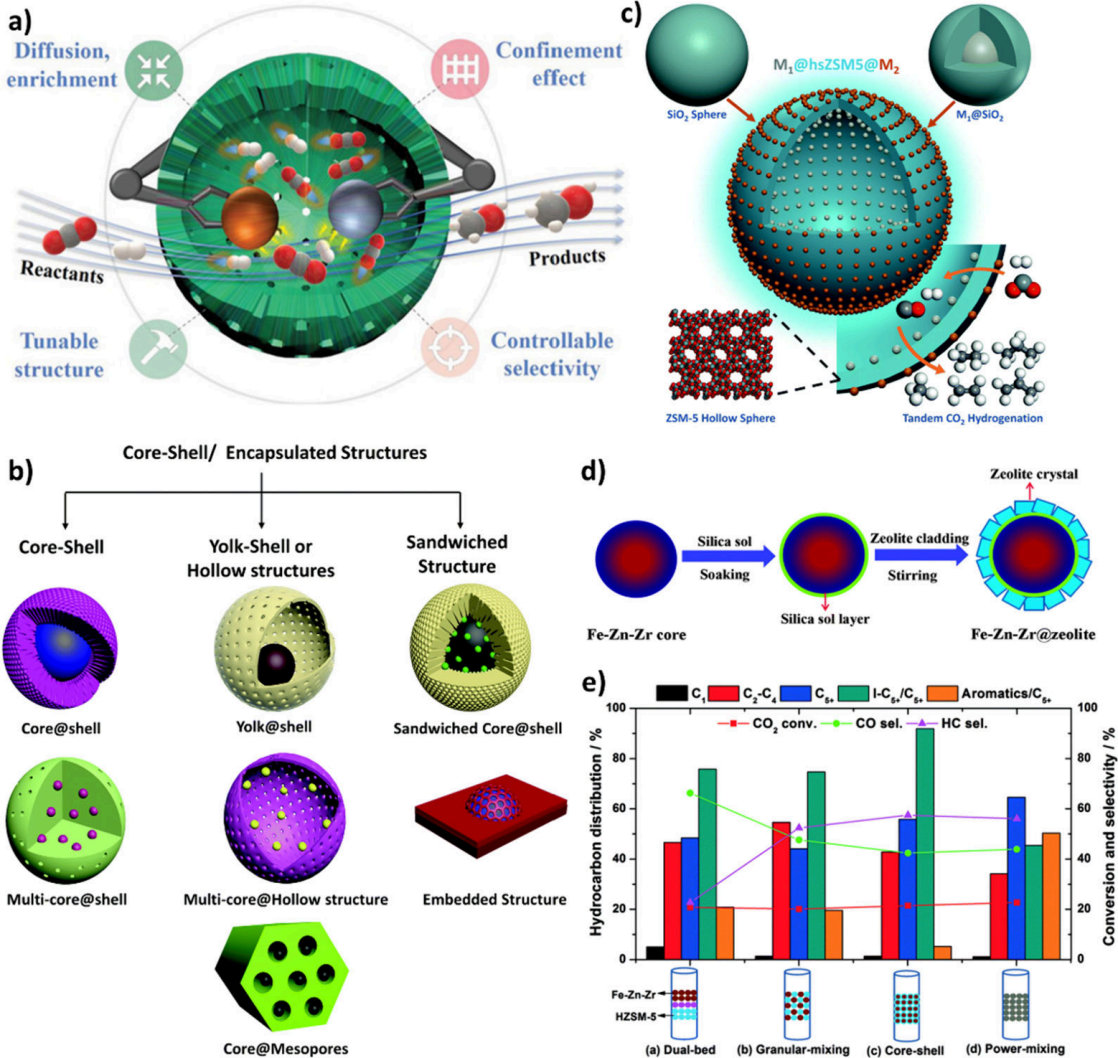
**a)** Illustration of the benefits of encapsulated structures in a schematic diagram. Reproduced with permission from Ye et al.^291^ Copyright 2021, Wiley Publishing under [CC BY 4.0] license. **b)** Schematic depicting various core–shell structures based on morphology. Reproduced with permission from Das et al.^235^ Copyright 2020, RSC under [CC BY-NC 3.0]. **c)** Schematic illustration of dual-layer metal-supported zeolite M1@hsZSM5@M2. Reproduced with permission from Kwok et al.^299^ Copyright 2020, RSC. **d)** Schematic illustrating the preparation of the Fe-Zn-Zr@zeolite core–shell tandem catalyst using a straightforward cladding technique. Reproduced with permission from Wang et al.^296^ Copyright permission obtained from RSC, 2016. **e)** Effect of different integration manners of Fe-Zn-Zr and HZSM-5 on the CO_2_ hydrogenation performance (Reaction conditions: 340 °C, 5.0 MPa, 3000 mL g^–1^ h^–1^, Fe-Zn-Zr:HZSM-5 = 4:1 (weight ratio), SiO_2_/Al_2_O_3_ = 50. Reproduced with permission from Wang et al.^300^ Copyright permission obtained from RSC 2019.

Moreover, it was shown that modifying the core structure by adding TPABr reduced CO formation. In addition, more C_5+_ nonaromatics were formed via Fe-Zn-Zr-T@HZSM-5 core–shell compared to granule stacking manner (Figure 31 (e)).^300^ It is worth mentioning that, compared to alkali-promoted Fe-based catalysts, Fe-Zn-Zr exhibits higher selectivity toward oxygenates, indicating that the methanol-mediated pathway is the dominant route compared to FTS. In addition, after combining with zeolites, nonaromatics production was higher than that of aromatics.^301^

### 6.4 Effect of Oxide-Zeolite Integration Method

Overall, the proximity of active sites in Fe-based catalysts for CO_2_ hydrogenation plays a crucial role in promoting the formation of C_5+_ hydrocarbons. It influences diffusion limitations, affects the adsorption and reactions of intermediates, enables complex surface reactions, determines the catalytic pathways, and can be tailored through catalyst design for improved selectivity toward higher hydrocarbons. Many parameters should be taken into consideration while selecting the best oxide/zeolite arrangement, such as (i) the nature and loading of the alkali promoter, (ii) whether the promoter is loaded on the metal oxide, support, or zeolite, (iii) the method of promoter loading (impregnation, sol–gel, or physical mixing), (iv) the acidity and Si/Al ratio of the zeolite, (v) different treatments to modify BAS, and (vi) the weight ratio of metal oxide to ZSM-5. Considering these effects and based on the explanations provided in sections 4.1, the promoted Fe-based oxide integrated with zeolite are classified based on their alkali promoters.

#### 6.4.1 Na-Promoted Fe-Based Oxide and Zeolite

Na can act as a promoter in Fe-based CO_2_ hydrogenation catalysts by facilitating CO_2_ activation and promoting the generation of reactive species, particularly carbonate-like intermediates, and subsequent dissociation of CO, which is essential for the hydrogenation reaction. Moreover, Na interacts with the catalyst surface, leading to the formation of active sites via modifying the catalyst’s electronic properties.^302–304^ These effects collectively contribute to the enhanced catalytic activity of Na-promoted Fe-based catalysts in CO_2_ hydrogenation.^305^ It was uncovered that Na can migrate to the surface of Fe-based oxide catalysts and then move toward acid sites of zeolite, depending on their respective distances. This migration can impact the functionality of both basic and acidic sites, thus influencing the overall catalytic performance of the catalyst.^306,307^ Therefore, providing appropriate proximity between Na-promoted Fe-based particles and zeolite is of utmost significance in obtaining the desired products. For instance, it was shown that by integrating 0.7%Na-Fe_3_O_4_ and HZSM-5(160) particles via M.G., which provided the smallest distance between active sites, CH_4_ was the main product (60% selectivity) at low CO_2_ conversion (13%). This was attributed to the reduced basicity of the Fe_3_O_4_ surface and, in turn, its carburization extent due to the poisoning of alkali sites by the acidity of the zeolite at close proximity. The catalyst prepared via incipient wetness impregnation (2%Na-10%Fe/HZSM-5) similarly presented poor CO_2_ activity (about 5.4% conversion) while exhibiting CO selectivity of ∼29.5%. However, combining particles through granule stacking (G.S) increased the spatial distance between active sites. Accordingly, olefin intermediates formed over Na-Fe_3_O_4_ were transferred to HZSM-5 and converted mainly to C_5+_ at a high CO_2_ conversion rate (34%). Further increasing the spatial distance between active sites via D.B. integration led to lower selectivity toward C_5+_ while CO_2_ conversion remained unchanged, as depicted in Figure 32 (a).^201^ Moreover, it was illustrated that the composition of gasoline C_5+_ hydrocarbons depended on the proximity of active sites. Although the content of aromatics in total C_5+_ hydrocarbons was higher than that of nonaromatics under both G.S. and D.B. integration, more nonaromatics were formed in the latter configuration.^201,308^ It was speculated that higher H_2_ partial pressure under a D.B. configuration resulted in more hydrogenation rather than aromatization reactions and thus the formation of more nonaromatics (Figure 32 (b)).^308^ A similar trend was observed by Wen et al.,^309^ who studied the effect of different proximities of 1.11%Na-Fe_3_O_4_ and HZSM-5(30) on CO_2_ hydrogenation performance. It was demonstrated that mortar mixing of powders reduced the formation of active carbides under FTS reaction due to the detrimental effect of zeolite acidity on the basic sites of iron-oxide induced by Na. Therefore, in the closest proximity of the active sites, about 97.9% of the CO was formed at poor CO_2_ hydrogenation. Increasing the proximity via G.S. and D.B. configurations considerably increased CO_2_ hydrogenation performance, and the highest C_5+_ selectivity (66.8%) at 26.5% CO_2_ conversion was achieved under the D.B. mode.^309^ Moreover, Wang et al.^45^ achieved the lowest CO_2_ conversion (15.5%) and the highest CH_4_ selectivity (49%) on mixed-powder granules of 0.47%Na-Fe@C and HZSM-5(40)-0.2 M (NaOH treated). Increasing the spatial distance via G.S. increased CO_2_ conversion and aromatic selectivity (50.2%). In this mode, the continuous conversion of alkenes to aromatics on zeolitic acid sites could shift the RWGS equilibrium forward, resulting in increased CO_2_ conversion (33.3%). Further increasing the distance by using two separate reactors in series reduced CO_2_ conversion and aromatic selectivity, while nonaromatic selectivity reached its highest value (39%), indicating higher hydrogenation of intermediates. Amoo et al.^206^ also showed that G.S. integration of Na-Fe@C with HZSM-22 and HZSM-5 was more favorable compared to D.B. that resulted in 60.8% and 59.3% C_5+_ selectivity, respectively.

**Figure 32:**
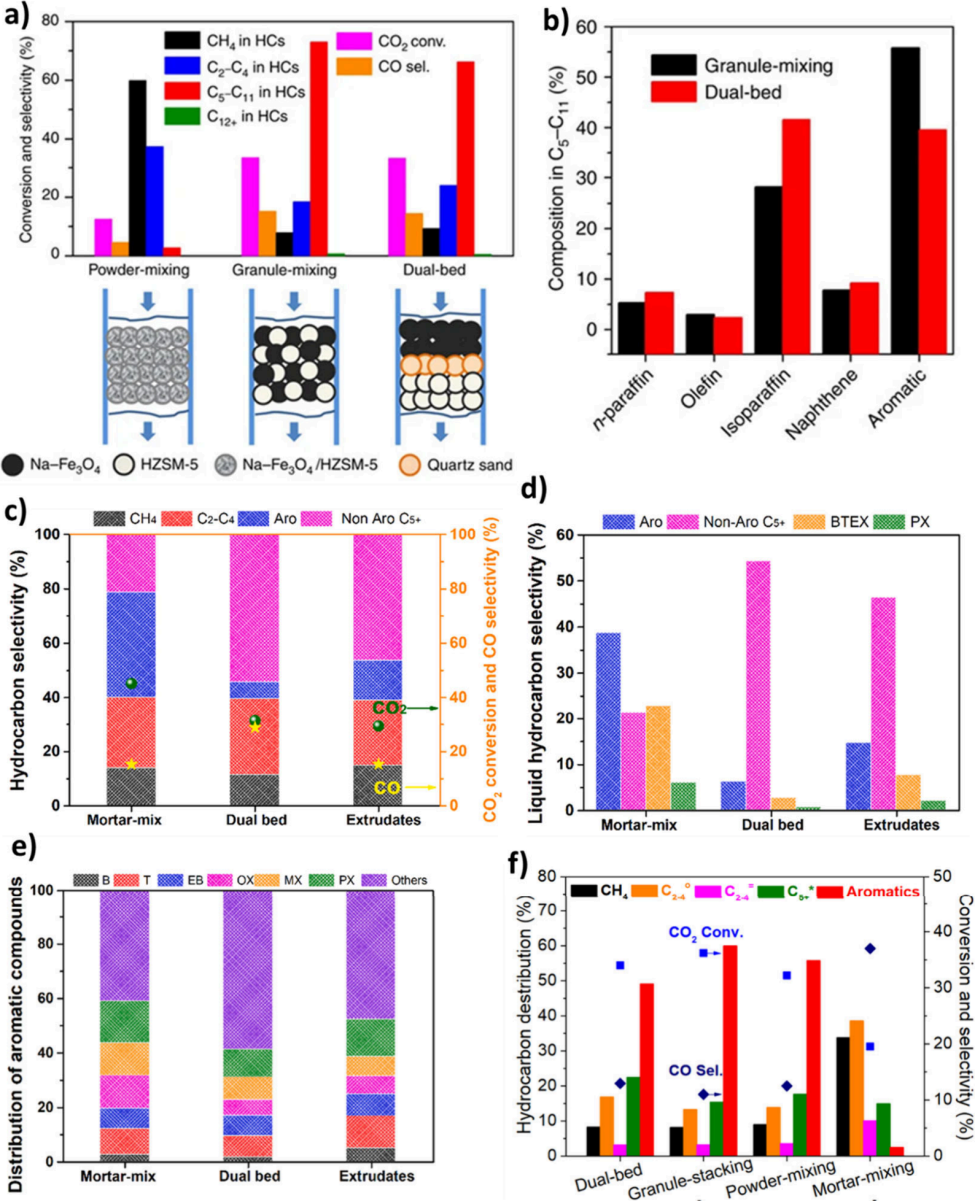
**a)** The influence of different Na-Fe_3_O_4_ and HZSM-5(160) combinations on the CO_2_ hydrogenation performance, **b)** comparison of the gasoline composition in two different catalyst integration manners. Reproduced with permission from Wei et al.^201^ Copyright 2017, Nature Springer Publishing under [CC BY 4.0] license. The effect of the integration manner of 0.74%Na-FeAlOx and Zn-HZSM-5(12.5)@SiO_2_ on **c)** CO_2_ conversion and product selectivity, and **d)** liquid product distribution. **e)** Aromatic distribution. Reaction conditions: H_2_/CO_2_ = 1:3; 3.5 MPa; and 4000 mL g^–1^ h^–1^ (Note: B (benzene), Aro (aromatics), EB (ethylbenzene), Non-Aro C_5+_ (nonaromatic C_5+_), T (toluene), X (xylene), PX (p-xylene), OX (o-xylene), MX (m-xylene)). Reproduced with permission from Sibi et al.^216^ Copyright 2022, Elsevier. **f)** The influence of different proximities between ZnFeO_*x*–4.25_Na (Fe:Zn = 3:1) and S-HZSM-5(25) on CO_2_ hydrogenation performance. Reproduced with permission from Cui et al.^210^ Copyright 2019, ACS. Reaction conditions: 3.0 MPa, 320 °C, and 4000 mL g_cat_^–1^ h^–1^ (Note: C_5+_* refers to C_5+_ products except for aromatics).

Incorporating Al into magnetite via the coprecipitation method could change the concentration of oxygen vacancies and carburization ability that influences the RWGS and FTS reactions, respectively.^310,311^ Moreover, it has been revealed that more CH_*x*_ intermediates were formed over Na-FeAlO_*x*_ than those produced over Na-Fe_3_O_4_. The presence of the amorphous AlO_*x*_ phase increased the readsorption of light olefins, which are vital for the formation of heavier hydrocarbons.^312^ However, the dispersion of Na and Fe over the AlO_*x*_ lattice may increase the distance between basic and acidic sites. Therefore, a closer proximity was required for this catalyst to provide the best C_5+_ yield than Na-Fe_3_O_4_ and ZSM-5. Moreover, coating the ZSM-5 surface BAS might also necessitate the closer proximity of the materials to facilitate the access of intermediates to the zeolite pores. This was also confirmed by Sibi et al.,^216^ that mixed-powder granules with the closest proximity of 0.74Na-FeAlO_*x*_ and Zn-HZSM-5(12.5)@SiO_2_ provided the best performance in terms of both CO_2_ conversion (45.2%) and aromatic selectivity (38.7%), as can be observed in Figure 32 (c).^216^ It can be observed that as the distance increased, the nonaromatic proportion increased in the C_5+_ fraction (Figure 32 (d)), while the distribution of aromatics remained almost the same (Figure 32 (e)).^216^ Notably, the coverage of the pore opening and surface BAS of the ZSM-5 by SiO_2_ necessitates a temperature increase of up to 370 °C to facilitate the diffusion of intermediates into the zeolite pores.^312^ Based on the CO_2_-TPD analysis, the active sites integrated by G.S. exhibited a reduced ability to adsorb CO_2_ compared to the ones integrated by mortar mixing, indicating that at closer proximity, new interfacial sites were created, facilitating CO_2_ adsorption.^216^

Zn has been extensively utilized as a promoter in conjunction with alkali metals for Fe-based metal oxides in CO_2_ hydrogenation, as described in section 4.2.1. Moreover, the coexistence of Zn and Na was effective in hindering the oxidation of Fe_5_C_2_ to FeO_*x*_ and inhibiting the catalyst deactivation.^128,132^ In this regard, Cui et al.^210^ studied the influence of Na-promoted spinel ZnFe_2_O_4_ (Fe:Zn = 3:1) and HZSM-5(25) integration method on CO_2_ hydrogenation performance. It was demonstrated that while the metal oxide and zeolite particles were in the closest proximity (via mortar mixing), only 2.5% selectivity to aromatics was achieved at 19.5% CO_2_ hydrogenation (Figure 32 (f)). However, increasing the distance between Na-induced alkali sites of iron oxide and Brønsted acidity of zeolite via G.S. improved the performance regarding both CO_2_ hydrogenation (36%) and aromatic selectivity (60%). Further increasing the distance between active sites to D.B. resulted in lower aromatic selectivity, while the selectivity of the nonaromatic proportion of C_5+_ hydrocarbons increased. It was also observed that the 2p_3/2_ XPS peak of Fe_5_C_2_ shifted to lower binding energies for the Na-promoted Zn-FeO_*x*_ compared to the nonpromoted Zn-FeO_*x*_, indicating the formation of electron-rich Fe_5_C_2_, which inhibited the hydrogenation of olefins to paraffins.^210^ The same phenomenon was observed by Jiang et al.,^313^ which showed that the G.S. mode provided the optimum distance for combining NaZnFe13 and S-HZSM-5-0.5 to produce higher C_5+_ aromatics, as depicted in Figure 33 (a). However, increasing the distance to D.B. mode resulted in the formation of more C_2_–C_4_ paraffins (22.37%), confirming higher hydrogenation possibilities in this mode. In contrast, Ra et al.^189^ showed that both the D.B. and G.S. modes of Na/ZnFe_2_O_4_ and ZSM-5(40) exhibited high CO_2_ conversion to C_5+_ hydrocarbons. However, when using powder and mortar mixing methods, the production of CO and CH_4_ was more prevalent (Figure 33 (b) and (c)). Additionally, maintaining an appropriate proximity in the D.B. integration mode could hinder Na migration and prevent acid site deactivation.

**Figure 33:**
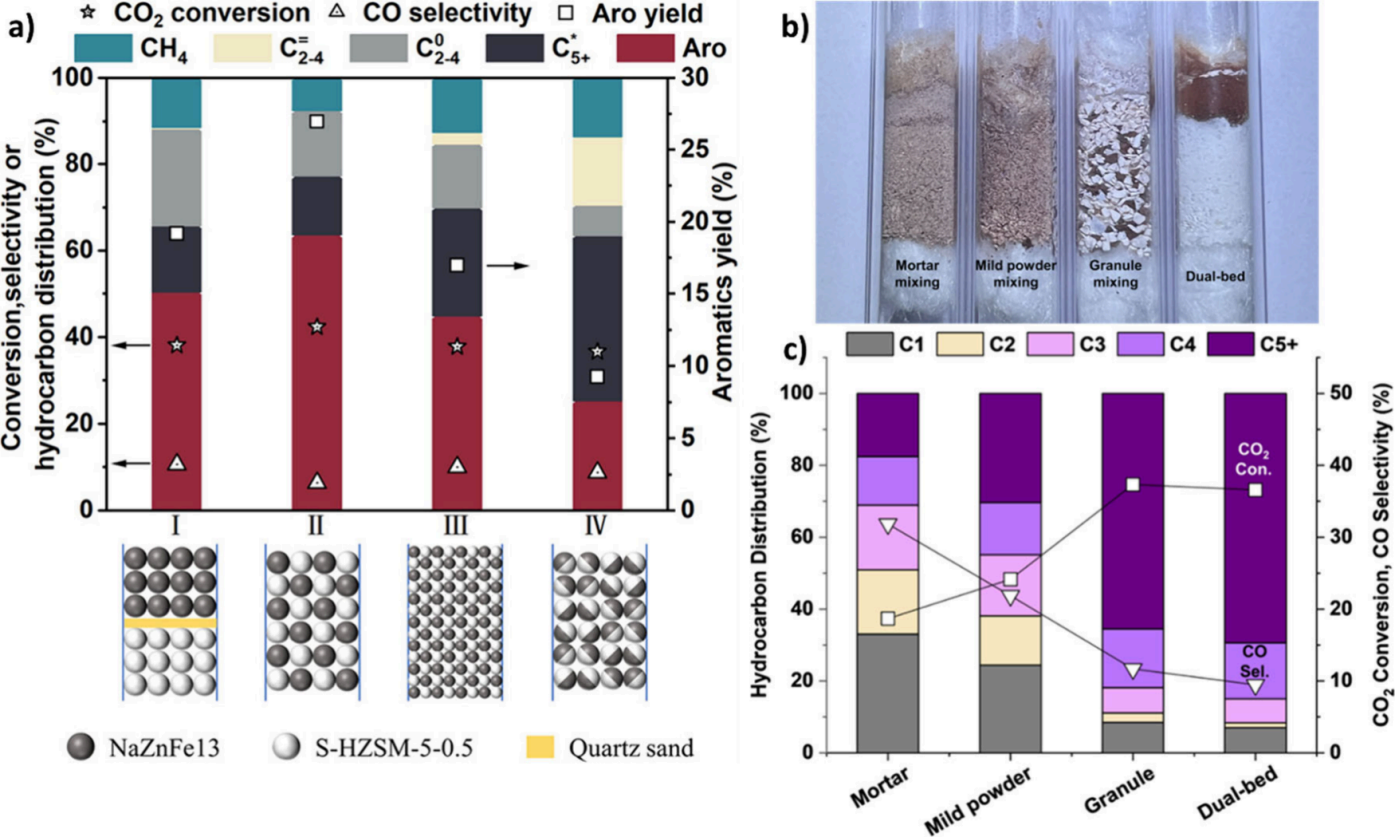
**a)** The effect of the integration manner of NaZnFe13 and S-HZSM-5-0.5 on the performance of CO_2_ hydrogenation. Reproduced with permission from Jiang et al.^313^ Copyright 2023, ACS. Reaction conditions: 3.0 MPa, 320 °C, and 1000 mL g^–1^ h^–1^ (Note: I: dual bed (40–60 mesh); II: granule mixing (40–60 mesh); III: powder mixing (200–300 mesh); and IV: powder mixing and then pressing into 40–60 mesh). **b)** Schematic of different integration manners of Na/ZnFe_2_O_4_ and ZSM-5(40). **c)** The influence of the stacking manner of Na/ZnFe_2_O_4_ and ZSM-5(40) on CO_2_ hydrogenation performance (Reaction conditions: *P* = 2.0 MPa, *T* = 340 °C, and 2700 mL g_cat_^–1^ h^–1^). Reproduced with permission from Ra et al.^189^ Copyright 2023, Elsevier.

Mn is another important transition metal, which has been used in combination with Na-promoted iron oxide, as described in section 4.2.3. The coexistence of Na with Mn can boost the distinctive synergy that can suppress the negative effects on RWGS caused by close Fe-Mn contact, thus sustainably enhancing the efficiency of CO_2_ hydrogenation to lower olefins, which are the key intermediates for higher hydrocarbons. In light of this achievement, it is hypothesized that the composite Fe-Mn-Na catalyst can also stimulate excellent catalytic performance for C_5+_ synthesis. Despite the beneficial synergy, the integration manner between Na and Mn and how this synergy affects CO_2_ hydrogenation are still active areas of research.^157,163^ To elucidate this synergy, Song et al.^157^ investigated the proximity of metallic sites by changing the synthesis method of Na-Fe-MnO_*x*_ (Figure 34 (a)). Accordingly, five catalysts were prepared via (I) coprecipitation, (II) impregnation of Fe_2_O_3_ powder by Na and Mn, (III) impregnation of FeMnO_*x*_ powder by Na, (IV) impregnation of FeMnO_*x*_ granules by Na, and (V) impregnation of Si-wrapped FeMnO_*x*_ granules by Na. It was observed that the worst performance belonged to cases (I) and (V), where the active sites were in the closest and farthest proximities, respectively, while case (III) exhibited the best performance in terms of CO_2_ conversion, along with CH_4_ and aromatic selectivity. This was explained by HRTEM images of the spent samples, which showed that in case (I), two kinds of lattice fringes of Fe_3_O_4_ (311) and Fe_5_C_2_ (510) were found on the same diffraction facet, implying the aggregation and shrinkage of the active sites. However, in case (V), Fe_5_C_2_ and Fe_3_O_4_ were at their farthest distance, which was detrimental to the efficient diffusion of intermediates. In case (II), the active sites were isolated, while in cases (III) and (IV), some overlapping active sites were found; nonetheless, the number of nonoverlapped active phases was still higher, which could facilitate the diffusion of both reactants and intermediates. In addition, XPS studies showed that the binding energies of Fe 2p doublet in case (III) were lower than those of other cases, which indicated a higher charge transfer from Na to the surface of Fe species and, in turn, increased basicity that led to higher CO_2_ adsorption.^157^ In another study, Gao et al.^156^ studied the influence of the proximity of 2.83Na-Fe(90)­Mn(10) and HZSM-5@S1-S on product distribution, especially the PX/X ratio. It was revealed that the passivation of ZSM-5 acidity via silicalite in the core–shell structure could limit the isomerization reaction of PX and, hence, increase the PX/X ratio. Moreover, the G.S mode was found to be the most beneficial stacking scheme (Figure 34 (b)) for the formation of more aromatics (17%) and a higher PX/X ratio (75.4%) due to the most efficient diffusion of light olefin intermediates through the zeolite pores.^156^

**Figure 34:**
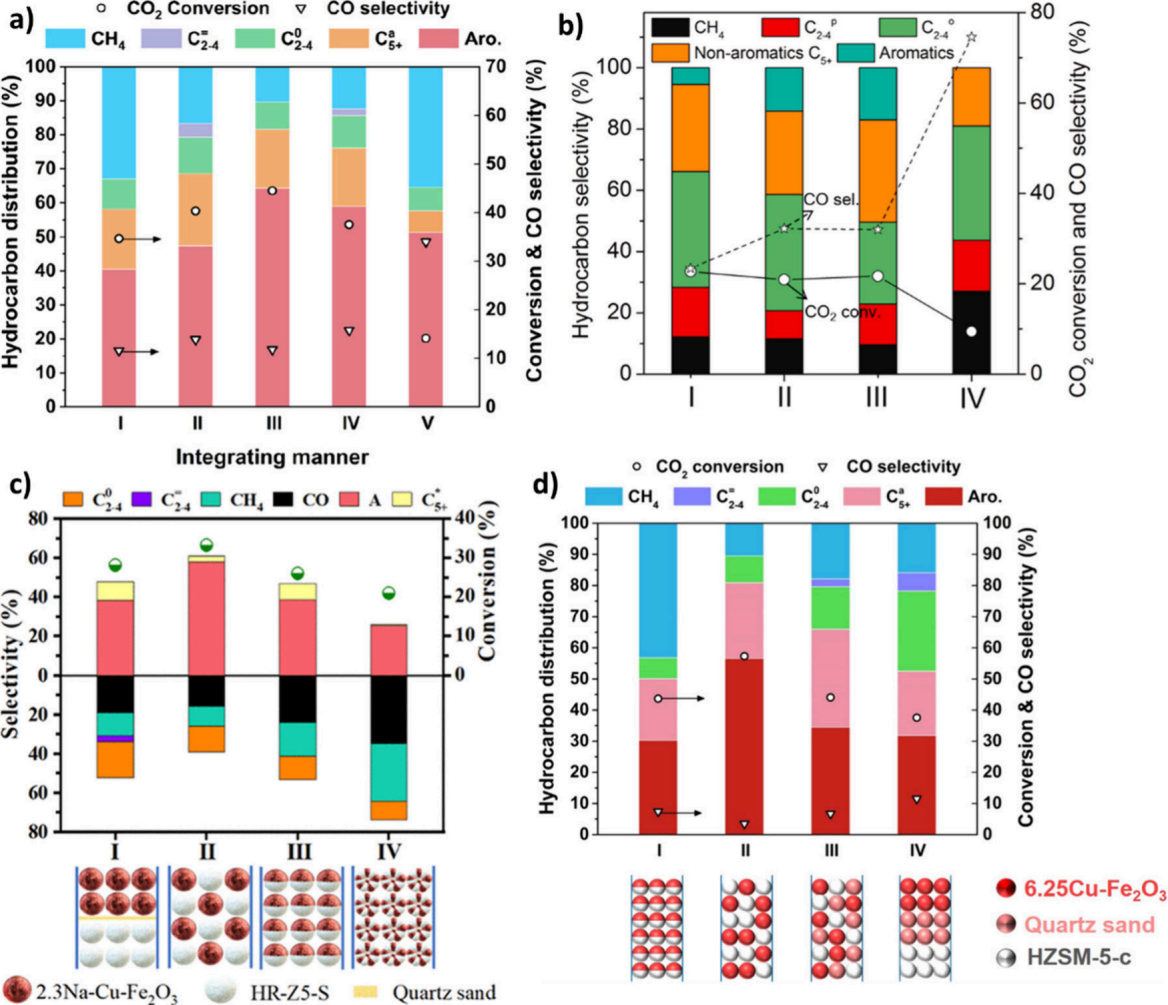
**a)** Catalytic performance of Na-Fe-MnOx integrated in different manners (I to V). Reproduced with permission from Song et al.^157^ Copyright permission obtained from ACS, 2022. Reaction conditions: 3.0 MPa, 320 °C, and 1000 mL g^–1^ h^–1^ (Notes: C_5+_^a^ refers to C_5+_ aliphatic). **b)** CO_2_ hydrogenation performance of 2.83Na-FeMn (90/10) integrated with HZSM-5@S1–S. Reaction conditions: 3.0 MPa, 4000 mL g_cat_^–1^ h^–1^, 320 °C. Reproduced with permission from Gao et al.^156^ Copyright permission obtained from Elsevier, 2022. **c)** The effect of the integration manner of 2.3Na-Cu-Fe_2_O_3_ and HR-Z5-S tandem catalysts on the hydrogenation of CO_2_ performance (Note: I: dual bed (40–60 mesh); II: granule mixing (40–60 mesh); III: powder mixing (200–300 mesh); and IV: powder mixing and then pressing to 40–60 mesh). Reaction conditions: 3.0 MPa, 320 °C, and 1000 mL g^–1^ h^–1^. Reproduced with permission from Yang et al.^142^ Copyright permission obtained from ACS, 2022. **d)** The influence of stacking method of 6.25Cu-Fe_2_O_3_ and HZSM-5-c tandem catalysts on the CO_2_ hydrogenation performance (Note: I: powder mixing, II: granule mixing, III: granule mixing with quartz sand, and IV: dual bed). Reaction conditions: H_2_/CO_2_/N_2_ = 72/24/4, 3.0 MPa, 320 °C, and 1000 mL g^–1^ h^–1^. Reproduced with permission from Song et al.^140^ Copyright permission obtained from ACS, 2020.

Cu is another recognized promoter that enhances the reducibility of ferric oxide and favors subsequent carburization of Fe species in Fe-based catalysts, as explained in section 4.2.2. CuFeO_2_ was considered an effective catalyst for demonstrating activity in producing liquid fuel from direct CO_2_ hydrogenation within a single reactor, resembling a conventional CO-FT catalyst for heavy hydrocarbon production.^143^ In order to unravel the influence of proximity in Na-promoted Cu-Fe catalysts, Yang et al.^142^ integrated 2.3% Na-promoted Cu-Fe_2_O_3_ (Fe/Cu molar ratio = 15) with HZSM-5(25) at different distances. Results revealed that the lowest performance that led to the highest amount of CH_4_ and CO was obtained in the closest proximity (powder mixing) due to the mutual poisoning of basic and acidic active sites, while with increasing the distance to G.S., the largest amount of aromatics (around 57.7%) at 33.3% CO_2_ conversion was achieved. Further increasing the distance via D.B. mode resulted in some unconverted C_2_–C_4_ olefins and thus reduced selectivity of aromatics (38.34%) (Figure 34 (c)), indicating the weakened synergy between the active sites. Moreover, Song et al.^140^ showed similar trends for 6.25% Cu-Fe_2_O_3_ and HZSM-5(25) and revealed that the G.S. integration manner led to the lowest CH_4_ and the highest C_5+_ hydrocarbons, as shown in Figure 34 (d).

In summary, in almost all cases when Na-Fe-based oxide and ZSM-5 were used as catalysts, the higher proportion of C_5+_ belongs to the aromatics due to the stronger BAS of ZSM-5 compared to other zeolites like HMCM-22 and H-beta.^314,315^ However, different integration schemes of Fe-based oxide and ZSM-5 could alter the C_5+_ distribution.^15^ Moreover, it can be speculated that generally (this trend commonly observed, though not universal), over Na-Fe and ZSM-5 in the G.S. scheme, hydrogen consumption via RWGS and FT reactions results in a lower hydrogen partial pressure over ZSM-5, which is favorable for aromatization reactions. Besides, since the active sites of metal oxides are in appropriate proximity to those of zeolites for C–C coupling reactions, the produced light hydrocarbons (intermediates) can diffuse quickly through zeolite pores. However, in the D.B. mode, the intermediates easily undergo hydrogenation reactions over active metal oxide sites before entering the remotely located zeolite, which favors the light olefins hydrogenation reactions.^308^ In addition, the partial pressure of hydrogen in the D.B. mode seems to be higher over zeolite, which can facilitate hydrogenation and increase the nonaromatic proportion of C_5+_. This trend can be observed in most of Na-promoted Fe-based catalysts integrated with ZSM-5 mentioned above, demonstrating that the selectivity of the nonaromatic proportion of C_5+_ increased via D.B. integration, while aromatic selectivity increased in the G.S. mode. It was also observed that the G.S. mode provides a larger Aro/non-Aro ratio than the D.B. mode. However, Wen et al.^309^ and Noreen et al.^202^ demonstrated that the D.B. mode was the best configuration for both nonaromatic and aromatic selectivity for combining Na-Fe and ZSM-5, which can be attributed to the high Brønsted acidity of the ZSM-5 used. Therefore, at closer distances, poisoning of metallic basic sites and strong zeolite acidic sites might be a possible cause of low efficiency. Nevertheless, M.G. was considered the best configuration for Na-promoted Fe-based catalysts, which have been dispersed over or mixed with other phases like amorphous AlO_*x*_, regarding total C_5+_ selectivity and yield.^216^

#### 6.4.2 K-Promoted Fe-Based Oxide and Zeolite

K serves as an electronic promoter in Fe-based CO_2_ hydrogenation catalysts, impacting their catalytic performance by adjusting both conversion and product distribution. It plays a crucial role in enhancing the adsorption of CO_2_ molecules and facilitating the generation of critical active intermediates, which are fundamental for the desired hydrogenation reactions.^316,317^ Through its role as a promoter, K alters the surface characteristics of Fe-based catalysts, impacts selectivity, and enhances the stability of catalysts during CO_2_ hydrogenation reactions. These effects collectively enhance the overall performance and efficiency of the catalyst system.^318–320^ Studies have shown that high loadings of K are essential to activate CO_2_ through the potassium carbonate (KCO_3_) mechanism and convert it to CO through potassium bicarbonate/formate interconversion, in addition to the dominant electronic promotional mechanism.^321^ More recently, it was shown that the grinding-mixed bifunctional catalyst exhibited reduced CO_2_ conversion and a significantly different product distribution when compared to the G.S. and D.B. catalysts. The structural analysis indicated the migration of K from the K-promoted Fe-based component to the ZSM-5 and the strong interaction between Fe and the zeolite at a close distance. These two factors could result in electron deficiency on the Fe surface, hindering the activation of CO_2_. Additionally, the reduction and carburization of FeO_*x*_ species were restricted, leading to the inhibition of FeC_*x*_ active site formation.^322^ Dai et al.^212^ investigated the influence of proximity between Fe-K/a-Al_2_O_3_ and HZSM-5(25) on CO_2_ hydrogenation performance and showed that by increasing the distance, C_5+_ distribution shifted from more iso-hydrocarbons to aromatics. Therefore, close proximity is required to achieve the desired product distribution, as can be observed in Figure 35 (a).^212^ This is linked to the nature of Al_2_O_3,_ which led to the dispersion of K and Fe in remotely located sites. In addition, the H_2_/CO_2_ ratio over Fe-K/Al_2_O_3_ was 1, which can be another reason for the closer proximity required. Therefore, the production of iso-hydrocarbons required closer proximity, but the nature of the support and feed ratio determined the extent of the proximity.

**Figure 35:**
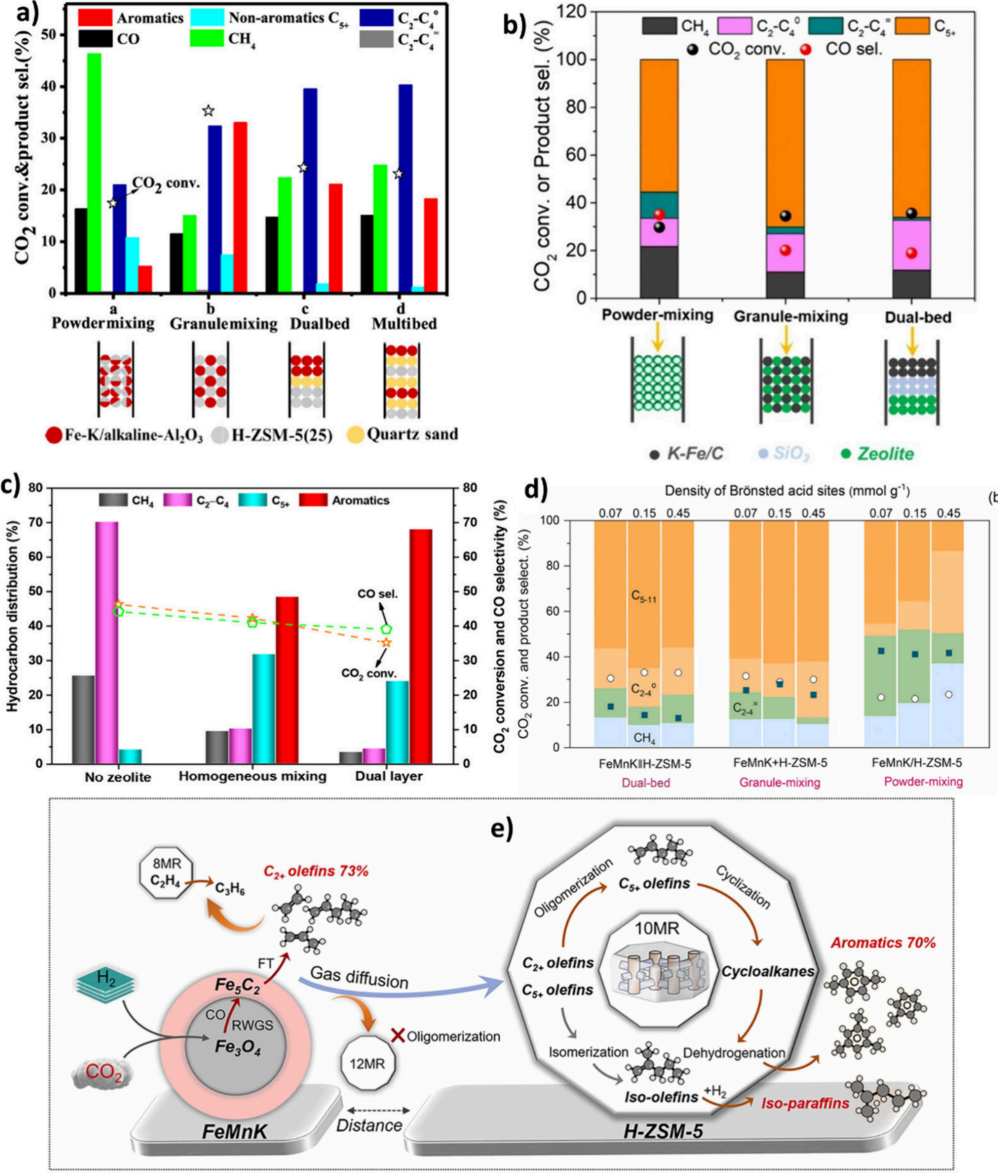
**a)** Effect of integration manner of Fe-K/a-Al_2_O_3_ with HZSM-5(25) on the CO_2_ hydrogenation performance. Reaction conditions: H_2_/CO_2_ = 1, 3 MPa, 400 °C, and 3000 mL g_cat_^–1^ h^–1^. Reproduced with permission from Dai et al.^212^ Copyright permission obtained from ACS, 2020. **b)** Influence of different integration manner of K­(6 wt %)-Fe/C and K-ZSM-5 tandem catalysts on CO_2_ hydrogenation performance. Reaction operating conditions: H_2_/CO_2_ = 2.5, mass ratio of K-Fe/C to zeolite = 1/3, 320 °C, 2.0 MPa, and 1200 mL g^–1^ h^–1^ (4800 mL g^–1^ h^–1^ for K-Fe/C). Reproduced with permission from Guo et al.^220^ Copyright permission obtained from Elsevier, 2021. **c)** CO_2_ hydrogenation performance of FeK1.5/HSG assembled in different manners with HZSM-5(50) (Reaction conditions: 339.85 °C, 2.0 MPa, and 26000 mL g^–1^ h^–1^). Reproduced with permission from Wang et al.^203^ Copyright permission obtained from ACS, 2019. **d)** The effect of proximity of K-Mn-Fe and HZSM-5(27) and BAS density on product distribution, Reaction conditions: 3 MPa, 320 °C, and 6000 mL g^–1^ h^–1^. **e)** Reaction pathway for CO_2_ hydrogenation using FeMnK+H-ZSM-5 tandem catalyst, emphasizing the effects of distance and zeolite pore dimension on the product distribution. Reproduced with permission from Li et al.^86^ Copyright permission obtained from Elsevier, 2023.

Moreover, the method of K incorporation to the Fe-oxide has been found to exert a significant effect on product distribution. In this context, Han et al.^323^ also showed that the physical mixing of Fe/C and K_2_CO_3_ and then making pellets provided the best performance in CO_2_ hydrogenation to olefins. Using HR-TEM and FFT, it was observed that the proximity of K_2_CO_3_ and Fe/C affected the morphology of the catalyst, and the physical mixing case resulted in the faster carburization of metallic iron to Fe_5_C_2_. Ramirez et al.^324^ also reported the formation of more aromatics using physically mixed Fe_2_O_3_ and KO_2_ (Fe_2_O_3_@KO_2_) when integrated via the D.B. mode with HZSM-5(600). Therefore, a physical mixture of K_2_CO_3_ and Fe/C was prepared and mixed with K-promoted HZSM-5(24) at different proximities for the production of heavy liquid hydrocarbons (Figure 35 (b)). It is noteworthy that exploiting alkali ions like K^+^ to treat zeolite could reduce the amount of strong BAS on the H-ZSM-5 surface and modulate the acid density for the production of heavier hydrocarbons in the gasoline range.^220^ The results revealed that in a composite catalyst integrated by the G.S. mode, the distance between active sites was increased, leading to isomerization, aromatization, and oligomerization of alkene intermediates. This resulted in a notable selectivity for liquid hydrocarbons (approximately 70.1%). On the other hand, the D.B. configuration led to a further increase in distance between active sites, yielding a catalytic performance similar to the composite catalyst integrated by G.S. However, due to a greater extent of hydrogenation, more C_2_–C_4_ paraffin formed in the D.B. mode.^220^

Wu et al.^325^ utilized honeycomb-structured graphene (HSG) with K as the promoter to create Fe-K/HSG catalysts, which demonstrated high efficiency in FT. The distinctive three-dimensional structure of HSG effectively prevents the agglomeration of iron carbide nanoparticles, while its large pores enable unrestricted diffusion of reactants and products, facilitating the catalytic process. In addition, Wang et al.^203^ showed that the highest aromatic selectivity could be obtained when Fe-K/HSG integrated with HZSM-5(50) in a D.B. manner. It is suggested that not only the ZSM-5 acidity, metal oxide basicity, and their synergy at the metal oxide/zeolite interface but also the nature of the second phase (HSG in this catalyst) has a vital role in the proximity of active sites. It was revealed that Fe-K/HSG was active in light olefins formation.^236^ However, in the G.S. mode of Fe-K/HSG and HZSM-5, the close proximity of the active sites resulted in further hydrogenation and hydrogenolysis of the produced heavy aromatics in the H-ZSM-5 pores, which transferred back to the hydrogenation sites of Fe-K^203^ through HSG. This might be attributed to the large pores of HSG that facilitate the diffusion of reactants and products.^325^ Therefore, by increasing the distance via the D.B. configuration, the diffusion of products through HSG and their further hydrogenation were inhibited, and more aromatics were produced, as can be observed in Figure 35 (c).

Moreover, it was confirmed that the copromotion of K and other elements can alter the CO_2_ hydrogenation performance in terms of product distribution.^151,326^ Recently, Li et al.^86^ observed that coupling FeMnK and HZSM-5(47) and increasing the distance between active sites improved the catalytic performance of CO_2_ hydrogenation toward C_5+_ considerably. For instance, the CO and CH_4_ selectivity decreased from 41 to 14% and from 19 to 9.5%, respectively, while the CO_2_ conversion and selectivity to C_5+_ hydrocarbons rose from 22 to 33% and from 37 to 65%, respectively, when increasing the distance from powder mixing granules to the D.B. mode (Figure 35 (d)).^86^ It was revealed that the olefins formed on the active Fe_5_C_2_ could diffuse into 10MR zeolite micropores if assembled at the optimized distance and oligomerized into C_5+_ hydrocarbons on BAS, which further can be transformed into aromatics or iso-paraffins as shown in Figure 35 (e).

As a whole, in the most of the above-mentioned cases of K-promoted Fe-based catalysts, the D.B. integration manner with ZSM-5 provided the highest selectivity toward aromatics, and the D.B. mode provided a larger Aro/non-Aro ratio. Moreover, it can be inferred that the physical mixing of potassium salts such as KO_2_ ^321^ and K_2_CO_3_ ^323^ with iron oxide enhances the formation of more light olefins, which are the intermediates for aromatic production in the presence of ZSM-5. In addition, promoting the support instead of metal oxide or dispersing the K-promoted iron oxide over support like Al_2_O_3_ may increase the distance between basic active sites of metal oxide and BAS, compared to unsupported catalysts, which necessitates closer proximities between oxide and zeolite.^212,287^ However, mesoporous supports such as HSG, which facilitates the diffusion of both reactants and products, necessitate distant proximity to avoid hydrogenation of the intermediates before entering the zeolite pores.^203^

## 7 Discussion and Perspectives

### 7.1 Analysis and Trends

CO_2_ hydrogenation via tandem catalysis has attracted considerable attention as a potent approach for suppressing CO_2_ emissions while producing valuable chemicals and fuels.^327,328^ According to the discussion in section 6, it has been confirmed that simply mixing catalysts together in a random manner fails to adequately manage the progression of desired reactions. Consequently, meticulous attention must be given to establishing the precise arrangement and structure of catalytic sites within the tandem catalyst to effectively govern the conveyance of essential intermediates.

Based on the above knowledge and the investigation of literature data, it can be speculated that the interactions between Fe-based oxide (basic sites) and zeolite (acidic sites), which can be altered at different proximities, play a critical role in determining the hydrocarbon distribution. Notably, at very close proximity, strong interactions can result in detrimental phenomena such as elemental migration and mutual poisoning of the active sites. Poisoning the metallic basic sites can limit carburization and carbide formation, thereby reducing the formation of intermediate hydrocarbons. In addition, poisoning the acid sites of zeolites can neutralize them and hinder further oligomerization and aromatization. Moreover, if the active sites are located far apart, the intermediates cannot transfer appropriately from the Fe-based oxide to the zeolite active sites and may undergo unwanted hydrogenation. Additionally, modifying Fe-based catalysts with different promoters and/or incorporating other metals can impact CO_2_ conversion and product distribution. However, to tune product distribution, an appropriate zeolite topology with the optimized BAS should be selected. Notably, the modifications of both basic and acidic sites, as well as their density, can affect the proximity of the active sites.

According to the above outcomes, the appropriate selection of active sites, promoters, zeolite topology, and BAS strength, when combined at an optimized distance and configuration, can result in the formation of the desired hydrocarbons. Drawing connections and insights from the existing literature in this field is beneficial for establishing a cohesive comprehension of how the mentioned parameters contribute to the overall understanding of CO_2_ activation and selective conversion. In this context, analyzing the performance of K- and Na-promoted Fe-based catalysts in different integration modes revealed that, although they both belong to the alkali elements, each might induce a different influence at a definite proximity. To further investigate the interaction of alkali promoters and proximity, the performance of alkali-promoted iron oxide in the presence of ZSM-5 is summarized in Table 1.

**Table 1:** Performance of Alkali-Promoted Fe-Based Catalysts with Different Catalyst Configurations

Catalysts										HC distribution								
		I.M^a^	T^b^	P^c^	GHSV^d^	F.R^e^	Ox/Z^f^	X_CO2_	S_CO_	C_1_	C_2_–C_4_^0^ ^i^	C_2_–C_4_^=^ ^j^	N-Aro^l^	Aro^k^	Aro/N-Aro	Y_C5+_ ^g^	STY_C5+_ ^h^	Ref.
1	Na-Fe_3_O_4_ & HZSM-5(160)	G.S.	320	3	4000	3	1/1	33.60	14.20	7.90	18.40		17.39	56.31	3.24	21.25	9.11	^201^
2		D.B.	320	3	4000	3	1/1	33.60	14.42	9.10	23.60		26.02	41.28	1.59	19.35	8.29	
3	Na-Fe_3_O_4_ & HZSM-5(12.5)	G.S.	340	1	4800	3.1	1/2	15.1	>98	-	-	-	-	-	-	-	-	^209^
4		D.B.	340	1	4800	3.1	1/2	33.9	24.8	20.1	53.5	0.3	0.4	25.7	64.25	6.65	3.48	
5	Na-Fe_3_O_4_ & HZSM-5(30)	G.S.	320	3	4000	2	1/1	20.90	23.30	9.10	11.70	23.20	5.50	50.50	9.18	8.98	5.13	^309^
6		D.B.	320	3	4000	2	1/1	26.50	16.10	6.60	9.40	17.20	10.80	56.00	5.19	14.85	8.49	
7	ZnFeOx-4.25Na & HZSM-5(25)	G.S.	320	3	4000	3	1/2	36.00	11.00	8.22	13.33	3.17	15.40	60.06	3.90	24.18	10.36	^210^
8		D.B.	320	3	4000	3	1/2	34.06	12.94	8.35	16.89	3.17	22.46	49.26	2.19	21.27	9.11	
9	NaZnFe13 & HZSM-5-0.5	G.S.	320	3	1000	3	1/1	42.5	6.15	7.4	15.22	0	13.45	63.92	4.75	30.92	3.31	^313^
10		D.B.	320	3	1000	3	1/1	38.13	10.34	11.36	22.37	0	15.02	50.33	3.35	22.34	2.39	
11	Na-Fe@C & HZSM-5-0.2 M	G.S.	320	3	9000	2.95	1/3	33.30	13.30	4.80	9.60	0.80	34.60	50.20	1.45	24.63	24.05	^45^
12		D.R.	320	3	9000	2.95	1/3	29.50	15.00	7.30	5.50	2.10	39.00	46.10	1.18	21.34	20.83	
13	Na-FeAlOx/Zn-ZSM-5(12.5)@SiO_2_	M.G.	370	3.5	4000	3	1/1	45.20	15.30	13.8	26.26		21.14	38.7	1.83	22.91	10.23	^216^
14		D.B.	370	3.5	4000	3	1/1	31.38	28.82	11.36	28.29		54.33	6.02	0.11	13.48	6.02	
15	2.3Na-CuFe_2_O_3_ and HZSM-5(25)	G.S.	320	3	1000	3	1/1	33.3	16	11.91	15.63	-	3.81	69.05	18.13	20.38	2.18	^142^
16		D.B.	320	3	1000	3	1/1	28.12	19.37	14.34	22.87	3.88	12.02	47.55	3.96	13.51	1.45	
17	2.83Na-FeMn(90/10) & HZSM-5@S1-S	G.S.	320	3	4000	2.73	1/1	21.7	32	9.6	13.3	26.7	33.4	17	0.51	7.44	3.41	^156^
18		D.B.	320	3	4000	2.73	1/1	22.7	23.4	12.2	16.1	37.8	28.5	5.4	0.19	5.89	2.71	
19	15Fe-10K/a-Al_2_O_3_ and P/HZSM-5(25)	M.G.	400	3	3000	1	1/1	17.50	16.30	55.32	25.09	0.36	12.90	6.33	0.49	2.82	1.89	^212^
20		G.S.	400	3	3000	1	1/1	35.30	11.50	16.95	36.61	0.68	8.47	37.29	4.40	14.30	9.57	
21	FeK1.5/HSG|HZSM-5(50)	G.S.	340	2	26000	3	1/1	42.34	41.14	9.50	10.18		31.80	48.40	1.52	19.99	55.68	^203^
22		D.B.	340	2	26000	3	1/1	35.00	39.00	3.50	4.40		24.00	68.00	2.83	19.64	54.72	
23	Fe_5_C_2_ and 10K/a-Al_2_O_3_	M.G.	400	3	3600	3	0.133/0.867	44.84	23.24	30.09	6.25	29.83	33.87		NA	11.66	4.68	^287^
24		G.S.	400	3	3600	3	0.133/0.867	43.87	21.40	37.40	17.68	21.88	23.03		NA	7.94	3.19	
25	Fe_2_O_3_@KO_2_/ZSM-5(600)	M.G.	375	3	5000	3	1/1	NA	31.38	31.40	8.86	41.90	16.85	0.98	0.06	NA	NA	^324^
26		D.B.	375	3	5000	3	1/1	48.90	12.61	15.29	32.65	11.25	13.83	26.98	1.95	17.44	9.73	
27	K-FeC_2_O_4_2H_2_O and K-ZSM-5(29)	M.G.	320	2	1200	2.5	1/3	29.75	35.07	21.61	11.99	10.86	55.54		NA	10.73	1.56	^220^
28		G.S.	320	2	1200	2.5	1/3	34.5	20	10.86	16.18	2.83	70.1		NA	19.35	2.81	
29	FeMnK/HZSM-5(47)	M.G.	320	3	6000	3	1/1	22	41	19	13	32	37		NA	4.8	3.09	^86^
30		D.B.	320	3	6000	3	1/1	33	14	9.5	17	8.10	65		NA	18.45	11.86	

a Integration manner of active sites.

b (°C).

c (MPa).

d (mL g^–1^ h^–1^).

e (Oxide/ZSM-5 ratio).

f Feed ratio (H_2_/CO_2_).

g C_2_–C_4_^0^: paraffins.

h C_2_–C_4_^=^: olefins.

i N-Aro: Non-Aromatics.

j Aro: Aromatics.

k Yield of C_5+_ hydrocarbons.

l (mmol g_cat_^–1^ h^–1^).

The aromatic to non-aromatic hydrocarbons (Aro/N-Aro) ratio is observed to be higher than one in most Na-promoted catalysts, contrary to many of K-promoted samples. The data provided in Table 1 shows that by increasing the distance between active sites (mainly from G.S to D.B), the Aro/N-Aro ratio in Na-promoted catalysts decreases, which is in contrast to K-promoted catalysts where the ratio often increases, as illustrated in Figure 36 (a) and (b). Moreover, Figures 36 (c) and (d) show that selectivity toward CO and CH_4_ in K-promoted catalysts is higher than the corresponding values in Na-promoted catalysts. This can be attributed to the more basic nature of K that facilitates carburization and thus the formation of CH_*x*_, as discussed in section 4.1.

**Figure 36:**
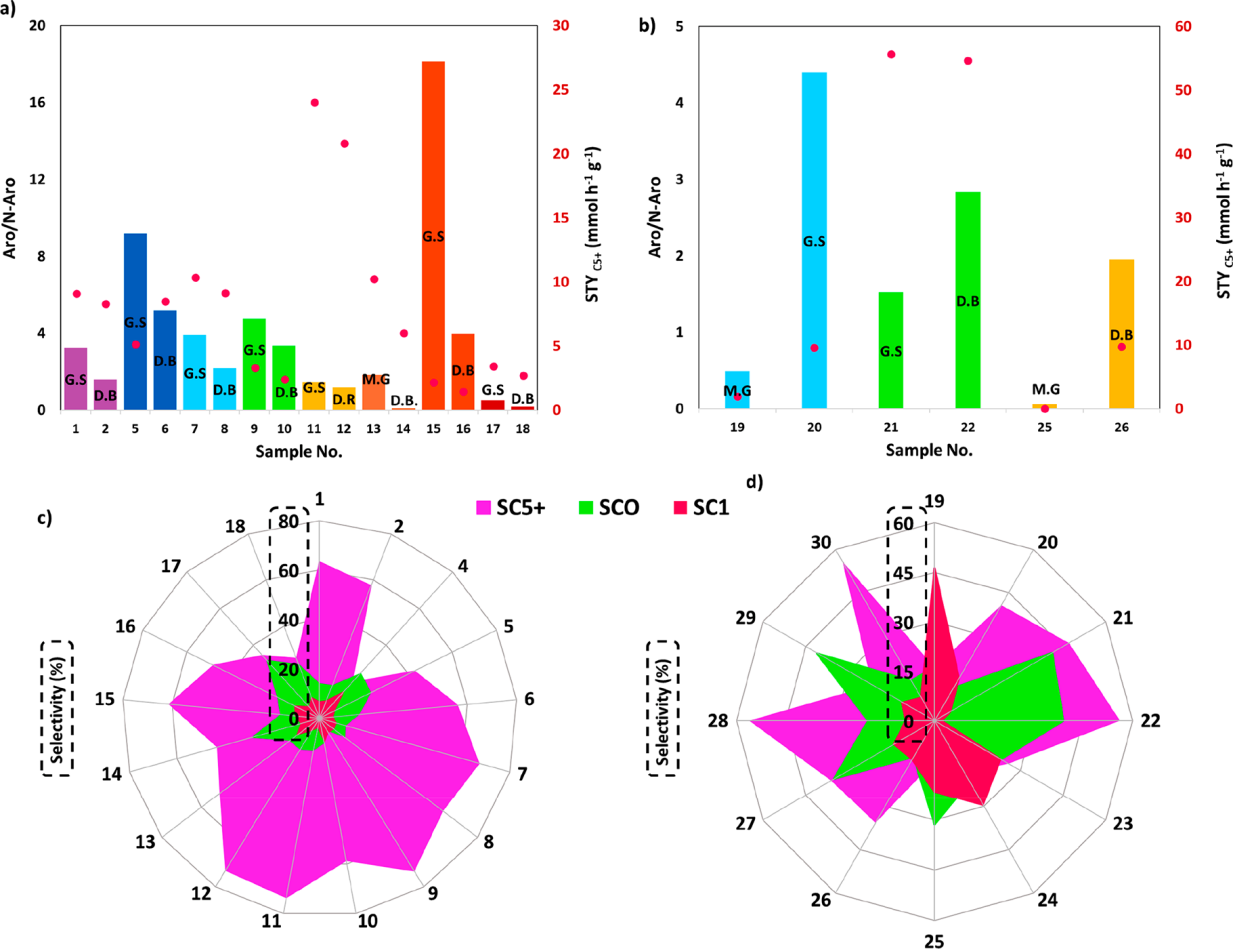
Aro/N-Aro (columns) and STY_C5+_ (circles) for **a)** Na-promoted catalysts, **b)** K-promoted catalysts (Note: Only the data for which the proportions of aromatic and nonaromatic C_5+_ were available were used in these Figures). Selectivity of CO, CH_4_, and C_5+_ (including CO) for **c)** Na-promoted catalysts and **d)** K-promoted catalysts (Sample numbers refer to entries from Table 1) (Created by authors).

It is evident that by increasing the distance between alkali-promoted iron oxide and zeolite, the alkali migration from oxide to zeolite is suppressed. Accordingly, the influence of Na and K on hydrogenation of the hydrocarbons formed mainly on the surface of the zeolite is decreased. However, the extent of this decrease is different according to the nature and loading of the promoter, and K likely has a higher capacity for reducing the BAS sites compared to Na. Therefore, heavy hydrocarbons over Na-promoted Fe-oxide undergo more hydrogenation reactions than aromatization when the distance between Fe-oxide and ZSM-5 increases. Such a configuration leads to a decreased Aro/N-Aro ratio. On the other hand, for a K-promoted catalyst, with increasing distance, hydrogenation becomes more difficult; in turn more aromatization reactions proceed, which increases the Aro/N-Aro ratio. Accordingly, the distance between Na-promoted Fe-oxide and zeolite should be increased if the desired product is non-aromatic hydrocarbons. In contrast, for K-promoted ones, a closer proximity is required for non-aromatic formation. The influence of Na and K on the Aro/N-Aro ratio with increasing distance between iron oxide and zeolite is illustrated in Figure 37.

**Figure 37:**
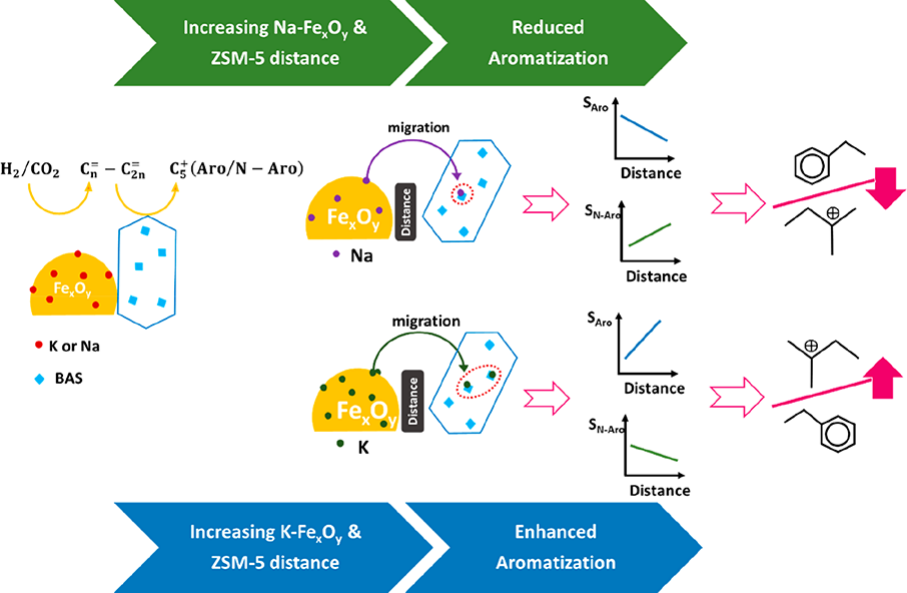
Effect of Na and K on Aro/Iso ratio with increasing the Fe-oxide and zeolite distance (Designed by the authors).

However, the role of operating conditions, particularly GHSV, cannot be ignored when evaluating CO_2_ hydrogenation performance to produce liquid fuels. In other words, if the performance (yield) of different catalysts at different operating conditions is compared, disregarding the aforementioned parameters, the comparison may result in erroneous results. Accordingly, the performance data of various catalysts containing different promoters with different zeolites and BAS treatments at different GHSVs are collected (Table 2) and plotted in terms of C_5+_ selectivity vs CO_2_ conversion (Figure 38 (a)), as well as in terms of C_5+_ yield vs C_5+_ STY (Figure 38 (b)), where the dotted line shows the C_5+_ yield in Figure 38 (a). One of the major parameters commonly used in measuring the efficiency of the catalyst in converting reactants to products, taking into account the amount of product formed per weight of the catalyst at a definite time, is the space–time yield (STY).^329^ STY provides the comparison of different catalysts, considering inlet flow rate and catalyst weight in one parameter known as gas hourly space velocity (GHSV). In fact, STY is directly proportional to (Conversion × Selectivity × GHSV). By measuring and comparing the STY of different catalysts or reaction conditions, industrial researchers can identify the most efficient and productive systems.^261,268,330^ Additionally, STY offers valuable insights into the feasibility of scaling up a reaction from a laboratory to an industrial scale. By assessing STY at different scales, scientists and engineers can estimate process productivity and efficiency in large-scale operations. This knowledge is instrumental in designing and planning industrial reactors and determining their production capacities.^331^ Furthermore, STY has a direct correlation with the economics of a process. Higher STY values typically imply greater productivity, resulting in cost savings and increased profitability. Maximizing the STY allows industrial processes to achieve higher yields of desired products using less catalyst and shorter reaction times, thereby reducing operating costs and improving overall process efficiency.^332–334^

**Table 2:** CO_2_ Hydrogenation Performance of Different Catalysts in Terms of Y and STY of C_5+_ Hydrocarbons

No.	Catalysts	XCO_2_	T^a^	P^b^	GHSV^c^	SC_5+_ ^d^	YC_5+_ ^e^	STYC_5+_ ^f^	Ref.
1	FeMnK/H-SSZ-13(7)	30	320	3	6000	31.36	9.41	6.05	^86^
2	FeMnK/H-ZSM-35(10)	31				32.55	10.1	6.49	
3	FeMnK/H-MCM-22(17)	33				42.50	14	9.02	
4	FeMnK/H-ZSM-22(160)	32				44.14	14.1	9.08	
5	FeMnK/H-ZSM-5(153)	32				49.20	15.7	10.12	
6	FeMnK/H-ZSM-5(93)	32				46.13	14.8	9.49	
7	FeMnK/H-ZSM-5(47)	27				39.88	10.8	6.92	
8	FeMnK/H-ZSM-5(14)	30				47.74	14.3	9.21	
9	FeMnK/H-ZSM-48(85)	31				37.60	11.7	7.49	
10	FeMnK/H-ZSM-48(45)	31				38.38	11.9	7.65	
11	FeMnK/H-MOR(13)	32				32.32	10.3	6.65	
12	FeMnK/H-Beta(27)	32				29.80	9.53	6.13	
13	FeMnK/H-Y(3)	37				33	12.2	7.85	
14	Na-Fe@C/H-ZSM-5(40.5)	32.10	320	3	9000	68.61	22	21.50	^45^
15	Na-Fe@C/H-ZSM-5-0.1M(35.2)				30.20	69.69	21	20.55	
16	Na-Fe@C/H-ZSM-5-0.2M(32.8)				33.30	73.52	24.5	23.90	
17	Na-Fe@C/H-ZSM-5-0.4M(25.7)				30.40	54.29	16.5	16.11	
18	K-Fe-Cu-Al/HZSM-5(400)	45.39	320	3	4000	67.41	30.6	13.14	^211^
19	K-Fe-Cu-Al/HZSM-5(200)	45.91				68.73	31.5	13.55	
20	K-Fe-Cu-Al/HZSM-5(85)	44.48				56.02	24.9	10.70	
21	K-Fe-Cu-Al/HZSM-5(50)	43.19				52.43	22.6	9.72	
22	K-Fe-Cu-Al/HZSM-5(25)	44.35				53.17	23.6	10.13	
23	Na-FeMnOx/HZSM-5	44.63	320	3	1000	71.98	32.1	3.44	^157^
24	Na-FeMnOx/0.1P-HZSM-5	43.11				75.16	32.4	3.47	
25	Na-FeMnOx/0.5P-HZSM-5	42.88				74.09	31.8	3.40	
26	Na-FeMnOx/1P-HZSM-5	41.76				65.42	27.3	2.93	
27	Na-FeMnOx/5P-HZSM-5	38.02				44.01	16.7	1.79	
28	Na-FeMnOx/HZSM-5(25)	44.50				74.20	33	3.54	
29	Na-FeMnOx/HZSM-5(40)	43.17				70.42	30.4	3.26	
30	Na-FeMnOx/HZSM-5(60)	41.57				71.27	29.6	3.17	
31	Na-FeMnOx/HZSM-5(100)	39.81				64.89	25.8	2.77	
32	Na-FeMnOx/HZSM-5(200)	34.47				52.96	18.3	1.96	
33	F1MZnZS(12.5)	45.30	370	4	4000	50.31	22.8	10.17	^216^
34	F1MZnZS(100)	41.10				45.66	18.8	8.38	
35	FZS(12.5)	44.12				39.43	17.4	7.77	
36	F1.5MZnZS(12.5)	48.56				33.83	16.4	7.33	
37	F2MZnZS(12.5)	52.33				37.61	19.7	8.79	
38	F1MZnZ(12.5)	39.71				37.17	14.8	6.59	
39	F1MZnZS-2(12.5)	44.16				40.17	17.7	7.92	
40	2.83Na-FeMn(10/90)/HZSM5(24)	28.10	320	3	4000	43.15	12.1	5.96	^156^
41	2.83Na-FeMn/HZSM5(40)	27.60				42.05	11.6	5.70	
42	2.83Na-FeMn/HZSM5(105)	27				48.89	13.2	6.49	
43	2.83Na-FeMn/HZSM5(300)	25.20				43.26	10.9	5.36	
44	2.83Na-FeMn/HZSM5(1500)	22.70				25.42	5.77	2.84	
45	2.83NaFeMn(10/90)/HZSM5@S1-S	20.70				33.78	6.99	3.44	
46	K-Fe-C/H-ZSM-5(24)	37.20	320	2	1200	32.68	12.2	2.02	^220^
47	K-Fe-C/K-ZSM-5(29)	34.50				56.92	19.6	3.26	
48	K-Fe-C/H-ZSM-5*(28)	33.70				40.77	13.7	2.28	
49	Fe-C/HZSM5(50)	31.66	320	2	4000	25.28	8.01	3.43	^208^
50	Fe-C/HZSM5(160)	33.19				32.79	10.9	4.66	
51	Fe-C/HZSM5(300)	32.10				18.77	6.02	2.58	
52	FeK1.5-HSG/HY	35.99	340	2	26000	26.58	9.56	26.64	^203^
53	FeK1.5-HSG/HB	36.86				34.40	12.7	35.32	
54	FeK1.5-HSG/HMCM22	38.06				41.82	15.9	44.34	
55	FeK1.5-HSG/NaZSM5(50)	37.95				43.64	16.6	46.13	
56	FeK1.5-HSG/HZSM5(27)	37.19				55.48	20.6	57.48	
57	FeK1.5-HSG/HZSM5(50)	35.22				56.11	19.8	55.05	
58	FeK1.5-HSG/HZSM5(160)	36.97				55.24	20.4	56.89	
59	FeZn-Zr@HZSM5(38)	23	340	5	3000	24.30	5.59	1.72	^300^
60	FeZn-Zr@HZSM5(50)	21.50				32.14	6.91	2.13	
61	FeZn-Zr@HZSM5(100)	23.50				9.28	2.18	0.67	
62	FeZn-Zr/HZSM5(50)	20.10				23.06	4.63	1.43	
63	NaFe/HZSM23	34.36	320	3	4000	44.39	15.3	6.54	^201^
64	NaFe/HMCM22	34.84				50.11	17.5	7.48	
65	NaFe/HZSM5(27)	33.75				58.82	19.9	8.51	
66	NaFe/HZSM5(160)	33.75				63.12	21.3	9.13	
67	NaFe/HZSM5(300)	32.85				58.24	19.1	8.20	
68	CuFeO_2_/HZSM5(150)	45.40	320	3	8100	66.81	30.3	24.68	^144^
69	CuFeO_2_/HZSM5(65)	47.90				71.53	34.3	27.88	
70	CuFeO_2_/HZSM5(25)	46.10				69.74	32.1	26.16	
71	CuFeO_2_/HZSM5(12.5)	45				64.28	28.9	23.53	
72	5K-CoFeOx(1:5)/HZSM5(27)-Si	51.26	320	3	4000	55.74	28.6	12.25	^119^
73	5K-CoFeOx(1:5)/HZSM5(27)	42.27				39.86	16.8	7.22	
74	5K-CoFeOx(1:5)/HZSM5(60)	42.36				34.43	14.6	6.25	
75	5K-CoFeOx(1:5)/HZSM5(130)	43.96				37.17	16.3	7.00	
76	Fe@K/m-Z5	34.73	325	3	10000	32.28	11.2	11.51	^199^
77	Fe@K/h-Z5(bar,cellu)	35.99				36.57	13.2	13.51	
78	Fe@K/h-Z5(bar,glu)	35.07				38.88	13.6	14.00	
79	Fe@K/h-Z5(coffin,cellu)	35.99				39.14	14.1	14.47	
80	Fe@K/h-Z5(coffin,glu)	34.27				36.37	12.5	12.80	

a (°C).

b (MPa).

c (mL g^–1^ h^–1^).

d (Selectivity of C_5+_ hydrocarbons including CO).

e (Yield of C_5+_).

f (mmol g_cat_^–1^ h^–1^).

**Figure 38:**
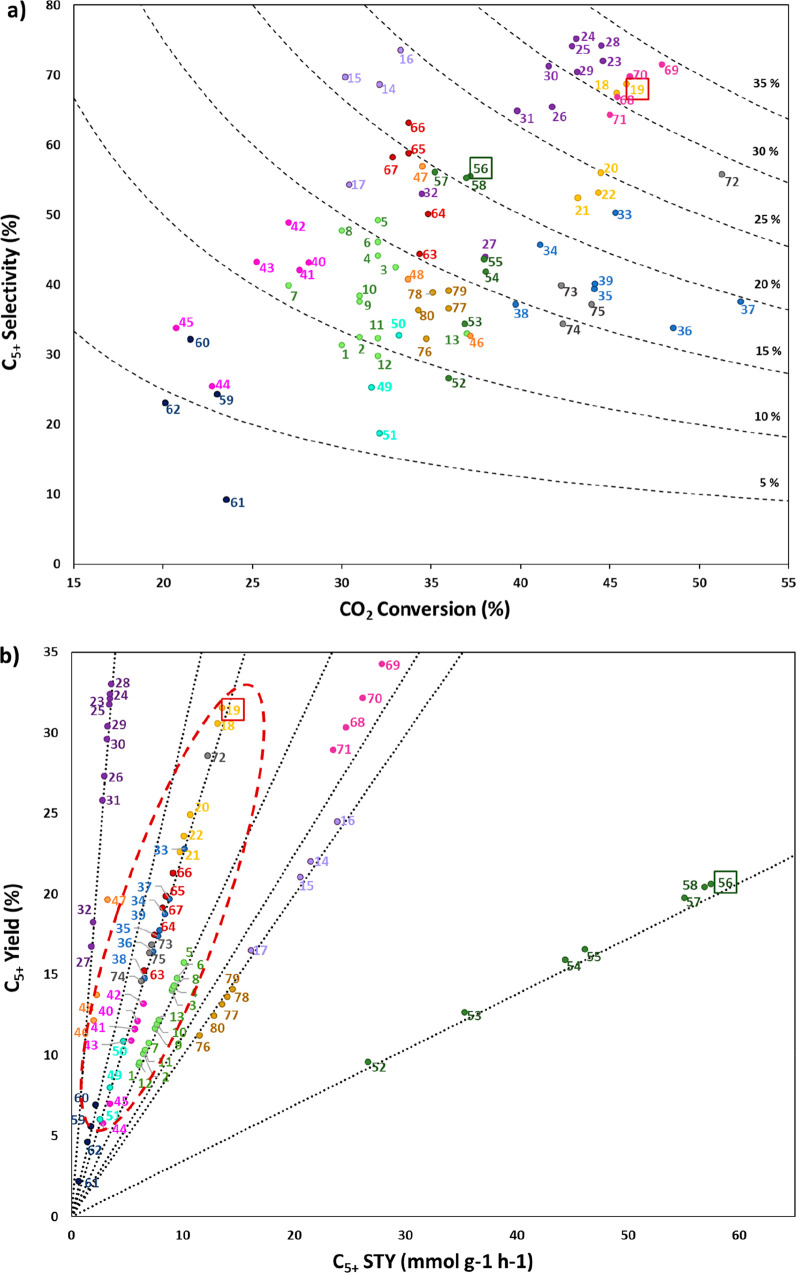
CO_2_ hydrogenation performance of the recently used catalysts: **a)** C_5+_ selectivity vs CO_2_ conversion and **b)** C_5+_ yield vs C_5+_ STY (Labels refer to entries from Table 2) (Created by authors).

For instance, it can be observed in Figure 38 (a) that samples 15 (NaFe@C/ZSM5–0.1M(35)),^45^ 66 (NaFe/ZSM5(160)),^201^ 56 (FeK1.5@HSG/ZSM5(27)),^203^ 21 (KFeCuAl/ZSM5(50)),^211^ and 33 (F1MnZnZSM5-S(12.5))^216^ have almost the same performance and C_5+_ yield (around 21–22%), while calculating the C_5+_ STY (around 57.5 mmol_C5+_ gr_cat_^–1^ h^–1^ in Figure 38 (b)) shows that sample 56 exhibits considerably higher performance among others due to the high GHSV (26,000 mL g^–1^ h^–1^).

Moreover, based on the available literature, it is revealed that the majority of research studies investigated catalyst performance at GHSV = 4000 – 6000 mL g^–1^ h^–1^ (the region shown by dotted oval in Figure 38 (b)), and therefore, the C_5+_ STY cannot go higher than a certain amount, which is around 13.55 mmol/g^–1^ h^–1^ for sample 19 (KFeCuAl/ZSM5(200)),^211^ as displayed in Figure 38 (b). In addition, it is obvious that modifying BAS via various zeolite treatments and using different zeolite topologies mainly alter the C_5+_ selectivity (for example, samples 1–13 (FeMnK/zeolite)^86^ and 18–21 (KFeCuAl/zeolite)),^211^ as illustrated in Figure 38 (a). In contrast, catalyst modification via changing the promoter loading commonly changes CO_2_ conversion (for example, samples 34–39 (xZn-yMn-Fe/ZSM5)).^216^

Therefore, given that this field of study is still in its early stages, further fundamental understanding is necessary to enhance the efficiency of these catalysts in terms of both CO_2_ conversion and hydrocarbon selectivity.

### 7.2 Contributions of Nanoscale Proximity to Reactor Configuration

Preparing and synthesizing effective catalyst materials are pivotal in achieving high-performance processes. Importantly, the key catalytic material/component does not necessarily have to be entirely new; instead, it often involves improving the original and well-studied catalytic material. Developing novel materials can begin with exploring structural composition and spatial arrangement.^335^ Based on the obtained knowledge, it can be speculated that appropriate proximity between the active site of basic Fe-oxide and acidic zeolite significantly affects the catalyst performance. Moreover, by adjusting the proximity and tailoring the optimal combination of basic and acidic sites, the distribution of hydrocarbon products can be tuned toward either aromatics or iso-paraffins. Therefore, the effective diffusion of intermediate molecules is crucial to obtain the desired product distribution. This necessitates precise regulation of the spatial distribution and connectivity of catalytic species to enhance diffusion paths. Therefore, the methodologies by which acid–base active sites can be segregated or compartmentalized are of paramount importance in designing high-performing CO_2_ hydrogenation catalysts.^336^

#### 7.2.1 At the Nanoscale

The proximity and arrangement of active sites can be finely controlled and tuned through various synthesis techniques, as described in section 6.2, which enable the manipulation of the active site arrangement and the overall structure of nanomaterials. For instance, in core–shell nanomaterials, also known as nanoreactors, the core size and the shell thickness can be tailored to optimize the exposure of active sites on the surface. This adjusted proximity of active sites can significantly enhance the catalytic efficiency and reactivity of the nanomaterial.^250,337^ From an engineering perspective, the central goal is always to design and enhance specific properties or functions of a material. In this context, various forms of hierarchy are incorporated into the multiple-scale structures, which can be utilized in industrial catalytic processes. Therefore, such entities exhibiting different chemical properties and functions can be coupled over a length scale of more than 10 orders of magnitude (Figure 39).^233^

**Figure 39:**
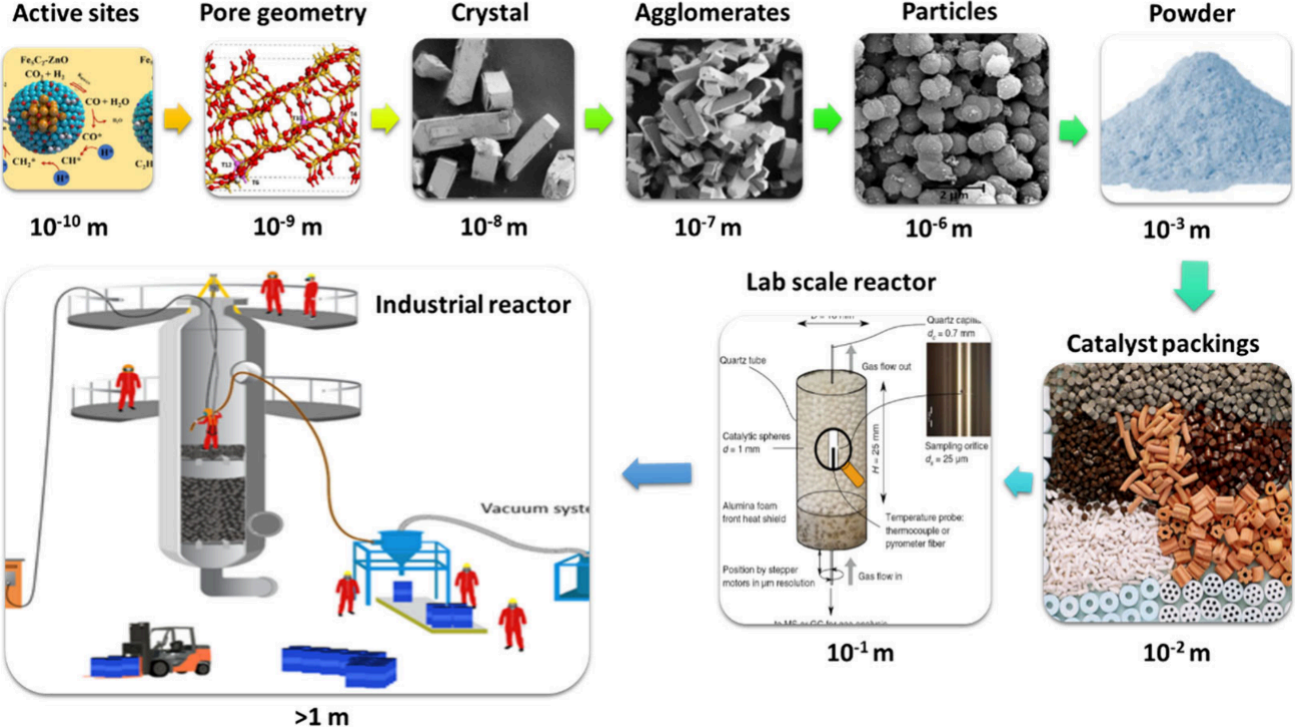
Hierarchy of length scales from active sites to reactor; active sites,^338^ pore geometry,^339^ crystal,^340^ agglomerates,^340^ particles,^341^ powder,^342^ catalyst packings,^343^ lab-scale reactor,^344^ industrial reactor.^345^ Reconceptualized and redrawn with permission from Mitchell et al.^342^ Copyright 2013, RSC. Reproduced with permission from the above-mentioned refs Copyright permission obtained from Elsevier, 2021; John Wiley & Sons, 2017; Elsevier, 2023; John Wiley & Sons, 2016; Catalysts Europe Weblog.

Considering the analysis presented in section 7.1 and the concept of different scales (from active sites to catalyst pellets) presented in Figure 39, one of the emerging solutions to take advantage of the excellent dispersion capability of mesoporous supports while tuning the sequence of reaction (first RWGS and then FT) can be a core–shell structure. Accordingly, the core comprises a mesoporous-supported Fe-based catalyst, while the appropriate zeolite, which can be selected based on the desired fuel distribution, would form the shell. Moreover, more effort can be devoted to incorporating the functional components of active CO_2_ hydrogenation catalysts into a fully integrated platform with diverse structures by 3D printing.^346^ In fact, 3D printing can assemble the components with multiple structures in a preferred proximity to tune the product distribution.^268^ Mechanical strength, intensified heat transfer, reduced pressure drop, and precise adjustment of size, shape, and porosity are other advantages of 3D-printed catalysts.^267^ These unique properties make 3D printing a promising alternative approach for the large-scale production of tandem catalysts for CO_2_ hydrogenation to a variety of chemicals and fuels.^273,347,348^

#### 7.2.2 In the Context of the Macroscale

A limited number of studies have investigated the influence of the reactor type and process intensification in CO_2_ hydrogenation.^349–351^ The chosen reactor configuration should match the reaction steps over tandem catalysts to achieve a high yield. The organization of rectors and selective transportation of reactive chemical components are crucial elements in chemical transformations within the cells. Radial-flow packed beds (RPBs) represent a new and innovative form of structured packed beds that combine the benefits of both traditional packed beds (PBs) and structured packed beds. An RPB typically has a cylindrical, fluid-permeable separating wall that divides the bed into inner and outer regions. This unique design feature sets RPBs apart and offers distinct advantages compared to conventional and structured packed beds. The modeling study of this reactor configuration confirmed improved efficiency in mass and heat transfer, as well as reduced pressure drop, during the dry reforming of methane. The presence of the separating wall ensures a uniform distribution of catalyst pellets, resulting in less convoluted flow channels. As a result, flow resistances are reduced, and there is improved axial convective heat transfer within system^352^ (Figure 40 (a)). However, applying the proposed reactor configuration to segregate reactions has not been thoroughly investigated and understood. The thickness of its wall can be modulated to tune the proximity of active sites, which are separated via the tube wall. Moreover, suppose another tube, which is permeable to water, can be inserted into the inner or outer tube.^353,354^ In that case, one can take advantage of both RPB and membrane reactor (MR) technologies to remove water in situ, as demonstrated in Figures 40 (b) and (c). This configuration can direct the flow toward the wall, hinder the backflow of intermediates to the basic metallic sites, convey the intermediates toward acid sites of zeolite, and avoid mutual poisoning of active sites. Therefore, it can be employed in CO_2_ hydrogenation reactions by segregating or compartmentalizing the reactor through a radially configured reactor design, ensuring that RWGS and FTS occur successively.

**Figure 40:**
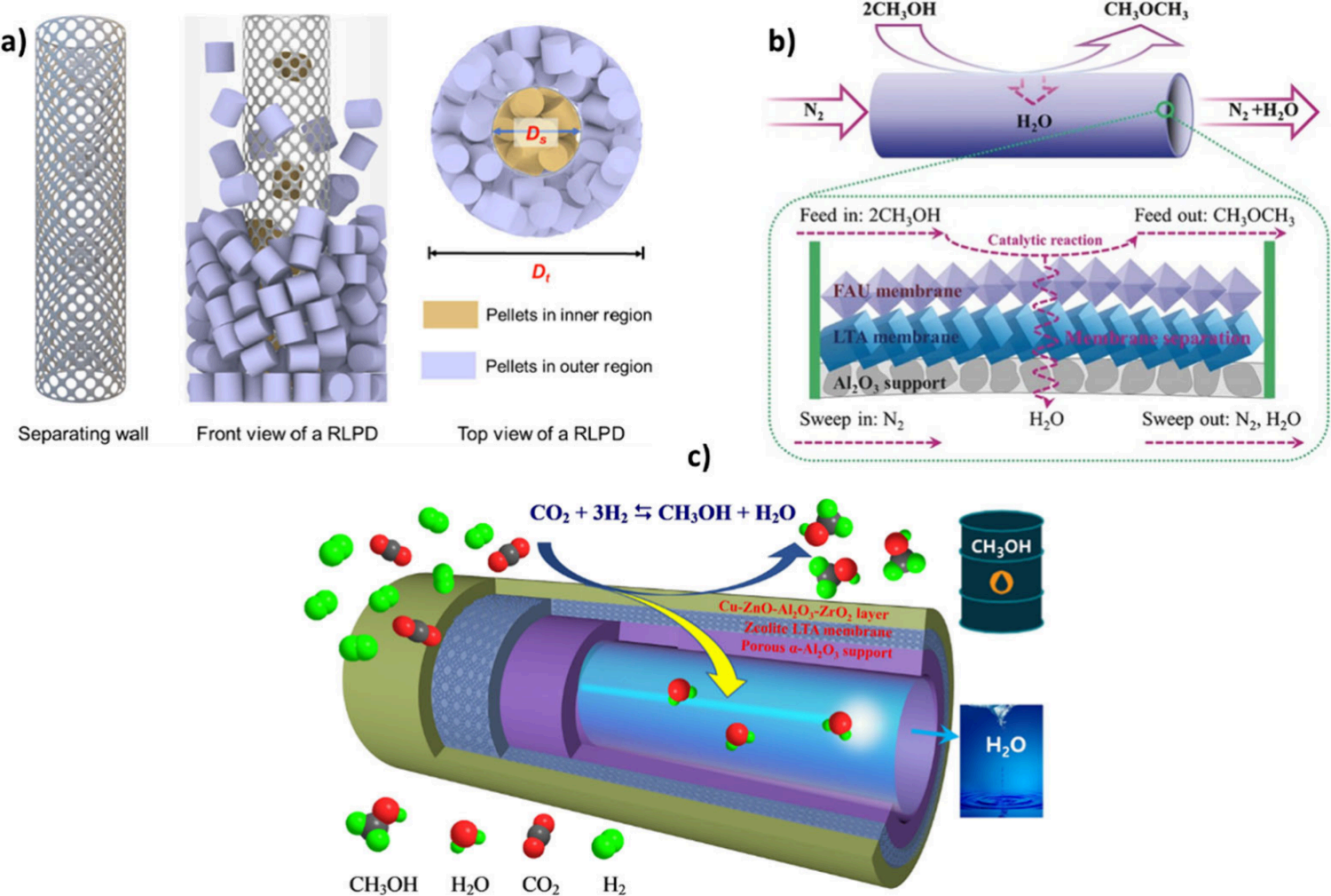
**a)** Schematic representation of radially layered packed bed reactor. Reproduced with permission from Weng et al.^352^ Copyright 2022, Elsevier. **b)** In situ water removal in a bifunctional catalytic membrane reactor based on a zeolite FAU-LTA double-layered membrane. Reproduced with permission from Zhou et al.^353^ Copyright 2016, John Wiley & Sons. **c)** In situ water removal in a bifunctional LTA@Cu-ZnO-Al_2_O_3_-ZrO_2_ catalytic membrane reactor. Reproduced with permission from Yue et al.^354^ Copyright 2021, John Wiley & Sons.

Another interesting intensification, recently introduced, is the concept of catalyst-specific heating and thermometry in tandem catalysis. This allows the individual temperature of two active catalysts combined in very close proximity to be controlled. Accordingly, Farpón et al.^355^ exploited localized magnetic induction heating in a radiofrequency oscillating field to induce a temperature gradient between two close active sites, which function at different temperature windows.

### 7.3 Complexities Associated with Catalyst Development and Reaction Engineering

In addition, it is crucial to obtain a comprehensive understanding of the interactions and relationships among various parameters to adjust catalyst properties and optimize an economically viable catalytic process.^356^ Exploiting in situ and operando techniques can be a helpful strategy to gain a deeper insight into the dynamic evolution of active phases, intermediate species, and particle structure, along with structure–activity relationships and different phenomena during reaction.^357–360^ In addition, examining catalytic reactions from various aspects through a combination of multiple in situ characterization methods can yield valuable insights into the chemistry of catalyst materials, the mechanisms of catalytic reactions, and the identification of active sites.^361–363^ These techniques enable continuous observation of the phenomena during the reaction, providing real-time monitoring and offering valuable information about the interaction, migration, and behavior of active sites on catalysts assembled via various integration schemes.

The kinetics of CO_2_ hydrogenation using Fe-oxide/zeolite tandem catalysts has not yet been fully understood, mainly due to the involvement of a complex surface reaction network.^364^ Traditional computational methods, such as density functional theory (DFT), microkinetic modeling (MKM), and kinetic Monte Carlo (KMC) simulations, have been extensively employed to investigate reaction mechanisms through the identification of active sites, rate-determining steps, and reaction pathways. These methods have some limitations, despite providing valuable atomic-level insights. For instance, DFT cannot precisely model complex reaction networks, which include long-range interactions and competing pathways. In addition, extensive input parameters and assumptions required for KMC and MKM methods hamper obtaining the complex reaction network, especially when heavy hydrocarbons form.^365–367^

However, AI and ML utilizing the data from experiments and computations have gained much attention as emerging tools to overcome these barriers and complement traditional methods.^368–370^ Exploiting both computational and experimental data from the atomic to laboratory scale (Figure 41(a)), ML models are able to indicate linear/nonlinear relationships, predict catalyst activity, and explore chemical spaces, which would be infeasible with time-consuming and computationally demanding ab initio calculations.^371–374^ Moreover, such AI/ML approaches have started to provide a deeper insight into reaction mechanisms and the discovery of new catalysts through data integration across various lengths and time scales.^375–378^ After finding stable catalyst structures under reaction conditions, mechanistic analysis and microkinetic simulations can be employed to gain deeper insights into catalyst design. These predictions can be validated through the synthesis, characterization, and testing of the catalysts (Figure 41 (b)).^374^ Combining ML and first-principle calculations, Wang et al.^379^ elucidated the influential factors in CO_2_ reduction by Cu-based single-atom alloys. Low generalized coordination numbers and valence electron numbers were identified as key descriptors for determining catalytic performance. It was demonstrated that the ML model could generalize among different alloying elements. In addition, using electronic structure calculations, it was revealed that CO adsorption was enhanced on the negative centers of the surface. Finally, they identified AgCu, PdCu, and GaCu as promising catalysts for electrocatalytic CO_2_ reduction.^379^

**Figure 41:**
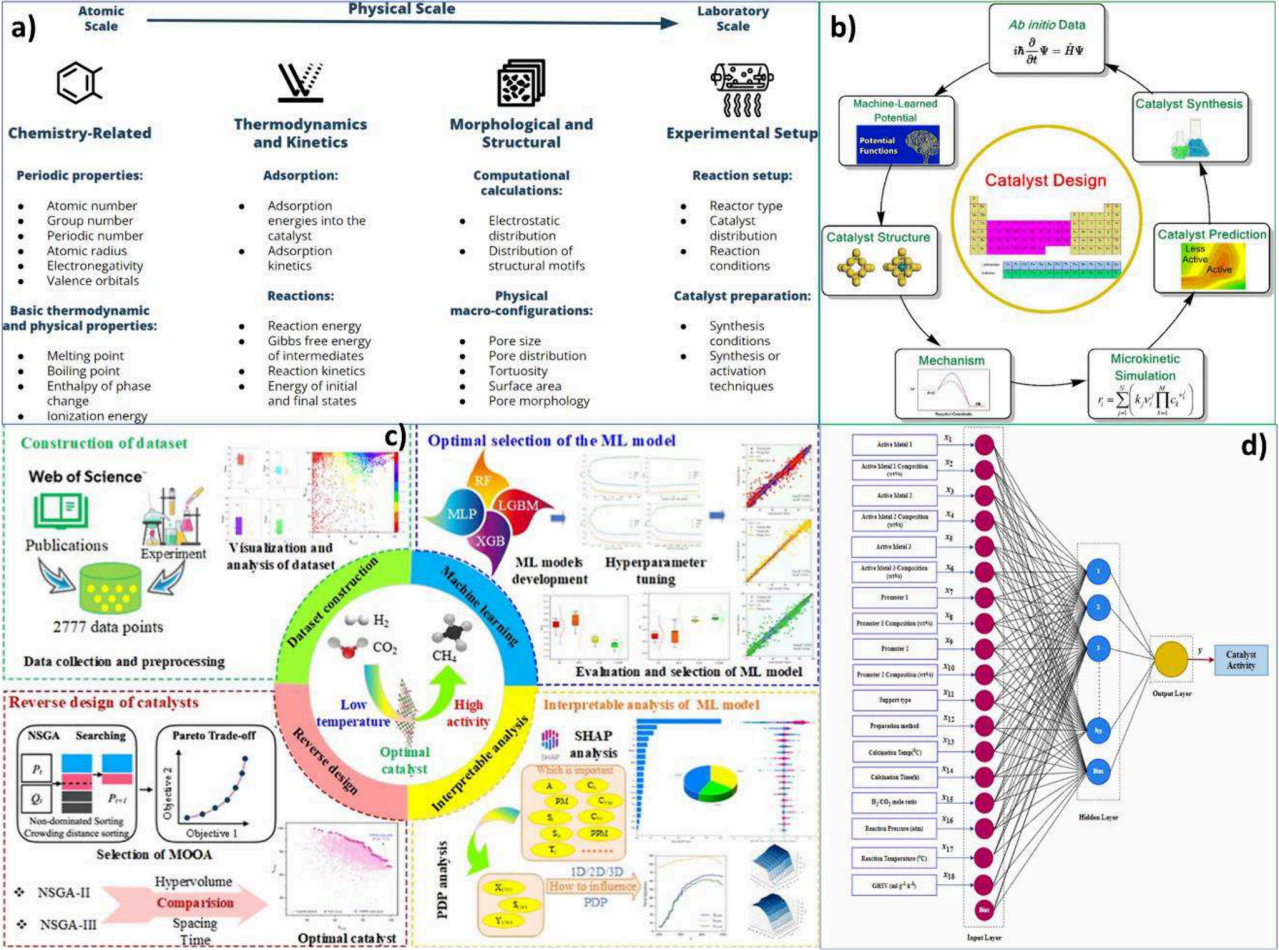
**a)** Descriptors used in ML for heterogeneous catalysis applications. Reproduced with permission from ref 371. Copyright 2024, ACS. **b)** Schematic illustration of ML potentials coupled with ab initio calculations in catalyst design. Reproduced with permission from ref 374. Copyright 2018, the John Wiley & Sons. **c)** Schematic demonstrating ML workflow for low temperature CO_2_ methanation. Reproduced with permission from ref 386. Copyright 2024, ACS. **d)** Proposed single hidden-layer ANN model for prediction of catalyst activity in CO_2_ hydrogenation to light olefins. Reproduced with permission from ref 388. Copyright 2023, Elsevier.

In addition, understanding how a fixed bed reactor responds to changes in inlet conditions and disturbances, as well as the formation of hot-spots and spatiotemporal patterns, is crucial. The advanced capabilities of CFD, combined with AI/ML models that can handle complex bed geometries and simulate realistic flow fields, enable the connection of these factors to the specific responses of catalyst pellets in a three-dimensional flow environment.^380–382^ Thus, the development of advanced physics- and chemistry-informed AI/ML models based on data generated from multiscale modeling will likely provide the fundamental insights required to design active and selective catalysts.^383–385^ For instance, Yang et al.^386^ used an ML framework to demonstrate the challenges in low-temperature (<250 °C) catalyst development for CO_2_ methanation. By integrating multiobjective optimization and interpretability analysis with ML, the relationships between catalyst preparation, composition, and reaction parameters were revealed. The active component content and calcination temperature were identified as key descriptors of catalyst performance using the light gradient boosting machine (LGBM) model. In addition, Ru-Ba/Cr_2_O_3_-SrO was proposed as a new high-performance catalyst at low temperatures (Figure 41 (c)).^386^ A recent artificial neural networks (ANNs) based ML analysis predicted that tailoring product distributions based on specific selectivity or conversion for optimization purposes is achievable for CO_2_ conversion to CO, CH_4_, olefin, and paraffin, with carbon numbers varying from 2 to 10 via FT synthesis.^387^ Using structural, composition, and operating parameters, Chandana et al.^388^ developed an ML framework to model and design catalysts for CO_2_ hydrogenation to light olefins. It was found that ANN models trained using the Levenberg–Marquardt and Bayesian–regularization algorithms showed better predictions of CO_2_ hydrogenation performance. They also employed the AI-based MOO technique for integrated catalyst design and operating condition optimization (Figure 41 (d)).^388^ In another study, Yang et al.^158^ created a database using literature data on CO_2_ hydrogenation to heavy hydrocarbons to determine the influential descriptors via statistical analysis and regression trees. It was found that pressure, temperature, and catalyst treatment played crucial roles in determining CO_2_ conversion and C_2+_ selectivity. In addition, the Pauling electronegativity of dopants was found to be one of the most essential descriptors affecting both activity and selectivity. Results revealed that Fe-based catalysts promoted with Mn/K could enhance the CO_2_ hydrogenation performance effectively.^158^

The combination of in situ characterization data and multiscale modeling holds the potential for a methodical exploration of how the proximity of active sites influences the distribution of resulting hydrocarbons in the context of CO_2_ hydrogenation. As a whole, multiscale modeling, which involves simulating catalytic processes across different lengths and time scales using computational methods, allows for investigating the behavior of active sites at different levels, from molecular to macroscopic.^389,390^ Thus, by incorporating fundamental concepts of reaction kinetics, thermodynamics, and heat/mass transport, the interaction of active sites based on their proximity and spatial arrangement can be predicted using multiscale modeling. Figure 42 illustrates the primary relationship between reactor operation and catalyst properties, which can be combined to understand the engineering feasibility of the CO_2_ hydrogenation process for fuel production.

**Figure 42:**
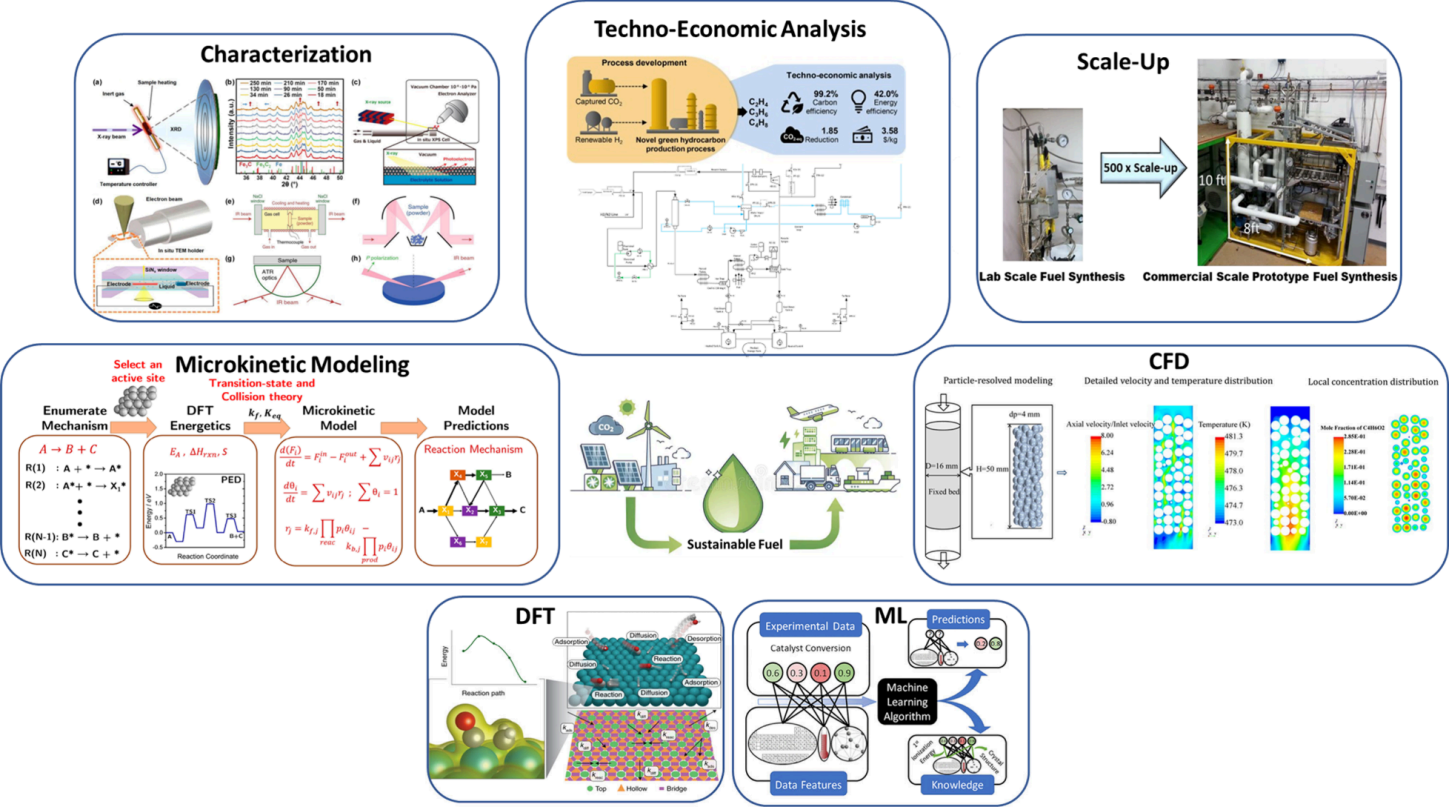
Key techniques and methods required for analyzing and developing the CO_2_ hydrogenation into value-added products. Reproduced with permission from references Characterization,^24^ Mikrokinetic modeling,^365^ DFT,^391^ ML,^392^ CFD,^393^ Scale-up,^394^ and techno-economic analysis.^395^ Copyright permission obtained from Elsevier 2023; ACS 2020; Nature Catalysis 2019; ACS 2019; Elsevier 2019; MDPI 2020; Elsevier 2020.

To date, most scale-up investigations have focused on CO, CH_4_, and methanol production. However, studies on the feasibility of fuel production via coupling RWGS and FT route, which is more challenging, are in the early stage.^396–398^ Recently, a ton-scale gasoline production plant in the chemical industrial park of Shandong province in China was reported.^46^ Nevertheless, the industrialization of this process requires holistic efforts and investigations that take into account economic issues.^204,399,400^ Indeed, large-scale CO_2_ hydrogenation requires a critical analysis of energy requirements and environmental impacts.^401–403^ CO_2_ hydrogenation using energy derived from fossil fuels cannot mitigate CO_2_ emissions. Moreover, the cost of H_2_ production, CO_2_ separation from flue gas, and the separation of hydrogenation products must be considered for industrial exploitation of this concept.^404–406^

### 7.4 Proximity Determination from Lab- to Large-Scale Fixed-Bed Reactors

Traditionally, a packed bed reactor has been viewed as consisting of two separate domains: the macroscopic level, which pertains to phenomena within the bed dimensions, and the microscopic level, which focuses on phenomena within individual particles.^407,408^ The architectural configuration of a catalytic reactor plays a crucial role in chemical processes, particularly when transitioning from small-scale laboratory setups to larger industrial reactors. The distance and organization of catalyst particles become critical factors affecting parameters such as bed permeability, reaction kinetics, and heat/mass transfer rates. Understanding the spacing between particles helps refine reactor designs to enhance heat/mass transfer efficiency. The interaction among heat/mass transfer properties, fluid dynamics, and reactor structure is pivotal in tuning the performance of larger reactors.^409,410^

#### 7.4.1 Determining the Proximity at Lab-Scale

Catalyst particles contain active sites that are typically sized in nanometers or angstroms and prone to spatial variations. The activity of one site within the catalyst grain can differ from that of another due to localized chemical changes or variations in reactant accessibility induced by shape-selective effects. Additionally, the quantity of active sites varies across surfaces, with supported metal nanoparticles exhibiting adjustable reactivity and selectivity based on their shape, which affects the distribution of atoms at edges and corners. High-index planes typically have higher concentrations of unsaturated atomic steps, edges, and kinks, which serve as active sites for catalytic reactions. These nanoparticles vary widely in size and shape, each possessing unique catalytic properties. Adding other elements can introduce heterogeneity, resulting in uniform or egg-shell distributions within the nanoparticle.^408^ Identifying proximity at the laboratory scale requires data extracted from high-resolution imaging methods, utilizing a suitable distribution function across different proximity metrics, and pinpointing the distance. Measuring the distance between active sites in heterogeneous catalysis typically requires techniques with high spatial resolution that can directly image or probe individual active sites.^411,412^ Although numerous experimental techniques offer valuable insights into the spatial arrangement of active sites in catalysis, only a few can directly measure the distance separating them.^413^

##### 7.4.1.1 Technical Methods and Their Limitations

Over the past decade, efforts have been made to integrate multiple spectroscopic methods into a single setup, enabling correlative spectroscopy on the same catalyst material under uniform conditions. This eliminates the necessity for sample transfer and allows for the collection of complementary structural, electronic, and kinetic data regarding a catalytic process.^408^ In recent years, the array of techniques for chemical imaging of catalyst particles has widened and continues to grow with the emergence of new promising methods. These approaches offer enhanced spatial and temporal resolution, along with enhanced sensitivity and chemical information content. Figure 43 illustrates the present and emerging (italicized) imaging methodologies for studying catalytic materials at individual particle sizes, with the capacity to reveal novel properties of catalytic materials at the micro- and nanometer scales.^414^

**Figure 43:**
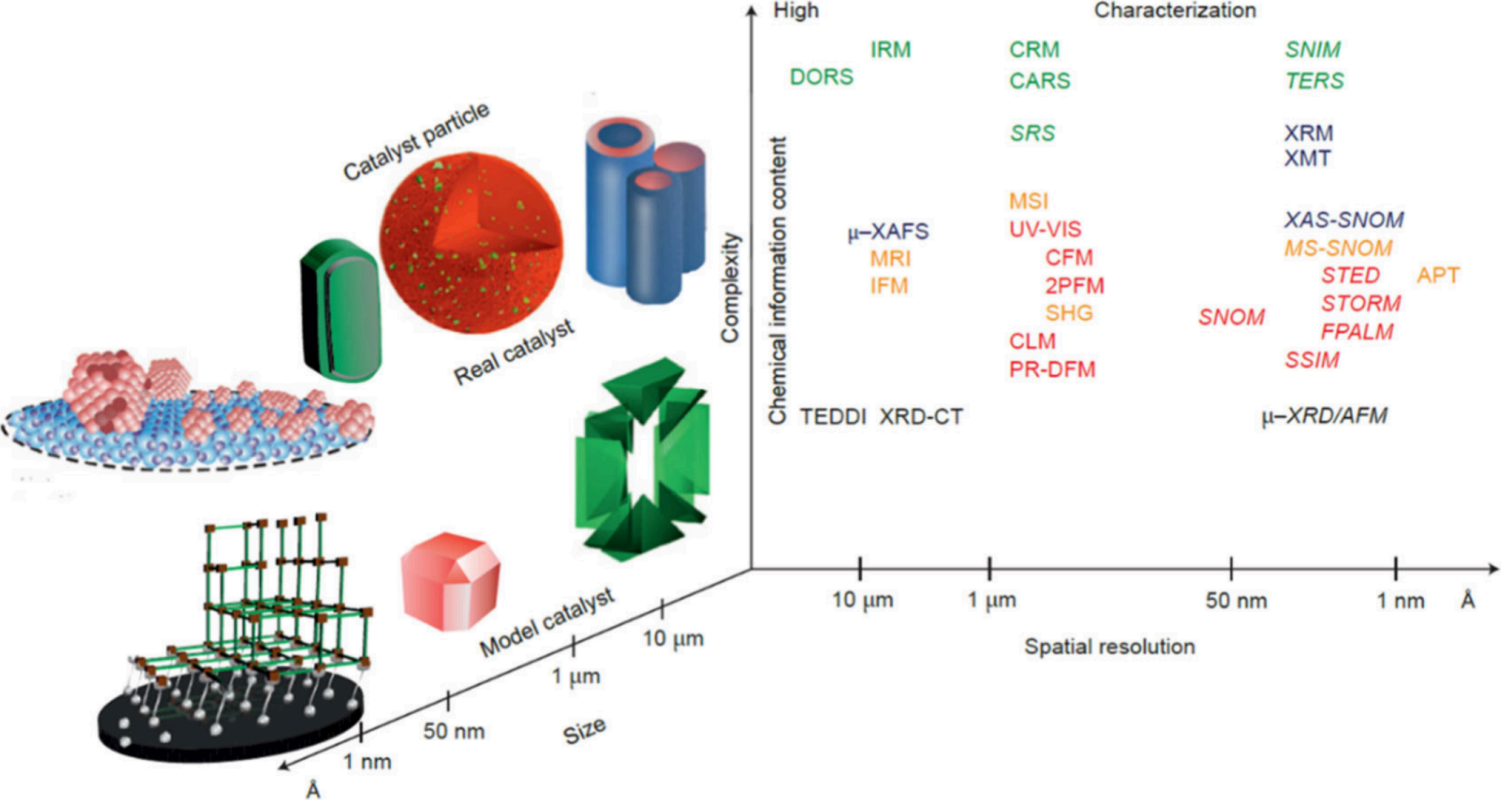
Chemical imaging methods currently available, along with those anticipated in the future (in italics), for studying individual catalyst particles. Reproduced with permission from ref 414. Copyright 2012, Nature Chemistry.

While robust methods are employed in materials science and catalysis studies, especially for investigating nanostructured materials and surfaces, they are not typically utilized for directly determining distances between active sites. Instead, they offer high-resolution imaging and spectroscopic data, enabling researchers to visualize the spatial arrangement of elements, chemical species, and morphological characteristics at the nanoscale.^414,415^ Techniques such as high-resolution electron microscopy or scanning probe microscopy are commonly used to directly measure distances between active sites in heterogeneous catalysis.^415^ Determining the proximity of active sites in heterogeneous catalysts involves various technical methods, such as scanning tunneling microscopy (STM),^416^ atomic force microscopy (AFM),^417^ transmission electron microscopy (TEM),^418^ annular dark-field scanning transmission electron microscopy (ADF-STEM),^419^ atom probe tomography (APT),^420,421^ and Förster resonance energy transfer (FRET).^422,423^ Each technique has its own set of advantages and limitations, as illustrated in Figure 44. These techniques provide direct insights into the spatial arrangement of active sites in heterogeneous catalysis, enabling researchers to better understand their behavior and optimize catalytic performance.

**Figure 44:**
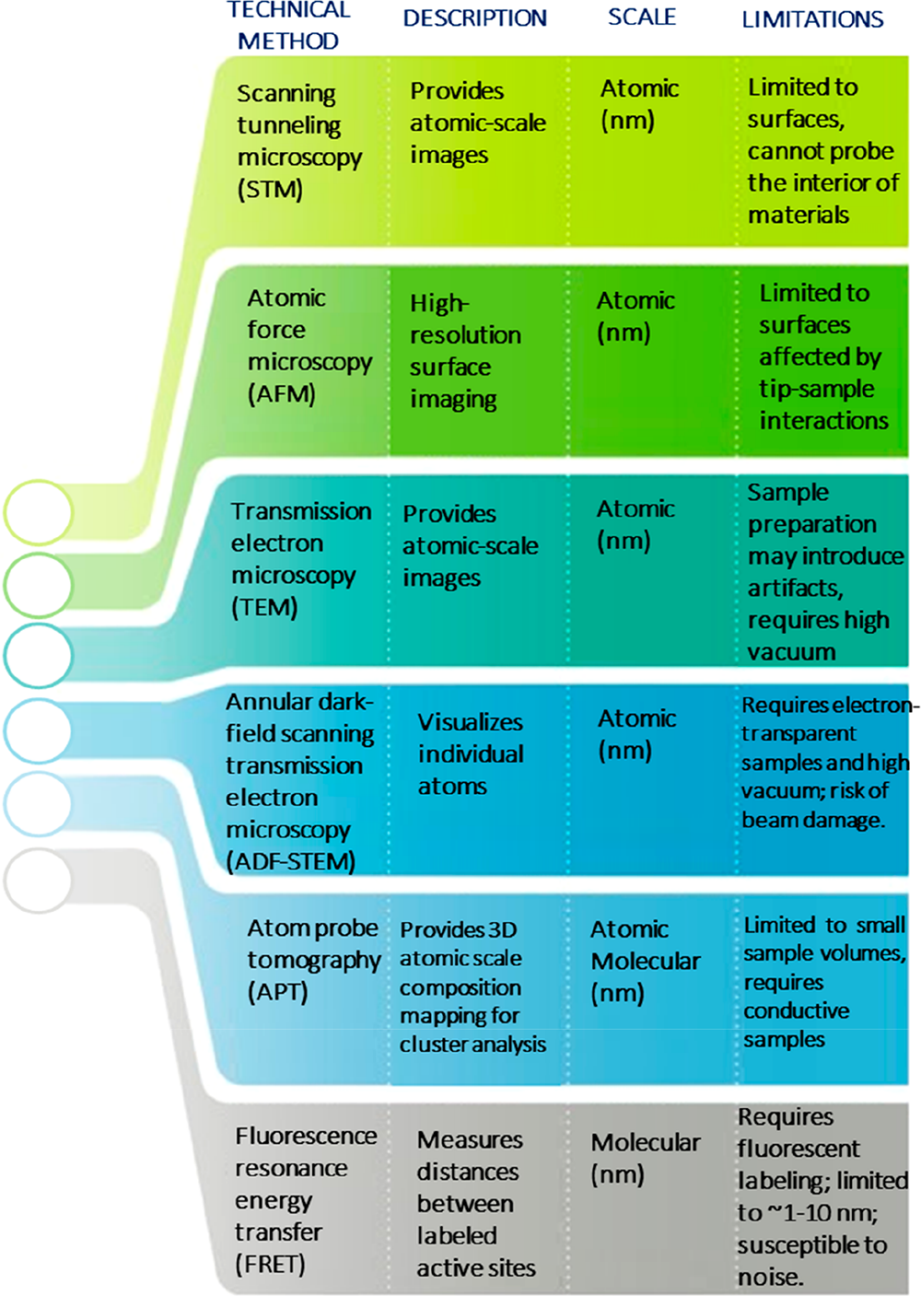
Schematic representation of common techniques to probe the spatial arrangement and distances of active sites (Designed by authors).

In brief, though there are diverse technical approaches for assessing the proximity of active sites in heterogeneous catalysts, each method has its own limitations. Therefore, researchers combine multiple techniques to address these limitations and achieve a thorough comprehension of catalyst structure and functionality.^415^

##### 7.4.1.2 Common Proximity Metrics in Heterogeneous Catalysis

In heterogeneous catalysis, the spatial arrangement of active sites has a significant influence on reaction pathways, selectivity, and the overall effectiveness of the catalyst. As described in section 6, understanding and controlling proximity effects are crucial for designing advanced catalysts that convert CO_2_ into valuable long-chain hydrocarbons.^136^

Proximity metrics, which quantify intersite distances and spatial distributions, help evaluate how active site positioning affects reaction kinetics, mass transport, and product formation. Therefore, researchers can develop more efficient and commercially competitive catalytic processes, via characterizing and optimizing the active site proximities.^424^ Additionally, these metrics, along with other critical parameters, play a significant role in connecting lab-scale research with industrial-scale catalytic applications. Optimizing proximity metrics can be considered an important factor for improving catalyst performance and contributing to potential economic viability or industrialization.

Various proximity metrics can be employed to determine the spatial arrangement of active sites in catalysts. These metrics, derived from mathematical and statistical geometry methods that describe distances and complex spatial relationships among points, include interparticle distance, average nearest-neighbor distance, cluster size distribution, spatial correlation function, Voronoi tessellation, pair distribution function, and spatial autocorrelation (e.g., Moran’s I and Geary’s C). It should be highlighted that spatial autocorrelation is more commonly exploited in geography, ecology, etc., for analyzing spatial patterns. Spatial autocorrelation can be applied to catalysts in principle, but their use in quantifying active sites in heterogeneous tandem catalysts may require method adaptation. The most important and commonly used of these proximity metrics are explained below:

Interparticle Distance: Measured using the Euclidean distance function, which calculates the straight-line distance between two points in space.
Average Nearest-Neighbor Distance: Defined as the mean distance between each point and its nearest neighbor, providing insight into the dispersion of particles.
Voronoi Tessellation: Analyzed using the Voronoi tessellation algorithm, which partitions space into regions based on the distance to a specific set of points.
Pair Distribution Function: Determined via the radial distribution function, describing how particle density varies as a function of distance from a reference particle.

These abovementioned proximity metrics have been widely applied in various fields, such as biology, materials science, geographic information systems (GIS).^425–430^ Despite its critical importance, the quantitative analysis of active site spatial arrangements in heterogeneous tandem catalysis remains a significant challenge and a major focus of nowadays research. We propose extending their use further in the field of catalysis to determine the distances and complex spatial relationships between active sites. Figure 45 presents various proximity metrics used to describe the spatial arrangement of active sites within catalysts as a case study.^78^

**Figure 45:**
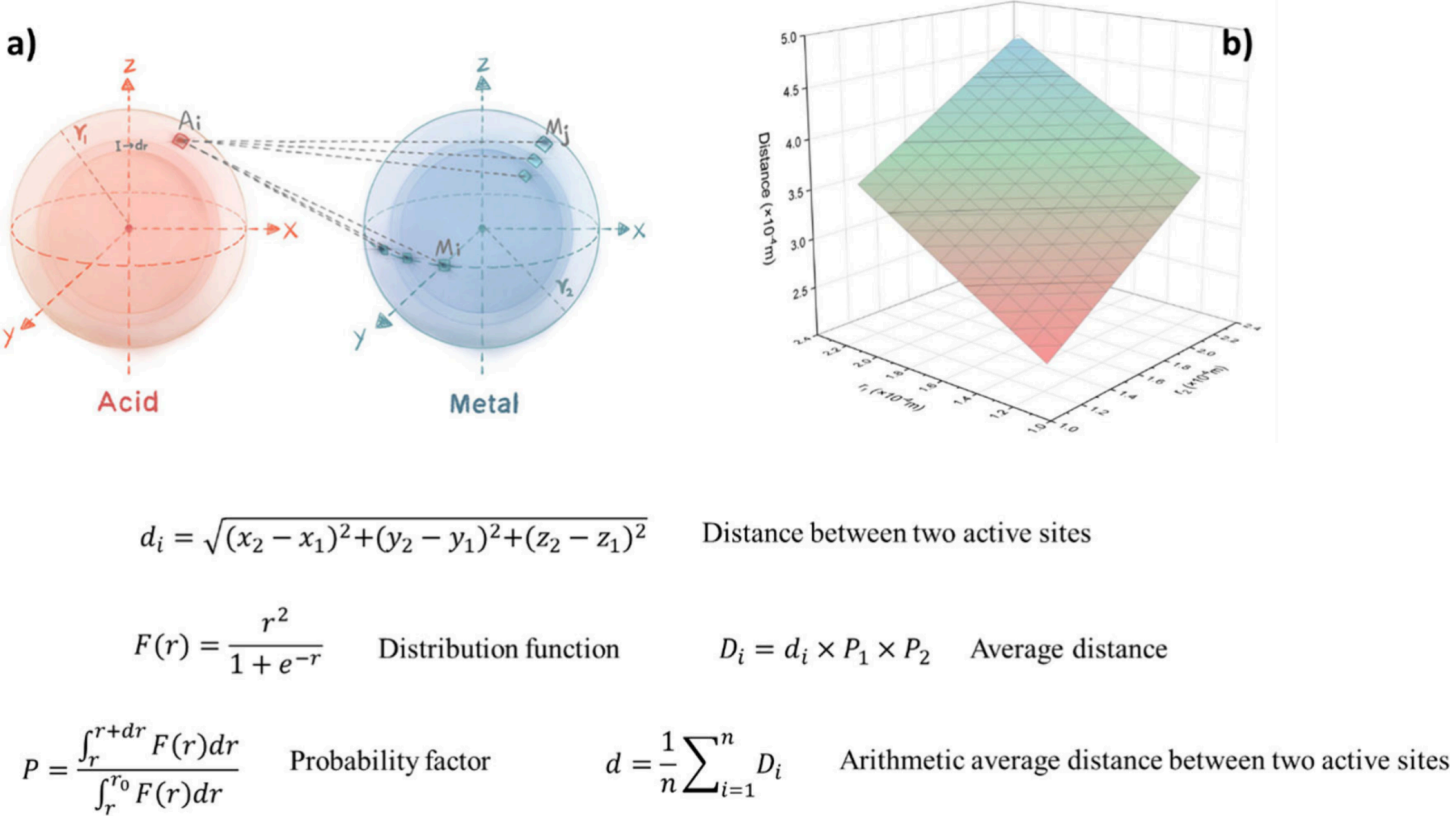
**a)** Scheme of the iterative method for distance estimation between metal oxide and zeolite. **b)** The *x*-axis, *y*-axis, and *z*-axis directions represent the radius of the zeolite particles, the radius of the oxide particles, and the average distance (*d*) between the active sites of the two catalyst particles, while *d* = *f* (*r*_1_, *r*_2_). Reproduced with permission from ref 78. Copyright 2022, ACS.

By characterizing these parameters, researchers can gain a comprehensive understanding of proximity metrics in heterogeneous catalysts, facilitating the rational design of catalysts and reactor setups to optimize catalytic performance. For example, by utilizing distribution functions (DFs), the proximity of active sites within heterogeneous catalyst materials can be quantitatively evaluated, providing valuable insights for catalyst design and enhancement.^431,432^ Initially, data should be gathered using high-resolution microscopy imaging techniques to pinpoint individual nanoparticles that act as active sites. Subsequently, the DF is computed to depict the likelihood of locating an active site at a specific distance from a reference point. Evaluation of the resultant DF demonstrates the dispersion of distances between active sites, where a uniform DF indicates an even distribution, while peaks show clustering or aggregation. Analysis of the DF helps in determining the proximity of active sites, which can be correlated with the catalyst performance.

Recently, an iterative method to estimate the distance between two functional components (metal and acid site) integrated via the granule-stacking method was proposed for syngas conversion.^78^ Using MATLAB, the authors modeled two catalyst granules as ideal spheres with homogeneous distributions of active sites. They established a spherical model of the catalyst particles by layering spherical shells with decreasing radii from an initial radius (*r*_0_) to 0. A probability function was assumed for the number of active sites in each layer, resulting in distribution functions of active sites with different radii. The distance between each active site was calculated, and a normalization process was applied to the distribution function. The average distance from all units at a given radius (*r*) to each point of the other sphere is computed, yielding an average distance (*d*). By analyzing a large number of scatter points (*r*_1_ (oxide), *r*_2_ (zeolite), *d*), a relationship between the distance and catalyst particle size (*d* = *f*(*r*_1_, *r*_2_)) could be derived and matched to practical catalyst systems. The schematic representation of the method is illustrated in Figure 45.

In essence, although the details of the analysis may differ based on the proximity metric under study, the overarching process entails imaging the catalyst material, pinpointing active sites, measuring distances or associations among them, and scrutinizing the acquired data to quantify proximity metrics and grasp their significance for catalytic performance.

#### 7.4.2 Adjustment of Spatial Arrangement at Large-Scale Reactors

The connection between proximity metrics, which refer to the spatial arrangement of active sites in heterogeneous catalysts, and the industrialization of chemical processes is vital. It shows the significance of fundamental catalyst characterization and reactor design considerations in successfully translating lab-scale innovations into commercially viable chemical processes. Researchers can develop more efficient and sustainable processes that meet the demands of industrial-scale production by leveraging the insights into the active sites distribution.

The reactor design and spatial arrangement of catalyst particles are crucial in determining the heat/mass transfer limitations. The interparticle distance influences factors such as bed porosity, effective diffusivity, heat and mass transfer coefficients, and reaction rates implicitly.^433^ In fact, no explicit relationship has been provided for scaling up the reactor, considering the proximity between active sites due to nonidealities related to the large-scale reactors. Nonideal effects, such as channeling, dead zones, hot spots, axial and radial temperature gradients, pressure drop gradients, fouling and coking, wall effects, and heat/mass transfer limitations, can significantly impact the performance and efficiency of large-scale fixed-bed reactors.

In this regard, an indirect approach can be developed based on the analogies of dimensionless numbers (DNs) to ensure dynamics or flow characteristics (Reynolds), thermal (Nusselt and Péclet_h_ numbers), and mass transfer (Sherwood, Schmidt, Péclet_m_, and Damköhler numbers) similarities between scales.^434,435^ This can provide valuable insights into the design and operation of a packed bed reactor. In the packed bed reactors, using such analogies between lab-scale and large-scale reactors can be viable if the particle characteristics, flow regimes, and operating conditions are sufficiently similar between the two scales. In fact, the proximity of active sites can alter the DNs by affecting reaction rates and heat/mass transfer coefficients. DNs provide a tool to compare the relative importance of gravity, inertia, convection, diffusion, and reaction across different scales. For instance, similarity in Reynolds numbers indicates comparable flow patterns and turbulence levels, while similarity in Péclet numbers signifies equivalent rates of diffusive and convective transport. Figure 46 presents the most important DNs and their applications in fixed-bed reactor scale-up.

**Figure 46:**
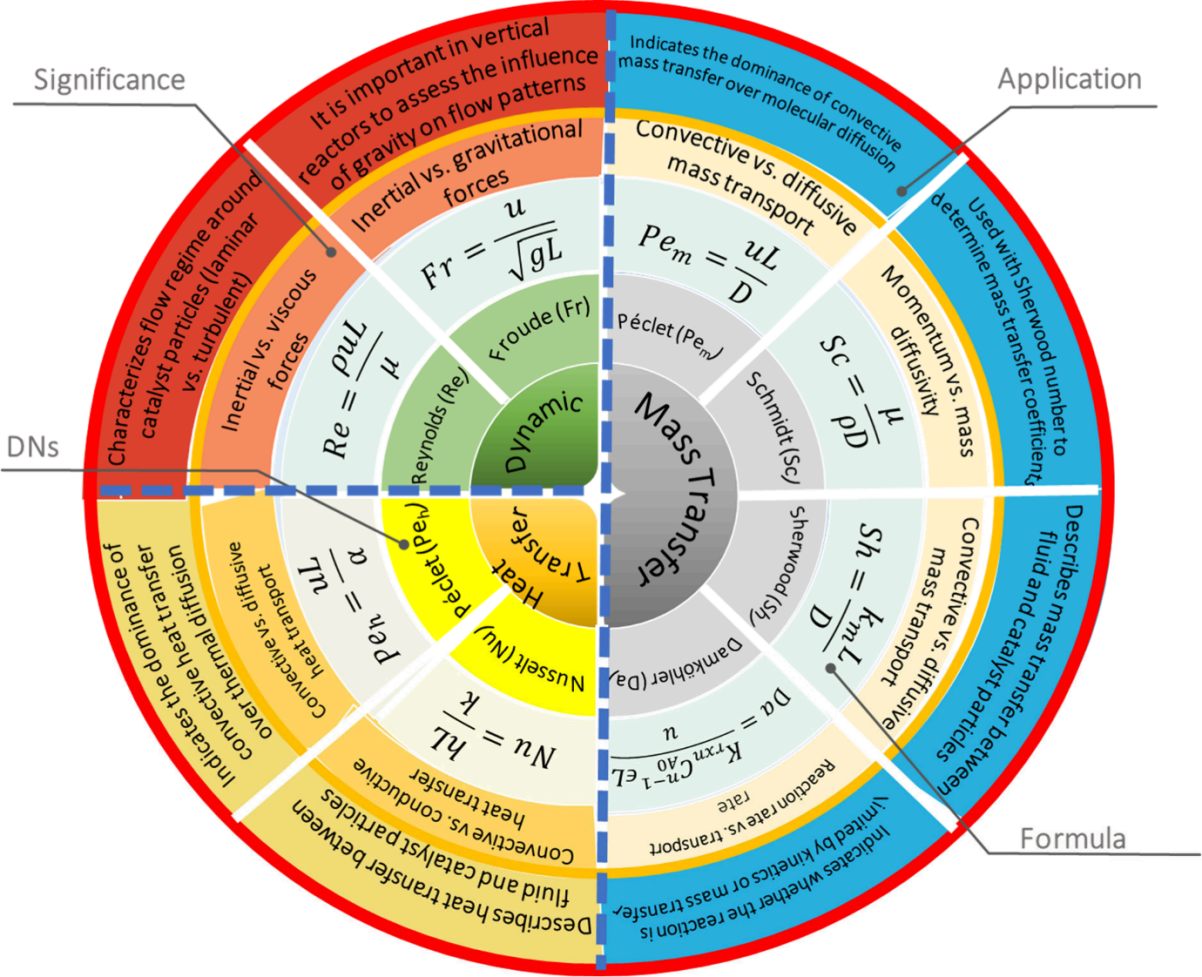
Significance and application of dimensionless numbers in the scale-up of fixed-bed reactors (Designed by authors).

Moreover, in chemical engineering and catalysis, the dimensionless parameter Da describes the relative significance of mass transfer and chemical reaction rates in a system. It is a ratio of the reaction rate to the mass transfer rate by diffusion. Where the Da is less than 1, scaling up processes governed by reaction-control can be achieved relatively straightforwardly by optimizing reactor design to ensure efficient heat transfer and mixing. However, additional factors must be considered when the Da exceeds 1 in diffusion-controlled regimes to overcome mass transfer constraints. The reaction occurs faster than mass transport, resulting in concentration gradients within the system. Typically, these gradients negatively impact the optimal performance of reactors and can affect the overall selectivity of the reaction (Figure 47). For example, mixing plays a significant role in affecting a competitive, consecutive side reaction where A + B → C and C + B → S (Da > 1). A and B react before homogeneity, while C, in the presence of B, results in the formation of the side-product, S. This could require enhancing mixing, modifying the catalyst structure, or adapting the reactor setup.^436^

**Figure 47:**
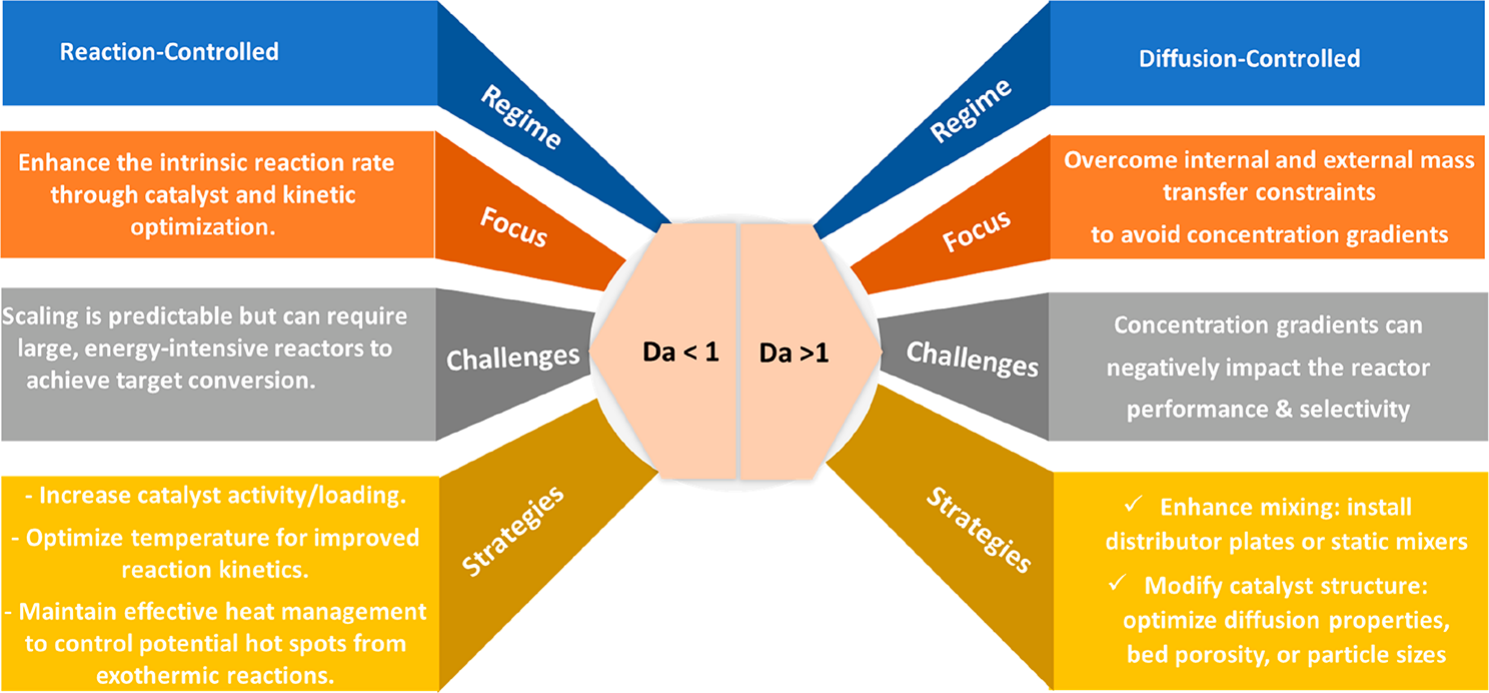
Implications of the Damköhler number (Da) in reactor scale-up (Designed by authors).

To take advantage of DNs, the arrangement of catalysts at the lab-scale should be optimized for the desired distribution of products. In the next step, the reaction rates and other parameters required to estimate DNs (such as diffusion coefficients, density, velocity, porosity, etc.) should be calculated. Using the lab-scale reactor data, including DNs and reaction rates, the AI/ML model should be trained to identify the correlation between active site proximity and reaction rates in response to changes in catalyst arrangement and DNs. Therefore, the optimized proximity that can result in the highest performance can be identified by the model. In the next step, we can use CFD simulations to calculate the concentration, temperature, and pressure profiles in the reactor. By integrating ML with CFD, the optimal reaction conditions can be determined. We can also integrate both microscale MD and macro-scale CFD to tune the catalyst and bed design for optimized proximity. Then, we can use the trained AI/ML model from lab-scale to predict the performance under large-scale conditions. By exploiting the AI/ML-CFD, the behavior of a large-scale reactor can be simulated, ensuring similar DNs to those calculated from the lab scale. Comparing the reaction rates and DNs in both lab-scale and large-scale reactors provides a comprehensive insight into the differences in reaction efficiency, flow regimes, and heat dissipation. This process should be repeated for the large-scale reactor by changing the catalyst particle arrangements to approach the ideal DNs achieved in the lab-scale reactor. Adjusting the active site proximity and catalyst arrangement in large-scale reactors involves a combination of strategies such as structured packing, optimized particle size, graded beds, and layered and segmented beds. The whole explained process is demonstrated in Figure 48.

**Figure 48:**
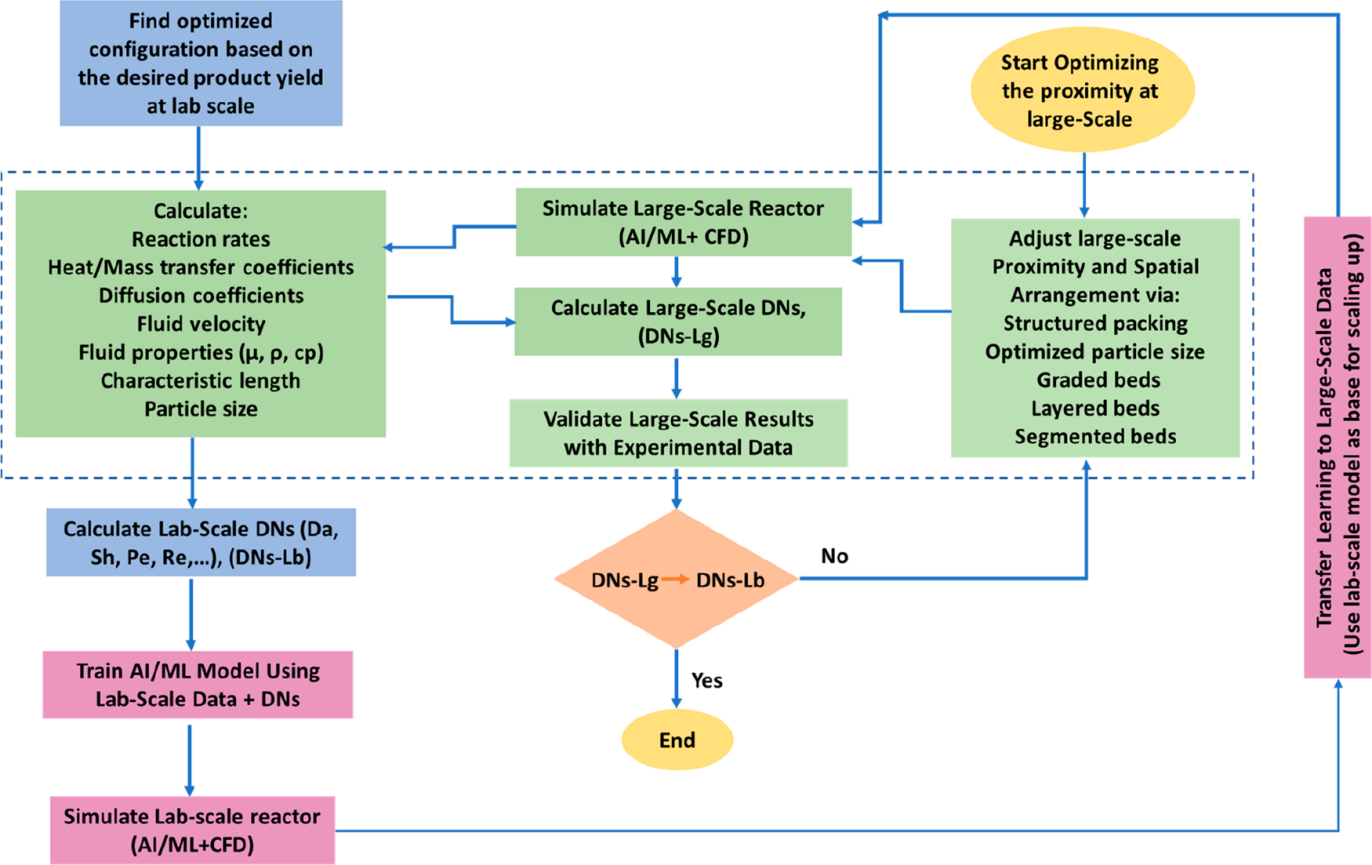
Flowchart showing how using dimensionless numbers (DNs) can help adjusting catalyst arrangement at a large scale by training the AI model with lab-scale data (Designed by authors).

Based on the chemical information and the spatial resolution needed for the catalyst material, an appropriate characterization technique can be chosen to assess spatial and temporal heterogeneities. This applies to model catalyst particles, such as large zeolite crystals and metal particles, as well as industrial catalysts, including small zeolite crystals, catalyst bodies, and supported metal nanoparticles.^414^ However, it is essential to recognize that these methods frequently simplify complex phenomena, disregarding aspects such as nonideal flow or catalyst deactivation. Additionally, they rely on empirical correlations that may not be transferable. To minimize nonideal effects in large-scale fixed-bed reactors, it is essential to combine accurate design, precise operational practices, and advanced modeling techniques. Using advanced catalyst materials, achieving uniform catalyst packing, optimizing flow distribution, and effective heat management are crucial factors for consistent and efficient reactor performance. Moreover, the reliability and scalability of the reactor system can be enhanced through regular monitoring, maintenance, and validation via pilot testing and computational simulations. Nonetheless, AI/ML can automate the entire iterative process of catalyst arrangement optimization for various reactor configurations, enabling faster adjustments as conditions evolve, particularly in large-scale reactors.

## 8 Concluding Remarks and Future Directions

Transforming CO_2_ into valuable chemicals and fuels using renewable energy is an emerging concept for developing carbon-neutral energy and fuel production technologies. In recent decades, thermo-catalytic CO_2_ hydrogenation for producing value-added chemicals and fuels using heterogeneous catalysis has emerged as a promising method, attracting significant attention worldwide. Particularly noteworthy is the successful development of active catalysts comprising Fe-based oxide and zeolite, which have obtained considerable attention in producing hydrocarbons within the fuel range (C_5+_). Despite extensive investigation in this field, there are still unsolved issues regarding the catalyst design for the industrialization of CO_2_ hydrogenation to value-added products.

I) It was demonstrated that the integration schemes of the basic sites of Fe-based oxide and zeolite acidic sites could considerably affect the reaction performance. In addition, the selection of promoter and other additional elements, as well as the loading and method of their introduction to either the Fe-oxide or zeolite (impregnation, one-pot, or physical mixing) alter the distribution of products in CO_2_ conversion. It was revealed that by selecting an appropriate alkali promoter in Fe-based oxide, along with an optimized integration scheme with zeolite, it is possible to tune the heavy hydrocarbon distribution and the Aro/N-Aro ratio. Moreover, the topology and BAS of zeolite, along with its modification via surface neutralization or elemental substitution, are among the influential factors affecting the proximity of active sites and, consequently, the product distribution.
II) Furthermore, the reaction sequence, density of acid/base active species, and architecture of pores/cavities were found to be the most important features contributing to the proximity of active sites. To this end, encapsulated catalysts are promising alternatives to conventional catalysts, as they enhance both diffusion and confinement effects; however, further investigations are needed for industrialization applications. Moreover, practical control of the proximity of active sites can be tuned by altering the synthesis methods. In this regard, adjusting the proximity and ratio of Fe-oxide and Fe-carbide species via the pyrolysis of organic precursors and MOFs, where Fe and C can be present in close proximity, has attracted significant attention. However, dispersing the promoted Fe-oxide over mesoporous supports resulted in increased distance between metallic active sites and BAS of the zeolite, which necessitates closer proximity between oxide and zeolite.
III) It is noteworthy that although both Na and K belong to the alkali group, their reduction abilities are found to be different as a result of inherent differences in their electronegativities and ionic radii. This led to different basic properties, and hence, the extent of electronic modification of catalysts differs. While Na can hinder all the reduction steps, from Fe_2_O_3_ to metallic Fe, K can only hinder the first hydrogenation step. In the remaining steps, the reduction is much easier, resulting in a more basic nature of K. On the other hand, Na could easily diffuse into the bulk of iron oxide due to its smaller size, while K mainly remains on the surface, increasing the electron density and facilitating electron donation and the formation of iron carbides. Nevertheless, the higher hydrogenation ability of K may be responsible for the formation of more CH_4_, along with more saturated and nonaromatic hydrocarbons rather than aromatics, on K-promoted Fe-based catalysts. Considering the above-mentioned differences, along with the influence of the proximity of active sites, it can be concluded that increasing the distance between the alkali-promoted Fe-based oxide and zeolite reduces the hydrogenation ability of K and results in an increased Aro/N-Aro ratio. However, hydrocarbons over Na-promoted Fe-oxide easily undergo hydrogenation reactions rather than aromatization, which leads to a decreased Aro/N-Aro ratio. Nevertheless, many other parameters, such as the second metal/promoter loading/doping, the loading of zeolite, and different surface modifications to tune its acid strength and density in zeolite, as well as the GHSV, must be considered when comparing the performance of various catalysts. In addition, to be industrially feasible and gain high C_5+_ STY, catalysts that maintain high performance over a longer period at increased GHSV must be developed.
IV) Experimental methods of activity measurements and in situ characterization need to be integrated with computational methods (DFT, molecular dynamics, microkinetic modeling, etc.) and ML to obtain a deeper insight into the active phases, their interactions, and evolution during reaction, as well as intermediate formation and their transfer to zeolite pores. Furthermore, CFD simulations and ML, which have gained widespread attention, should be exploited for catalyst design and process intensification to achieve the large-scale production of heavy hydrocarbons.
V) Moreover, to determine the spatial distribution of active sites, advanced spectroscopic techniques, combined with the use of appropriate proximity metrics, are necessary. Therefore, advanced imaging techniques and appropriate distribution functions are required. However, extending the lab-based results to a large scale requires unifying metrics such as dimensionless numbers, which can provide valuable insights about the phenomena associated with the reaction at different scales and could be optimized via an AI-enhanced iterative workflow.

The obtained results expand our knowledge about the role of integration methods that affect the proximity of Fe-based oxide and zeolite for C_5+_ hydrocarbon production, which will be helpful in designing the next generation of efficient catalysts for CO_2_ hydrogenation to C_5+_ hydrocarbons at industrial scales.

## Nomenclature

**Abbreviations**
FTS: Fischer–Tropsch synthesis
RWGS: Reverse water–gas shift
MFTS: Modified Fischer–Tropsch synthesis
G.S.: Granule stacking
D.B.: Dual-bed
D.R.: Dual reactor
M.G.: Mixed-powder granules
HSG: Honeycomb-structured graphene
BAS: Brønsted acid sites
LAS: Lewis acid sites
BTEX: Benzene, toluene, ethylbenzene, and xylene
BTX: Benzene, toluene, and xylene
PX: Para-xylene
DFT: Density functional theory
XPS: X-ray photoelectron spectroscopy
XRD: X-ray diffraction
TPD: Temperature-programmed desorption
STY: Space–time–yield
HRTEM: High-resolution transmission electron microscopy
BET: Brunauer–Emmett–Teller
SEM: Scanning electron microscopy
EELS: Electron energy loss spectroscopy
STEM: Scanning transmission electron microscopy
EDX: Energy-dispersive X-ray spectroscopy
DRIFT: Diffuse reflectance infrared Fourier transform
SVUV-PIMS: Synchrotron vacuum ultraviolet radiation-photoionization mass spectrometry
GHSV: Gas hourly space velocity
CIO: Cyclization isomerization oligomerization
**Symbols**
C_A0_: Concentration (mol m^–3^)
D: Mass diffusivity (m^2^ s^–1^)
D_a_: Damköhler number
Fr: Froude number
g: Gravity (m s^–2^)
h: Convective heat transfer coefficient (W m^–2^ K^–1^)
k: Thermal conductivity (W m^–1^ K^–1^)
Km: Mass transfer coefficient (m s^–1^)
K_rxn_: Reaction constant (m^3^ mol^–1^)^n–1^ s^–1^
L: Characteristic length (m)
n: Reaction order
Nu: Nusselt number
Pe_m_: Peclet number of mass transfer
Pe_h_: Peclet number of heat transfer
Re: Reynolds number
Sc: Schmidt number
Sh: Sherwood number
u: Velocity (m s^–1^)
**Greek Letters**
α: Thermal diffusivity (m^2^ s^–1^)
ε: Porosity
μ: Dynamic viscosity (kg m^–1^ s^–1^)
ρ: Density (kg m^–3^)
